# Supplementary material for: Iron absorption and loss, and efficacy of iron supplementation with and without prebiotics in children with virally suppressed HIV: three prospective studies in South Africa
Source: Nat Commun. 2025 Nov 13;16:9929. doi: 10.1038/s41467-025-64998-4 (PMC12615833; doi:10.1038/s41467-025-64998-4)
Supplement: Supplementary file 1 — Supplementary Information [file 41467_2025_64998_MOESM1_ESM.pdf]

Iron absorption and loss, and efficacy of iron supplementation with and without prebiotics in children with virally suppressed HIV: three prospective studies

SUPPLEMENTARY MATERIAL

Antiretroviral regimens of the children living with HIV in the three studies

In Study 1, the n=43 children with HIV were on the following antiretroviral regimens: n=23 were taking Abacavir-Lamivudine-Lopinavir boosted with Ritonavir; n=14 were taking Abacavir-Lamivudine-Efavirenz; n=5 were taking Zidovudine-Lamivudine-Lopinavir boosted with Ritonavir; and n=1 was taking Tenofovir-Emtricitabine-Lopinavir boosted with Ritonavir. In Study 2, of the 29 children with HIV, 20 were taking Abacavir-Lamivudine-Lopinavir boosted with Ritonavir, and 9 were taking Abacavir-Lamivudine-Efavirenz. In Study 3, of the 83 children with HIV included in the analysis: 8 were taking Abacavir-Lamivudine-Lopinavir boosted with Ritonavir, 7 were taking Abacavir-Lamivudine-Atazanavir boosted with Ritonavir, 4 were taking Abacavir-Lamivudine-Efavirenz, 20 were taking Abacavir-Lamivudine-Dolutegravir, 1 was taking Abacavir-Dolutegravir, 1 was taking Zidovudine-Abacavir-Lamivudine-Dolutegravir, 2 were taking Zidovudine-Lamivudine-Dolutegravir, 2 were taking Zidovudine-Lamivudine, 13 were taking Zidovudine-Lamivudine-Lopinavir boosted with Ritonavir, 1 was taking Dolutegravir-Darunavir boosted with Ritonavir, 2 were taking Lamivudine-Dolutegravir-Tenofovir-Darunavir boosted with Ritonavir, 23 were taking Lamivudine-Dolutegravir-Tenofovir, and 2 were taking Tenofovir-Emtricitabine-Lopinavir boosted with Ritonavir.

Statistical analyses

We performed the statistical analyses using the R statistical programming environment (R version 4.0.2) and SPSS (IBM). Values in the text and in tables are presented as means ± SDs for normally distributed data, and as medians (inter-quartile ranges [IQR]) for non-normally distributed data. When data were not normally distributed, appropriate transformation of values was performed before statistical analysis. In Studies 1 and 2, we used (two-sided) independent-samples t-tests to compare continuous variables between children with and without HIV. To compare categorical variables between children with and

without HIV, we used Pearson's Chi-squared tests or Fisher's exact tests. In Study 2, we used repeated-measures ANOVA to determine differences in variables assessed across three study time points between children with and without HIV. In Study 1, we further performed multiple linear regression models to explore predictors of FIA and hepcidin. In Study 3, we determined the effects of GOS intervention using linear-mixed models (LMM) with the outcome variable of interest as dependent variable, group and time as fixed effects, study IDs as random effects, and sex and age as covariates. We further compared the proportion (%) of children who had at least one episode of self-reported respiratory and gastro-intestinal symptoms and side effects during the intervention period between the two treatment groups using logistic regression analysis, adjusted for age and sex (expressed as Odds ratio [OR] with 95% confidence interval [CI]). The incidence (episodes) with symptoms, expressed as absolute number of episodes and as proportion (%) of the total number of episodes in each treatment group, was compared between the two treatment groups using Poisson regression with log linear link, adjusted for age and sex (expressed as Incidence Rate Ratio [IRR] with 95% CI). Results with *P* values between 0.05 and 0.08 were considered indicative of a trend and are reported as such. No adjustments were made for multiple comparisons, as primary and secondary outcomes were pre-specified and interpreted within a hierarchical framework.

Study 1

Supplementary Table 1. Study 1: Socio-economic and demographic characteristics of South African children without and with virally suppressed HIV

	**Children with HIV**	**Children without HIV**	*P* value[1]
n	43	45	
Access to school nutrition programme, n (%)	40 (93)	38 (85)	0.176
Formal / informal housing, n (%)	26 (61) / (41)	31 (69) / 14 (31)	0.684
Number of household members, median (IQR)	5 (4–6)	5 (4–7)	0.171
Primary caregiver highest level of education, n (%)			0.299
College/University	3 (7)	3 (7)	
Grade 10–12	22 (51)	31 (69)	
Grade 8–9	8 (19)	4 (9)	
Primary school or less	10 (19)	7 (16)	
Primary caregiver single / in partnership, n (%)	25 (58) / 18 (42)	27 (60) / 18 (40)	0.185
Employment status of household breadwinner, n (%)			0.042
Permanently employed	13 (30)	17 (42)	
Temporarily employed	10 (23)	4 (9)	
Unemployed	20 (47)	22 (49)	

[1]Independent-samples t-tests (two-sided) were used to compare continuous variables (non-normally distributed variables were log-transformed for analysis), and Pearson's Chi-squared tests (two-sided) were used to compare categorical variables between the two groups. No adjustments were made for multiple comparisons.

Supplementary Table 2. Study 1: Models predicting fractional iron absorption (%) from iron-fortified maize porridge, a lipid-based nutrient supplement (LNS), and an oral iron supplement in iron-depleted South African children without HIV and with virally suppressed HIV (*n*=87)

	Model 1 (without HIV variable)			Model 2 (with HIV variable)		
Variable	B	95% CI	P	B	95% CI	P
Maize porridge, %[1]						
Age, y	0.848	0.185, 1.511	0.013	0.857	0.187, 1.528	0.013
SF, μg/L[2]	−0.057	−0.178, 0.065	0.355	−0.057	−0.179, 0.066	0.359
sTfR, mg/L	−0.027	−0.421, 0.366	0.891	−0.019	−0.419, 0.381	0.924
AGP, g/L	−3.353	−7.521, 0.815	0.113	−3.209	−7.522, 1.103	0.142
Hepcidin, ng/mL	0.043	−0.130, 0.216	0.623	0.039	−0.137, 0.215	0.659
HIV				−0.300	−2.398, 1.799	0.777
adjusted R^2	0.067			0.056		
LNS, %[3]						
Age, y	1.072	0.506, 1.638	<0.001	1.054	0.485, 1.623	<0.001
SF, μg/L[2]	−0.041	−0.151, 0.068	0.453	−0.041	−0.151, 0.068	0.454
sTfR, mg/L	−0.074	−0.406, 0.258	0.658	−0.093	−0.429, 0.243	0.581
AGP, g/L	−2.316	−5.857, 1.225	0.196	−2.631	−6.265, 1.003	0.153
Hepcidin, ng/mL	−0.122	−0.293, 0.049	0.160	−0.105	−0.282, 0.072	0.242
HIV				0.737	−1.084, 2.559	0.422
adjusted R^2	0.207			0.203		
Iron supplement, %[4]						
Age, y	0.078	−6.378, 6.534	0.981	−0.072	−6.608, 6.464	0.983
SF, μg/L[2]	−0.070	−0.282, 0.141	0.510	−0.068	−0.282, 0.145	0.526
sTfR, mg/L	0.734	0.019, 1.450	0.044	0.762	0.030, 1.495	0.042
AGP, g/L	4.285	−8.277, 16.847	0.499	4.627	−8.122, 17.376	0.472

Hepcidin, ng/mL	−0.507	−1.087, 0.073	0.086	−0.536	−1.138, 0.065	0.079
HIV				−0.811	−4.780, 3.158	0.685
adjusted R^2	0.285			0.277		

The estimated standard coefficient (B) and 95% CI for unadjusted FIA from the maize porridge, LNS, and the iron supplement were assessed using two-sided multiple linear regression models.

For FIA from the maize porridge and the LNS, predictor values were obtained at the baseline visit. For the FIA from the iron supplement, predictor values were averaged across baseline and Day 31 (including PF, sTfR, AGP and hepcidin). No adjustments were made for multiple comparisons.

Abbreviations: AGP, α-1-glycoprotein; $FeSO_4$, ferrous sulfate; FIA, fractional iron absorption; LNS, lipid-based nutrient supplement; SF, plasma ferritin; sTfR, soluble transferrin receptor.

[1]*n* = 83.

[2]SF adjusted for C-reactive protein and AGP.[1]

[3]*n* = 79.

[4]*n* = 78.

Supplementary Table 3. Study 1: Models predicting baseline hepcidin (ng/mL) in iron-depleted South African children without HIV and with virally suppressed HIV (*n*=87)

	Model 1 (without HIV variable)			Model 2 (with HIV variable)		
Variable	B	95% CI	P	B	95% CI	P
Age, y	−0.692	−1.454, 0.069	0.074	−0.659	−1.420, 0.102	0.089
Hb, g/L	0.034	−0.079, 0.147	0.549	0.025	−0.089, 0.139	0.663
SF, μg/L[1]	0.128	−0.018, 0.274	0.084	0.128	−0.017, 0.274	0.083
sTfR, mg/L	−0.540	−1.014, −0.066	0.026	−0.492	−0.971, −0.017	0.044
AGP, g/L	1.458	−4.119, 7.035	0.604	2.078	−3.574, 7.729	0.467
IL-6, pg/mL	1.422	0.465, 2.379	0.004	1.372	0.415, 2.330	0.006
HIV				−1.511	−3.956, 0.945	0.224
adjusted R^2	0.203			0.208		

The estimated standard coefficient (B) and 95% CI for baseline hepcidin were assessed using two-sided multiple linear regression models. Predictor values were obtained from baseline visit. No adjustments were made for multiple comparisons.

Abbreviations: AGP, α-1-acid glycoprotein; Hb, haemoglobin; IL-6, interleukin-6; PF, plasma ferritin; sTfR, soluble transferrin receptor.

[1]PF adjusted for C-reactive protein and AGP.[1]

Study 2

Food frequency questionnaire

At the beginning of the equilibration period, a single dietitian interviewed the child-caregiver pair to assess dietary intake over the previous month using a quantified food frequency questionnaire (aQFFQ) and portion size estimation kit, as previously described.[2] The aQFFQ food list included fortified wheat and maize products, fibre-rich grains and cereals, nuts, fruit, vegetables, legumes, dairy, eggs, and animal flesh products that reflected the study population’s habitual intake. The food list excluded basic non-fortified

low-fibre staples (such as pasta and white rice), sugar and sugary foods, fats, oils, and salt. The aQFFQ could therefore estimate intake of protein, fibre, and selected micronutrients. Household measures were quantified according to the Food Quantities Tables for South Africa[3] and coded using the Food Composition Tables for South Africa.[4] Consumption for the past month (28 days) was reported. Frequency reporting was done as number of times per day, per week, or per month. Portion size estimation was facilitated with food portion photographs, various crockery and household utensil sizes, and pre-weighed food examples specific to the aQFFQ. The aQFFQ was interview-administered, once-off, by a single trained nutrition professional with the child-caregiver pair. Interviews were conducted in the child and caregiver's preferred language and where necessary, a translator from the research site assisted. Dietary intakes in children without HIV and with virally suppressed HIV are shown in **Supplementary Table 5**.

Supplementary Table 4. Study 2: Socio-economic and demographic characteristics of South African children without and with virally suppressed HIV

	Children with HIV	Children without HIV	*P* value[1]
n	29	36	
Access to school nutrition programme, n (%)	21 (81) / 5 (19)	23 (64) / 13 (36)	0.148
Formal / informal housing, n (%)	20 (69) / 9 (31)	20 (56) / 16 (44)	0.269
Number of household members, median (IQR)	5 (4–7)	6 (5–8)	
Primary caregiver highest level of education, n (%)			0.132
College/University	0 (0)	2 (6)	
Grade 10–12	18 (62)	28 (78)	
Grade 8–9	7 (24)	5 (14)	
Primary school or less	5 (17)	1 (3)	
Primary caregiver single / in partnership, n (%)	12 (41) / 17 (59)	11 (31) / 25 (69)	0.364
Employment status of household breadwinner, n (%)			0.062
Permanently employed	5 (17)	16 (45)	
Temporarily employed	6 (21)	6 (17)	
Unemployed	18 (62)	14 (39)	

[1]Independent-samples t-tests (two-sided) were used to compare continuous variables (non-normally distributed variables were log-transformed for analysis), and Pearson's Chi-squared tests (two-sided) were used to compare categorical variables between the two groups. No adjustments were made for multiple comparisons.

Supplemental Table 5. Study 2: Dietary intakes of iron, types of iron, and factors influencing dietary iron bioavailability in South African children with virally suppressed HIV and without HIV

	Children with HIV	Children without HIV	P value[1]
N	29	36	
Total iron, mg	16.1 (14.2–20.8)	18.1 (14.4 –20.2)	0.692
Non-haem iron, mg	13.3 (11.4–17.1)	13.1 (10.2–16.1)	0.153
Fortification iron, mg	8.8 (7.0–13.0)	5.7 (4.1–9.3)	0.005
Haem iron, mg	2.4 (1.6–3.6)	4.3 (3.2–5.9)	0.001
Animal protein, g	31.0 (26.1–39.9)	58.0 (42.3–71.7)	<0.001
Plant protein, g	34.4 (28.0–42.8)	34.6 (24.1–41.0)	0.364
Phytate, mg	302.0 (105.1–421.5)	266.2 (186.9–379.9)	0.183
Vitamin C, mg	51.1 (39.1–79.1)	92.9 (65.2–169.1)	0.002
Vitamin A, μg RAE	556.3 (354.1–851.9)	811.3 (414.7–1376.5)	0.411

Values are medians (IQR).

[1]Independent-samples t-tests (two-sided) were used to compare log-transformed variables. No adjustments were made for multiple comparisons.

Stable isotope dilution isotopic analyses

Calculation of total body iron (Fe_{total})[5]

The first step in the isotope dilution method is calculation of total body iron (Fe_{total}), which is calculated as the sum of circulating iron, tissue iron, and storage iron according to the following equation:

$$Fe_{total} = Fe_{circ} + Fe_{tissue} + Fe_{store}$$

where: Fe_{circ} represents circulating iron, and is calculated according to the equation:

Circulating Fe (mg) = haemoglobin (g/L) × blood volume (L) × 3.47 (mg Fe / g Hb)

Blood volume for children is assumed to be 0.065 L/kg body weight.[6]

Fe_{tissue} represents tissue iron in myoglobin and iron-containing enzymes and is simplified as 6 mg/kg body.[6]

Fe_{store} represents storage iron, consisting of iron in ferritin and hemosiderin and for adults and children is calculated (if serum ferritin concentration is >15.9 µg/L) according to the equation:[7]

Fe_{store} =(9380 x log(serum $ferritin_A$ [µg/L]) – 11,260) / 1000 x body weight]

where: serum $ferritin_A$ is serum ferritin concentration adjusted for inflammation according to the BRINDA initiative.[8]

Calculation of ^{57}Fe tracer concentration

After ^{57}Fe administration, the isotope concentration in blood sharply decreases after an initial enrichment shift due to recycling of iron from senescent red blood cells into other tissues. After approximately 8 months in children,[6] complete isotopic equilibration is established, indicated by a linear slope of the natural logarithm of the tracer concentration plotted against time.

Taking into account that stable isotope tracers are not monoisotopic, the measured isotopic ratio in the analyzed blood sample $R_{57/56}$ can be expressed as follows:[5]

$$R_{57/56} = \frac{a_{nat}^{57} \times n_{nat} + a_{A}^{57} \times n_{A}}{a_{nat}^{56} \times n_{nat} + a_{A}^{56} \times n_{A}}$$

where a_{nat}^{57} represents the concentration of the isotope ^{57}Fe in natural iron, n_{nat} the amount in mol of natural iron, a_{A}^{57} the concentration of the isotope ^{57}Fe in the enriched tracer, and n_A the amount in mol of tracer.

The tracer concentration ${}_{a}^{A}A^{t}$ in circulation at time t is represented by:

$$ {}^{A}_{a}A^{t} = {}^{n_A}/_{n_{tot}} = \frac{a^{57}_{nat} - a^{56}_{nat} \times R_{57/56}}{(a^{56}_{tracer} - a^{56}_{nat}) \times R_{57/56} + a^{57}_{nat} - a^{57}_{tracer}} $$

with n_A the amount in mol of tracer (here ^{57}Fe) and n_{total} the amount in mol of all iron in circulation.

Calculation of iron absorbed, lost and gained[5]

The decrease in tracer concentration after the equilibration period, in which all body iron is equilibrated with the tracer, reflects the rate of change of body iron composition [d^{-1}] expressed as:

$$ \frac{d({}^{A}_{a}A)}{dt} = -k_{abs} \times {}^{A}_{a}A $$

where: $d(^{A}A)/dt$ represents the rate of change of tracer concentration ^{A}A per unit of time, k_{abs} is the rate of change of tracer concentration constant.

Resolution of the differential equation leads to:

$$ {}^{A}_{a}A(t) = {}^{A}_{a}A_0 \times e^{-k_{abs} \times t} \quad \text{or} \quad \ln({}^{A}_{a}A_t) = -k_{abs} \times t + \ln({}^{A}_{a}A_0) $$

Iron loss only reduces the amount of body iron but does not affect its isotopic composition. Thus k_{loss} as the rate of decrease of isotopic label results from the semi-logarithmical regression of the tracer amount plotted against time; similar to the relationship shown above for k_{abs}.

The mean quantity of iron absorbed (Fe_{abs}) over the period of interest is then calculated as

$Fe_{abs} = -k_{abs} \times Fe_{total}$

where: Fe_{total} is the total body iron at the period midpoint calculated from the linear regression of Fe_{total} against time, k_{abs} is the rate of change of tracer concentration constant.

Mean iron loss (Fe_{loss}) is calculated correspondingly and net iron balance (Fe_{gain}) is determined by subtracting Fe_{loss} from Fe_{abs}.

Study 3

qPCR analysis of selected gut bacteria

We targeted *Bifidobacterium spp.*, as they are main targets of specific prebiotics such as GOS. We further measured virulence and toxin genes of six enteropathogenic bacteria: Enteropathogenic *Escherichia coli* (EPEC), Enterotoxigenic *Escherichia coli* (ETEC), *Salmonella ssp, Clostridium perfringens, Clostridium difficile and Campylobacter spp.* Finally, we measured the abundance of total bacteria by targeting the universal small subunit of the ribosome (henceforth, 16S rRNA). Primer details are given in **Supplementary Table 6.** DNA was extracted from 250 mg (± 10%) of feces with the use of a FastDNA Spin Kit for Soil (MP Biomedicals). Quantification was carried out with a Nanodrop ND-1000 Spectrophotometer. (Witec AG) at 260 nm. qPCR was performed with BioMark 96.96 Gene Expression Dynamic Arrays (Fluidigm). A sample premix was prepared by mixing 2 µl DNA with 0.4 µl 20 × DNA Binding Dye Sample Loading Reagent (PN 100–0388; Fluidigm), 4 µl 2 × Taq Man Gene Expression Master Mix (PN 4359016; Thermo Fisher Scientific), 0.4 µl 20 × EvaGreen DNA binding dye (PN 31000; Biotium), and 1.2 µl Tris-EDTA buffer. We prepared the assay mix by mixing 4 µl of 2 × Assay loading Reagent (PN 85000736; Fluidigm), 0.4 µl Tris-EDTA buffer, and 3.6 µl of the 20 µmol/l of a forward and reverse primer mix. Dynamic Array chips (PN BMK-M-96.69; Fluidigm) on a BioMark system were loaded and run as described by the manufacturer (advanced development protocol 41: using EvaGreen DNA-binding dye for gene expression with the 48.48 and 96.96 Dynamic Array IFCs; Fluidigm). The PCR was performed under the following conditions: 50 °C for 2 min and 95 °C for 10 min followed by 40 cycles at 95 °C for 15 s and at 60 °C for 1 min. To generate standards, complete or partial 16S ribosomal RNA genes were amplified from representative strains. Amplicons were purified and cloned into a PGEMT Easy Vector (Promega) and heterologously expressed in *Escherichia coli* according to the instruction of the supplier. Standard curves were prepared from 10-fold dilutions of linearized plasmids harbouring the 16S ribosomal RNA gene of interest. Concentrations were measured with a Qubit dsDNA BR assay kit (Q32850; Thermo Fisher Scientific) in triplicate on a Spark M10 plate reader (Tecan Group Ltd.). Data were processed with Fluidigm Real-Time PCR Analysis Software, including a melting curve analysis, and expressed as log gene copies/total 16S rRNA gene copies.

Supplementary Table 6. Study 3: Primers of targeted bacteria for qPCR analysis in faecal samples.

Target Bacteria	PCR primer sequence 5'-3'	Product size (bp)	Reference
Total Bacteria	Eub338F ACTCCTACGGGAGGCAGCAG Eub518R ATTACCGCGGCTGCTGG	200	9
Bifidobacterium spp.	Bif F TCGCGTCYGGTGTGAAAG<br>Bif R CCACATCCAGCRTCCAC	243	10
Campylobacter spp.	Camp-fwd CTGCTTAACACAAGTTGAGTAGG<br>Camp-rev TTCCTTAGGTACCGTCAGAA	287	11
Clostridium difficile	cdF TTGAGCGATTTACTTCGGTAAAGA<br>cdR CCATCCTGTACTGGCTCACCT	157	10
Clostridium perfringens	plcF AAGTTACCTTTGCTGCATAATCCC<br>plcR ATAGATACTCCATATCATCCTGCT	283	12
Salmonella ssp.	invA, 139 GTGAAATTATCGCCACGTTCGGGCAA<br>invA, 141 TCATCGCACCGTCAAAGGAACC	284	13
Enteropathogenic Escherichia coli (EPEC)	EAE-a ATGCTTAGTGCTGGTTTAGG<br>EAE-b GCCTTCATCATTTCGCTTTC	248	13
Enterotoxigenic Escherichia coli (ETEC)	LT-1 AGCAGGTTTCCCACCGGATCACCA<br>LT-2 GTGCTCAGATTCTGGGTCTC	132	13

References for Supplementary Material

1. Namaste SM*, et al.* Adjusting ferritin concentrations for inflammation: Biomarkers Reflecting Inflammation and Nutritional Determinants of Anemia (BRINDA) project. *Am J Clin Nutr* **106**, 359S-371S (2017).

2. Goosen C, Blaauw R. The Development of a Quantified Food Frequency Questionnaire for Assessing Iron Nutrition in Schoolchildren from Resource-Limited Settings in Cape Town, South Africa. *Ecol Food Nutr* **60**, 707-721 (2021).

3. South African Food Data System (SAFOODS). *SAMRC Food Quantities Manual for South Africa*, 3rd edn. SAMRC (2018).

4. South African Food Data System (SAFOODS). *SAMRC Food Composition Tables for South Africa*, 5th edn (2017).

5. Stoffel NU, Zeder C, Zimmermann MB. Assessing Human Iron Kinetics Using Stable Iron Isotopic Techniques. *Clin Pharmacokinet* **63**, 1389-1405 (2024).

6. Fomon SJ, Nelson SE, Serfass RE, Ziegler EE. Absorption and loss of iron in toddlers are highly correlated. *The Journal of nutrition* **135**, 771-777 (2005).

7. Hallberg L, Hulthén L, Garby L. Iron stores in man in relation to diet and iron requirements. *Eur J Clin Nutr* **52**, 623-631 (1998).

8. Namaste SML, Ou J, Williams AM, Young MF, Yu EX, Suchdev PS. Adjusting iron and vitamin A status in settings of inflammation: a sensitivity analysis of the Biomarkers Reflecting Inflammation and Nutritional Determinants of Anemia (BRINDA) approach. *Am J Clin Nutr* **112**, 458s-467s (2019).

9. Fierer N, Jackson JA, Vilgalys R, Jackson RB. Assessment of soil microbial community structure by use of taxon-specific quantitative PCR assays. *Appl Environ Microbiol* **71**, 4117-4120 (2005).

10. Rinttilä T, Kassinen A, Malinen E, Krogius L, Palva A. Development of an extensive set of 16S rDNA-targeted primers for quantification of pathogenic and indigenous bacteria in faecal samples by real-time PCR. *J Appl Microbiol* **97**, 1166-1177 (2004).

11. Josefsen MH, Jacobsen NR, Hoorfar J. Enrichment followed by quantitative PCR both for rapid detection and as a tool for quantitative risk assessment of food-borne thermotolerant campylobacters. *Appl Environ Microbiol* **70**, 3588-3592 (2004).

12. Rinttilä T, Lyra A, Krogius-Kurikka L, Palva A. Real-time PCR analysis of enteric pathogens from fecal samples of irritable bowel syndrome subjects. *Gut Pathog* **3**, 6 (2011).

13. Fukushima H, Tsunomori Y, Seki R. Duplex real-time SYBR green PCR assays for detection of 17 species of food- or waterborne pathogens in stools. *J Clin Microbiol* **41**, 5134-5146 (2003).

Application Form for the Ethics Commission of ETH Zurich

A. General Information

Project Title

A novel stable iron isotope method to define iron needs and improve iron nutrition in HIV+ and HIV- children

Principle investigator

Name	Title	Group	University
Michael Zimmermann	Prof. MD.	Human Nutrition Laboratory	ETH Zurich

Involved collaborators

Name	Title	Group	University
Mark Cotton	Prof. MD.	FAM-CRU, Department of Pediatrics and Child Health	Stellenbosch University Faculty of Medicine and Health Sciences
Renée Blaauw	Prof. PhD	Human Nutrition Division	Stellenbosch University Faculty of Medicine and Health Sciences
Shaun Barnabas	Dr. MD	FAM-CRU, Department of Pediatrics and Child Health	Stellenbosch University Faculty of Medicine and Health Sciences
Steve Innes	Dr. MD	FAM-CRU, Department of Pediatrics and Child Health	Stellenbosch University Faculty of Medicine and Health Sciences
Charlene Goosen	PhD candidate	Human Nutrition Division	Stellenbosch University Faculty of Medicine and Health Sciences
Colin Cercamondi	Dr. PhD	Human Nutrition Laboratory	ETH Zurich
Nadja Mikulic	PhD candidate	Human Nutrition Laboratory	ETH Zurich

Number of participants	***Duration of the study***	***Type of project***
Minimum: 160	Begin: September, 2018	☑ Research Project
Maximum: 180	End: February, 2020	☐ (Master Thesis)

B. Scientific Information

1. Abstract

Background: In Sub-Saharan Africa, HIV is a major cause of morbidity and mortality in children. Anemia frequently complicates pediatric HIV infection and predicts disease progression and mortality. Iron requirements and the specific contribution of iron deficiency (ID) to anemia in pediatric HIV infection remains uncertain. The fundamental barrier to understanding iron nutrition in HIV infection is that subclinical inflammation in individuals with HIV infection confounds the usual biomarkers used to assess iron status and response to iron interventions. A novel iron stable isotope technique developed by ETH Zurich, Switzerland, is a promising new tool for better understanding of iron metabolism in HIV infection. In contrast to existing conventional biomarkers of iron status, a method based on isotopic dilution of whole body iron labeled with stable, non-radioactive isotopes of iron (^{58}Fe, ^{57}Fe) could directly quantify iron requirements, as well as iron absorption from interventions, completely free of bias and confounding by inflammation. This method could offer, for the first time, a long-term quantitative measure of iron balance and absorption from iron interventions and provide reliable data on which to base nutrition recommendations for HIV infection.

Objectives: Comparing HIV infected children to uninfected children: 1) Quantify iron absorption from iron fortified maize porridge, lipid-based food supplements and oral iron supplements; 2) Quantify the daily iron requirement.

Design: The study participants will be recruited from the South African Stellenbosch University/Tygerberg Children's Hospital long-term antiretroviral therapy (ART) cohort of perinatally HIV infected children and uninfected controls from the same communities. In study 1, using a randomized cross-over design and stable isotope labeled single meal/doses we will: a) quantify the impairment of dietary iron absorption in HIV infected, iron deficient children compared to HIV uninfected, iron deficient controls using a labeled iron fortified maize meal, a lipid-based nutritional supplement (LNS) and an oral iron supplement; and b) administer sufficient iron isotope label (^{57}Fe) to allow equilibration and follow up of isotopic composition in the blood for two years (isotope dilution technique). At the end of Study 1, all iron deficient children will be iron replete prior to entering Study 2. In study 1, in parallel, a group of HIV infected and uninfected, iron sufficient children will be given orally 12 mg ^{57}Fe as ferrous sulfate ($FeSO_4$). In study 2, we will apply the principle of long-term isotope dilution to quantify the daily iron requirement in both the HIV infected and uninfected children, and the difference in iron requirements.

Significance and overall goal: To provide optimized recommendations on dietary iron requirements and iron treatment regimens in HIV infected children, in order to reduce ID and anemia, improve their health and well-being, their long-term prognosis and quality of life.

2. Project

a. Goals of the project with references to one's own and other published data as well as the results of any preliminary investigations

In Sub-Saharan Africa, HIV is a major cause of mortality and morbidity among children. It is estimated that in Sub-Saharan Africa there are ≈3 million HIV infected children <15 years of age of whom ≈330'000 live in South Africa. South Africa has an estimated 5.6 million HIV infected-individuals, more than any other country [1]. Worldwide, more individuals have iron deficiency (ID) than any other nutritional problem [2] and similar to HIV, the highest burden of iron deficiency anemia (IDA) is in Sub-Saharan Africa [3]. IDA impairs cognitive development in children [4].

The dual burden of anemia and HIV in Sub-Saharan Africa has substantial health and economic costs [5] with the relationship between these two, often-overlapping disorders, complex and poorly understood [6]. Anemia is an independent marker for increased disease progression and death in HIV infected children [7, 8] and is common before antiretroviral therapy (ART) is initiated. Although ART may reduce the prevalence of anemia, many patients fail to resolve or develop anemia, after ART initiation [9]. While anemia frequently complicates pediatric HIV infection [9, 10] with a Cape Town study of HIV infected toddlers reporting a prevalence of 73% [11] the difficulties of defining iron status during inflammation make it difficult to accurately estimate the contribution of ID to anemia during HIV [12]. The pathophysiology of HIV-associated anemia includes decreased dietary iron absorption, increased iron turnover, increased iron sequestration in the reticuloendothelial system and ineffective erythropoiesis [13].

During infections, iron metabolism is carefully regulated: iron is tightly bound in the intra- and extracellular compartments to reduce oxidative damage and limit its availability to potential pathogens [14]. This effect is mediated by inflammation and is protective during acute infections. However, during chronic infections, protracted inflammation has detrimental effects on iron status and hemoglobin (Hb). Chronic immune activation and inflammation are characteristic of both untreated and treated HIV infection [15, 16]. During HIV infection, increases in interleukin (IL)-6, IL-22, and type I interferon (IFN) stimulate hepatic synthesis of hepcidin which mediates redistribution of iron within the body [16], a process that becomes more pronounced with HIV disease progression [17, 18]. Upregulated hepcidin inactivates ferroportin decreasing intestinal iron absorption and increasing the sequestration of iron in macrophages [14], limiting erythropoiesis [16, 19] and causing anemia [20, 21]. In adults, hepcidin remains significantly up regulated (roughly twofold) during chronic HIV infection, in concert with other inflammatory proteins [16]. Greater inflammation, higher hepcidin levels and impairments in iron metabolism predict a more rapid progression of HIV disease [22].

The contribution of ID to anemia in pediatric HIV infection remains uncertain [23]. Several studies have reported impairments in iron metabolism and ID in children with HIV. In a prospective study in low-income, HIV infected South African children (n=58, 3 to 14 y-old) enrolled pre-ART and followed for 18 months, the incidence of ID (defined by a high soluble transferrin receptor (sTfR)) increased from 15.2% at baseline to 37.2%. This increase was independent of age and socioeconomic status suggesting that despite ART, dietary iron intake (mean±SD, 6.2±2.8 mg/d) was inadequate to protect against ID [24]. Even subclinical inflammation associated with protracted infections can increase hepcidin and sharply reduce iron

absorption [25], thus iron fortification programs, like South Africa's, may have little benefit for HIV infected children.

ID in children may impair immune function [26]. Anemia predicts poor outcomes in an HIV infected population while correcting anemia prolongs survival [27, 28]. Interestingly, many individuals with advanced HIV disease have high iron stores that may be associated with more rapid disease progression and higher mortality [18, 29, 30], although not all studies agree [31]. A proposed mechanism for this is that high circulating hepcidin causes excess iron to deposit in the reticuloendothelial system, bone marrow, brain, liver and other organs [21] increasing oxidation and HIV replication [32], stimulating pathogen growth and impairing host immune responses [29, 33, 34]. It remains uncertain if increased iron stores are a cause or a consequence of disease progression [6].

It is unclear whether iron supplementation affects HIV infection disease prognosis and mortality [35]. Several short-term iron supplementation trials in HIV infected adults have reported reductions in anemia without an increase in viral load [36, 37], while other studies have suggested iron supplements may worsen co-infections and increase disease progression [6, 18]. Some recent studies have suggested that oral iron supplements and/or fortificants may increase gut inflammation in African children [38, 39] which may be particularly detrimental in HIV infected individuals, who may have gut immune activation, enteropathy and adverse shifts in the gut microbiome [40, 41]. Because of concerns around iron supplements in HIV infected adults [18], there is no international consensus about iron supplementation for HIV infected children. The current South African pediatric HIV management guidelines state that although all anemia should not be assumed to be IDA, a therapeutic 3-week trial of iron supplementation can be attempted in anemic children without further work up if Hb is measured at baseline and after 3 weeks to monitor the response [42]. Currently, routine iron supplementation is not recommended for HIV infected children in most Sub-Saharan African countries [43]. To our knowledge, there has been only one randomized controlled trial of iron supplementation, combined with multivitamins, in anemic HIV infected children. Iron supplementation improved Hb and immunity but also increased the frequency of malaria [35]. Current clinical practice is based on limited evidence and expert opinion [10]. Thus, whether iron supplementation and/or fortification is effective and safe in HIV infected children remains unclear.

A major barrier to judging the efficacy of iron interventions in HIV infected individuals is the limitations of conventional iron biomarkers in the face of chronic infection. The therapeutic dilemma of iron supplementation in anemia and HIV infection remains a major knowledge gap. The accurate assessment of iron status and longitudinal iron balance in HIV infected populations are currently not possible because the commonly used blood indices of iron status (plasma ferritin (PF), sTfR, plasma iron (PFe) are confounded by inflammation [13, 26, 44]. Hb has low sensitivity and specificity in detecting ID and relatively large amounts of iron may be required to produce a measurable increase in Hb [45]. Hepcidin can help interpret the interplay between anemia, iron status and inflammation [14] but its levels may be increased by infections during IDA. Thus, there is no reliable method to evaluate the potential efficacy of iron fortification and supplementation during chronic infections, such as HIV, and there is no internationally agreed methodology to define iron status in such groups [46]. This is a major challenge in global efforts to control anemia in HIV infected populations.

Studies using isotopic dilution of whole body iron labeled with stable, non-radioactive isotopes of iron (^{58}Fe, ^{57}Fe, ^{54}Fe) could directly quantify iron absorption from interventions completely free of bias and

confounding by infection or inflammation. This method could offer, for the first time, a long-term, accurate and quantitative measure of iron absorption and balance from iron interventions. Stable iron isotopic dilution techniques have been used to assess iron balance and requirements in U.S. children, but have never been employed in children in an area with a high infectious and inflammatory burden. After equilibration of an isotopic label in the body, the dilution of the label is solely dependent on the amount of unlabeled iron entering the body. Equilibration in adults takes 1 year, while in children, isotopic exchange with total body iron pools is complete in about 6-8 months [47]. A basis for the isotopic dilution method is the slow turnover of the erythrocyte-iron compartment, modulated mainly by the rate of erythrocyte renewal. After equilibration in the body of a stable iron isotope (*tracer*), dilution of the *tracer* in erythrocytes is exclusively dependent upon the amount of iron with natural isotopic composition (*tracee*) entering the body iron pool where a mixture of *tracee* and *tracer* is present. In other words, assuming that all the iron in the body has a homogeneous iron isotopic composition, the amount of absorbed dietary and supplemental iron entering the body at steady state can be assessed quantitatively by measuring isotopic enrichment of the *tracer* [48, 49].

By measuring the dilution of labeled body iron, an extremely precise measure of iron absorption (k_{abs}) is obtained with an estimated capacity to detect a difference in iron absorption between the iron-fortified and placebo groups of as little as 12.5 µg Fe/kg body weight/day (Zimmermann M., ETH Zurich internal data). Therefore, long-term labeling with stable isotopic techniques offers the opportunity to investigate fundamental and vital characteristics of iron metabolism, including long-term iron absorption, losses and iron requirements. The proposed project would, for the first time, apply the principle of long-term isotope dilution in assessing iron requirements and the efficacy of iron interventions in children with and without HIV infection. Furthermore, this method will allow a less burdensome (i.e., requiring fewer subject numbers per study), more accurate and cost-effective measurement of the efficacy and effectiveness of iron interventions. This could be a major advance over conventional study designs to monitor the effectiveness of iron interventions that require large numbers of subjects because of the inherent variability of blood biomarkers of iron status during chronic infections such as HIV. The current protocol includes two studies: 1) the measurement of iron bioavailability from iron fortified maize porridge, lipid-based food supplements and oral iron supplements in HIV infected children compared to uninfected children and 2) the determination of iron turnover and basal iron requirements in HIV infected and uninfected children.

The main study aims are to:

1) Quantify iron absorption from iron fortified maize porridge, lipid-based food supplements and oral iron supplements in HIV infected children compared to uninfected children.
2) Quantify the daily iron requirement in HIV infected children compared to uninfected children.

We hypothesize that:

1) In HIV infected children receiving ART, compared to uninfected controls:

a) Systemic inflammatory indices, gut inflammatory indices and plasma hepcidin (PHep) will be higher.
b) In iron deficient children, as quantified by a single test meal/supplement labeled with stable iron isotopes (^{57}Fe and ^{58}Fe), dietary iron bioavailability will be reduced from: i) an iron fortified maize-

based meal mimicking the South African fortification program; ii) an iron containing lipid-based nutritional supplement (LNS); and iii) an oral iron supplement.

c) The dose of iron isotopes (^{57}Fe) provided in (b) will, after an equilibration period of 8 months, uniformly label body iron in both groups of children.

d) Iron turnover over the subsequent 6 months, as measured by k_{abs}, the slope of ^{57}Fe isotopic dilution from the end of the baseline enrichment equilibration period will define the daily iron requirement of both groups of children, and the daily iron requirement will be greater in the HIV infected children.

b. Research plan

Project activity	Duration	Time
Ethical approvals obtained at both institutions, set-up	3 months	April, 2018 – June, 2018
Baseline assessment and recruitment complete	6 months	September, 2018 – March, 2019
Study 1	8 months	September, 2018 - April, 2019
Study 2 equilibration period	8 months	April, 2019– November, 2019
Study 2 turnover period	6 months	November, 2019 – April, 2020

c. Research methods

Study site and subjects

The Division of Pediatric Infectious Diseases at Stellenbosch University is one of the leading groups internationally working on HIV infection in children. The Division of Human Nutrition, Stellenbosch University, has a broad range of research focus areas including the public health significance of IDA in children. The Family Clinical Research Unit (FAM CRU) is located inside the Tygerberg Academic Hospital complex in Cape Town, and is adjacent to the Family Clinic for HIV. FAM-CRU has 1000m^2 research space for clinical trials and prospective cohort studies. It has over 100 full-time research staff including 15 nurses and 12 doctors. The participants in these studies will mainly be recruited from the FAM-CRU long-term ART birth cohort including:

- Perinatally HIV infected children 8-12 years old who initiated very early ART within the first few weeks after delivery (n=71).
- Perinatally HIV infected children 8-12 years old who initiated ART at 6-36 months of age (n=67).
- HIV-exposed uninfected (HEU) children from the same communities and socioeconomic background (n=68).
- HIV-unexposed uninfected (HU) children from the same communities and socioeconomic background (n=74).

- Newly recruited HIV infected and uninfected children 8-12 years from the same communities and socioeconomic background who are not from the FAM-CRU long-term ART birth cohort in Khayelitsha and Kraainfontein (n= depends on the number of included children from the FAM-CRU and the required sample size).

The FAM-CRU cohort undergoes extensive annual assessment and is relatively homogeneous and well characterized, having exited two local clinical trials a few years ago during which they were very carefully watched. They offer a unique and valuable opportunity to study and compare well-defined populations with substantial clinical relevance.

All children will undergo baseline investigations of their body weight, height, iron status, systemic and gut inflammation status from blood (7.5 mL):

- Iron status investigations will include full blood count (FBC) and isotopic iron composition of red blood cells in whole blood; and PF, sTfR, PFe, transferrin saturation (Tsat), total iron binding capacity (TIBC) and erythropoetin (EPO) in plasma.
- Systemic and gut inflammation status investigations will include highly sensitive C-reactive protein (hsCRP) and alpha-1-glycoprotein (AGP), PHep, IL-6, soluble cluster of differentiation 4 (sCD4) in HIV-infected children, intestinal fatty acid binding protein 2 (IFABP-2) and lipopolysaccharide binding protein (LBP) in plasma.

From this, body mass index (BMI), hematologic data (Hb), iron status (PF), and sCD4 and HIV RNA viral load (measured as part from routine care) as HIV progress estimators in HIV infected children will be used for screening parameters. If new children outside the FAM-CRU cohort need to be recruited, these HIV estimators will be tested as screening parameters using commercially available HIV rapid test kits. Baseline blood draw will be in the afternoon and children need to be fasted for 2 hours before the visit. Because iron parameters are likely to be influenced by the last meal, a short questionnaire will be conducted about the last meal.
Because of logistic reasons, we will not feed all children on one day. Therefore, we will have 15 groups of 12 children each. Each group will start on another day with study 1 and have an individual schedule. The above factors will be compared in the two groups of HIV infected children and uninfected children. Based on current data, iron depletion is estimated to affect approximately 50% of participants in the FAM-CRU cohort.

We expect that these measures will be comparable in the two groups of HIV infected children and comparable between the HEU and HU groups. Based on these findings, we will select **four groups of children**. The first two groups will be an **iron deficient** HIV infected group (n=45) and an **iron deficient** uninfected group (n=45).

The children (i.e. their caregivers) will be contacted, and upon obtaining written informed consent, which will be available in English, Afrikaans and IsiXhosa, they will be screened and then enrolled in the new project. All of the children will undergo two studies (see **Figure 1**). The other two groups will be an **iron sufficient** HIV infected group (n=45) and an **iron sufficient** uninfected group (n=45). After obtaining informed consent, these children will also undergo two studies, but study 1 will be simpler than for the iron deficient children (see **Figure 1**).

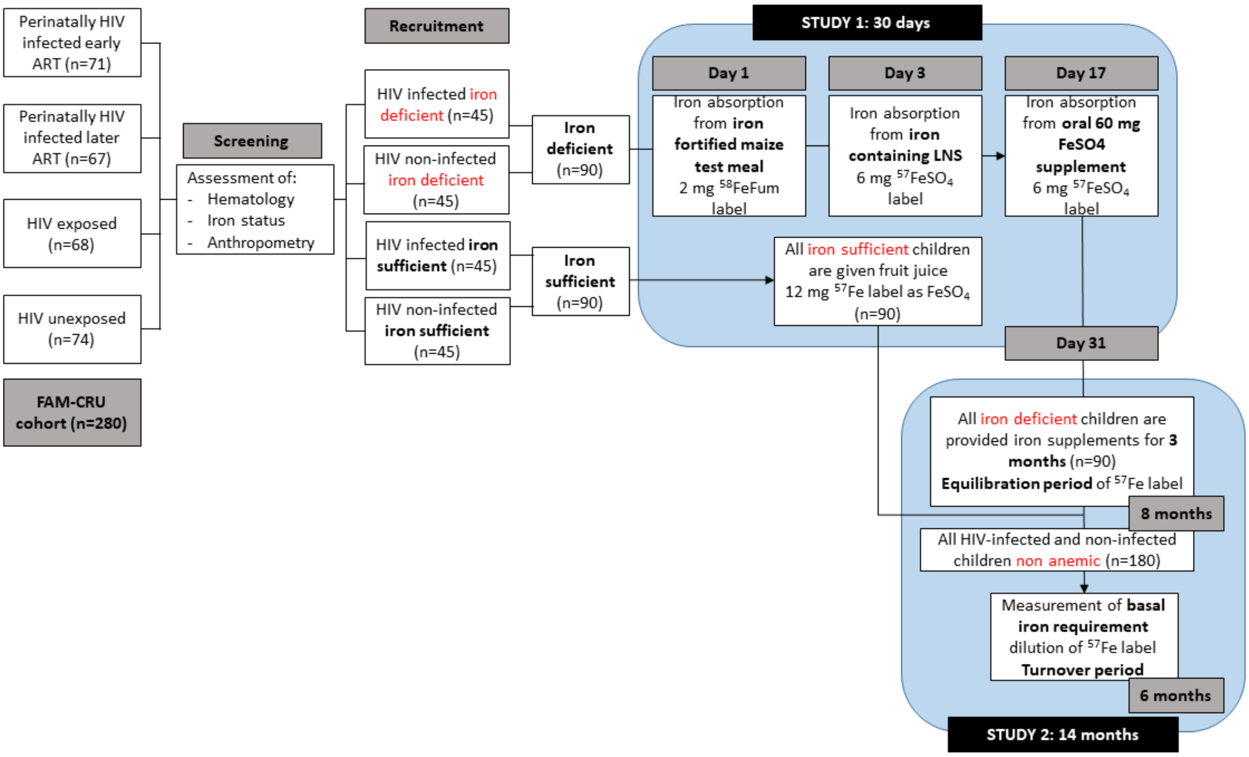

Figure 1: Study design

Study 1: Single meal/supplement stable iron isotope studies

Maize meal in South Africa (and in many other Sub-Saharan African countries) is fortified with iron to promote adequate iron intake in children. However, this may be so poorly absorbed in HIV infected children as to have a negligible effect on iron nutrition. Additionally, the cereal staple foods, like maize, depending on milling, may be high in phytic acid, a potent inhibitor of iron absorption [50]. Therefore, interventions with iron-containing LNS have been proposed for HIV infected individuals, but the iron bioavailability from the iron-fortified LNS has never been investigated and similarly, the absorption may be reduced in HIV infected children. In Study 1, we will therefore use iron stable isotopes for the first time in HIV infected, **iron deficient** children. In addition, the stable isotopes given in this study will be the prerequisite for the long term labeling required for study 2 and at a later stage also for the second phase of the project (separate protocol with new consent yet to be compiled).

Objectives:

1) Quantify the impairment in dietary iron absorption in HIV infected iron deficient children compared to uninfected iron deficient children from a labeled iron fortified maize meal (simulating the South African maize fortification strategy), a LNS and an oral iron supplement.
2) Investigate determinants of iron absorption in the two groups.
3) Administer sufficient iron isotope label to allow equilibration and use of isotope dilution techniques in the following two years (study 2 and second project phase).

On designated days 1 and 3, a randomized crossover design (the order of the two test meals will be randomly assigned for each subject) will be used with each subject in the two groups of children (HIV infected and uninfected, both iron deficient) serving as his/her own control and consuming the two different test meals. The two different test meals will consist of: 1) maize porridge extrinsically labeled with ferrous

fumarate (FeFum) (2 mg ^{58}Fe) as iron fortificant (the current compound used in the South African fortification program); and 2) a LNS (self-made; prepared in bulk every month) extrinsically fortified and labeled with ferrous sulfate ($FeSO_4$) (6 mg ^{57}Fe).

On days 1 and 3, the test meals, together with a glass of water (250 mL), will be administered in the presence of the investigators in the morning between 7 and 11 am after an overnight fast, where children should not eat from 10 pm on and not drink anything after midnight. The subjects will not be allowed to eat or drink for 3h after the meal. Afterwards, all children will receive a standardized meal for lunch (toasted cheese sandwich) on both test meal days. The stable iron isotopes will be purchased from Chemgas SA (France). The Paul Lohmann AG (Germany) will prepare the ^{58}Fe labeled FeFum and the Human Nutrition Laboratory (HNL) at ETH Zurich will prepare $^{57}FeSO_4$ from isotopic-enriched ^{57}Fe by dissolution in H_2SO_4 (food grade). FBC and Erythrocyte incorporation of ^{58}Fe and ^{57}Fe will be determined from a blood sample (3 mL) taken on day 17. Additionally, body weight and height will be measured on day 17.

Also on day 17, we will orally administer 170 mg iron tablets as $FeSO_4$ (containing 65 mg of elemental iron) with 6 mg extrinsically labeled ^{57}Fe to each iron deficient study child, together with a glass of water (225 mL) and fruit syrup (25 mL) to cover for the taste of iron. The children will arrive at the hospital after an overnight fast (no food from 10 pm on and no beverage after midnight), and will then consume the iron tablets after the blood sampling. There will be no intake of foods and fluids for 1h after the feeding. After 1h, children will receive a standardized small snack (white sugar bun with marmalade). Three hours after the test meal, all children will receive a standardized lunch (toasted cheese sandwich). Fourteen days later (day 31), we will collect again a venous blood sample (7.5 mL) to measure erythrocyte incorporation; iron status and systemic and gut inflammation status in plasma, and measure body weight, height and mid-upper arm circumference. Additionally, in the blood sample, plasma zinc status will be measured to compare nutritional status between HIV+ and HIV- children. Again, blood draw will be in the afternoon and children need to be fasted for 2h prior blood draw and a short questionnaire about the last meal/snack will be included. After completion of study 1, all children with ID will be treated with iron supplementation daily for 3 months. They will consume the above mentioned $FeSO_4$ iron tablets (65 mg elemental iron) or an oral iron syrup if they have problems to swallow a tablet daily for 3 months (per guidelines of Department of Health (2015) [42, 51]). These tablets and the iron syrup will not be isotopically labelled and the first day of the supplementation period is equal to the baseline of the 8 months equilibration period. Additionally, participants in the ID group will receive a gastrointestinal symptom diary for daily completion during the 3-month supplementation period.

In the additional groups of **iron sufficient** children (HIV infected and HIV uninfected), children will be labeled with 12 mg ^{57}Fe as $FeSO_4$ given in fruit juice on day 3. The administration of the isotopically labelled fruit juice will be done batch-wise. The children will arrive at the hospital after an overnight fast (no food from 10 pm on and no beverage after midnight), and will then consume the fruit juice. There will be no intake of foods and fluids for 1h after the feeding. Three hours after the test meal, all children will get a standardized lunch (toasted cheese sandwich). Subsequently, all the **iron sufficient** children will enter study 2 together with the **iron deficient** children on day 31; however, they do not receive the 3 months iron supplementation. On day 31, we will collect a venous blood sample (7.5 mL) to measure erythrocyte incorporation from the fruit juice; iron status and systemic and gut inflammation status in plasma; and measure body weight and height, together with the **iron deficient** children. Blood draw is in the afternoon

and children need to be fasted for 2h prior to blood draw and a short questionnaire about the last meal/snack will be included.

Both, the iron sufficient and deficient children will receive a total of 12 mg ^{57}Fe labels (single dose of 12 mg ^{57}Fe in a fruit juice vs. two times 6 mg ^{57}Fe from the labeled LNS and tablets). The isotopic labels will then equilibrate and completely label body iron during the subsequent equilibration phase.

During a face-to-face interview with the child and caregiver, relevant dietary information, including iron intake and iron bioavailability (by assessing iron absorption inhibitors (e.g. phytate) and enhancers (ascorbic acid)), assessed by quantitative food frequency questionnairs (QFFQ) adapted and validated to include local traditional food patterns by conducting 24h-recalls and group discussions with the population group will be recorded at day 3 and day 31 (for testing reproducibility of QFFQ from day 3) in a random half of the cohort subgroup. A food portion photograph book developed and tested for use in the African population together with different size crockery, glasses and household measure utensils will be used to assist participants with accurately estimating the portion sizes of foods eaten [52].

Maize test meal

The maize test meals will be prepared freshly on day 1 and 3. Unfortified maize flour will be purchased in bulk and used for the entire study. The maize test meal will contain 2 mg ^{58}Fe as FeFum. The $^{58}FeFum$ in powder form will be thoroughly mixed in a separate bowl with 20 g of porridge, which will be consumed prior to the main porridge meal. The test meals (70 mL cooked maize porridge per meal) will be prepared in the local traditional way and will consist of ~15 g unfortified maize flour (30% dry weight), 150 mL mineral water, a pinch of salt, 7.5 g white sugar and 40 mL full cream milk. Test meals will be consumed completely in the presence of the investigators and no intake of food and fluids will be allowed for 3 h after the test meal intake, except for test meal 3 on day 17 and the fruit juice test meal on day 3, where children will receive a standardized snack after 1h of test meals, otherwise they will be in a fasted state for too long. During this period, the children will be kept at the clinic under supervision. When the total amount of the meal is consumed, the bowl will be rinsed with 2x4 mL mineral water. The children will then drink the rinsing water. Start and end time of the meal consumption will be noted by the investigators.

Lipid-based nutritional supplementation

We will produce our own LNS at the study site batch-wise freshly every month (2-3 kg) (ingredients in **Table 1**). The LNS is storable over some months. Additionally, we will vacuum seal portion sizes in preformed aluminum sachets to increase storage durability. However, it will be tested for microbiological (coliforms 100 max., salmonella sp. absent in 25 g. E.coli absent in 1 g, total microorganism <100000 cfu/g max, yeast and molds 1000 max.) and toxicological safety (aflatoxin < 20 ppb). The formula will be adapted from a LNS previously used in one of our iron absorption studies [53]. Palm stearin will be used as a stabilizer to avoid the separation of the oil components and the solid components. The LNS (55 g) will be consumed together with a glass of water (250 mL). The 6 mg ^{57}Fe as $FeSO_4$ will be mixed in a separate bowl with 20 g of LNS, which will be consumed prior to the main LNS meal.

Table 1: Composition of the lipid-based nutritional supplement (LNS).

Ingredients	%
Canola oil	24
Peanut paste	23
Milk powder	23
Sugar	7
Maltodextrin	15
Palm stearin	8

Study 2: Defining daily iron turnover and the iron requirement in uninfected and HIV infected children based on iron isotope dilution

In Study 2, for the first time, we will apply the principle of long-term isotope dilution in assessing iron requirements in children with and without HIV infection. This method may provide the first accurate measurements of long-term dietary iron absorption and loss for estimation of iron requirements in these groups.

Objectives:

1) Achieve iron equilibration and labeling of body iron with ^{57}Fe.
2) Quantify the daily iron requirement in HIV infected and uninfected children, and compare their difference in iron requirements.
3) Determine the effect of oral iron supplementation on the gut microbiome, iron status and inflammation, and gut inflammation in HIV+ compared to HIV-, iron deficient children.
4) Characterize the gut microbiome diversity and abundance, and gut inflammation in iron deficient and sufficient children with and without HIV infection.

The dose of ^{57}Fe provided in Study 1 will, after an equilibration period of 8 months, uniformly label body iron in both groups of HIV infected and uninfected children. Iron turnover over the subsequent 6 months will be measured by using the slope of ^{57}Fe isotopic dilution from the baseline equilibration enrichment in both groups of children. This will allow quantification of the daily iron requirement in both the HIV infected and uninfected children, and the difference in iron requirements, as follows. Serial blood samples of small volume (7.5 mL) will be collected from all children 3 times during the equilibration period (baseline (day 31), 4 months, 8 months) and at 2 times (3 months and 6 months) during the subsequent measurement period of iron turnover. Blood draws are in the afternoon and children need to be fasted for 2h prior to blood draw and a short questionnaire about the last meal/snack will be included. Additionally, body weight and height will be measured, and mid-upper arm circumference on days 31 and 121 (before and after iron supplementation period).

Because the children in the FAM-CRU cohort are routinely assessed at 6-8 month intervals, including a venipuncture, we expect several of these venipunctures will be included as part of the routine care of the cohort, thereby minimizing blood draws.

To our knowledge, there is currently no data of the effect of routine iron supplementation (~65mg $FeSO_4$), a widely used therapeutic measure for correcting ID, on the gut microbiome in virally suppressed HIV

infected children, nor has the gut microbiome been characterized in older children with perinatal HIV infection and early onset ART, or in those with overlapping HIV infection and ID. Furthermore, this study provides a unique opportunity to compare the nutritional status (iron focused) of older children with perinatal HIV infection and early onset ART and HIV uninfected children, with further comparisons in those with and without ID. Therefore, a fecal sample will be collected from all children before (Day 31) and after ID children received iron supplementation for 3 months (Day 121) to measure fecal calprotectin, which is a gut inflammation marker, and gut microbiota after iron supplementation.

The basic principle of the isotope dilution method is based on the initial enrichment shift (incorporation of isotopic tracers) followed by a sharp decrease in enrichment (corresponding to a fraction of the iron from senescent red blood cells (RBC) being transported to other tissues) and then the isotopic ratio reaches a stable slope after 7-8 months in toddlers [47] and after approximately 12 months in adults [48, 49]. Under these conditions, the rate of change of the isotopic signal ^{n}A (typically the tracer abundance in the body iron) can be expressed as:

$$\frac{d\left({}^{n}A\right)}{dt} = -k_{abs} \times {}^{n}A$$

where $d(^{n}A)/dt$ represents the rate of change of the isotopic signal ^{n}A per unit of time, and k_{abs} the rate of change constant. The solution of the equation is:

$${}^{n}A\left(t\right) = {}^{n}A_{0} \times e^{-k_{abs} \times t}$$

The half-life of the isotopic signal ^{n}A can be expressed as:

$$t_{1/2} = \frac{\ln\left(2\right)}{k_{abs}}$$

While k_{abs} can be seen as the turnover rate, which, in a logarithmic scale (natural log) scale, is equivalent to the slope of the isotopic signal decrease over time as in the following formula

$$\ln\left({}^{n}A_{t}\right) = -k_{abs} \times t + \ln\left({}^{n}A_{0}\right)$$

Biologically, the change in tracer abundance ^{n}A is proportional to the amount of iron of natural isotopic composition that is absorbed (called *tracee*), which progressively dilutes the proportion to the amounts of iron absorbed with natural isotopic composition. Therefore, the amount of iron absorbed from the diet as a whole will be directly proportional to the shift in isotopic ratio. Iron losses (sweat, desquamation, gastrointestinal tract (GIT) losses and others) will not affect the isotopic ratio per se: each loss in body iron will be reflected in a proportionally similar loss in tracer, leaving the ratio of tracer to body iron unchanged. Results from our pilot study in Malawi, assessing the body iron dilution of a stable isotopic label (^{57}Fe) given orally in 2012 to five pre-school age children, indicate that after one year of isotopic administration, the shift in isotopic ratio compared to the natural abundance is well above the detection limit of the mass spectrometric determination of isotopic ratios, allowing monitoring tracer dilution over the next several years.

As discussed before, after an iron isotopic tracer has equilibrated with the total iron body pool (steady state), the fractional disappearance of the circulating tracer over time is equivalent to the fractional iron absorption (FIA) over the time unit. A decrease (dilution) of the isotopic *tracer* ^{n}A abundance can occur

only through incorporation of *tracee*. The decrease in *tracer* abundance is therefore proportional to the incorporation of *tracee*, which can only happen through absorption from dietary and fortificant/supplemental iron (i.e. iron from LNS, or from iron supplements). The linear regression of ln(^{n}A) against time will be calculated for each participant on a 3-monthly basis in the turnover period. The slope of this regression line, k_{abs} reflects the absorption of iron per unit of time.

The tracer abundances in the body iron $^{n}A_{t}$ will be calculated from the measured isotopic ratios at times *t*:

$$^{n}A_{t} = \frac{^{56}a_{nat} - R_{56/n} \times {}^{n}a_{nat}}{R_{56/n}\left({}^{n}a_{T} - {}^{n}a_{nat}\right) + \left({}^{56}a_{nat} - {}^{56}a_{T}\right)}$$

Where $^{56}a_{nat}$ and $^{n}a_{nat}$ represent the abundances of isotopes ^{56}Fe and ^{57}Fe in the natural iron, $^{56}a_{T}$ and $^{n}a_{T}$ the abundances of ^{56}Fe and ^{57}Fe in the tracer, and $R_{56/n}$ the measured isotopic ratio ($^{56}Fe/^{57}Fe$).

The difference in the slopes will be equivalent to the average difference in iron absorption over time in each period and this measure will be independent from all anthropometric or iron status measurements in the participants. Since this methodology allows for a precise assessment of the rate of change in body iron, it follows that it will allow the quantification of the amount of iron absorbed on average over the observation period, through the relationship of:

$$Fe_{abs} = k_{abs} \times Fe_{tot}$$

Fe_{tot} is the mean total body iron between the time points. This can be calculated as follows [47]:

$$Fe_{tot} = Fe_{circ} + Fe_{nca} + Fe_{stor}$$

Where Fe_{circ} is the circulating body iron, Fe_{nca} is non-circulating, active iron and Fe_{stor} corresponds to storage iron. Circulating body iron Fe_{circ} will be calculated as follows:

$$Fe_{circ}\left[mg\right] = V_{blood}\left[L\right] \cdot Hb\left[g/L\right] \cdot 3.47\left[mg/g\right]$$

Blood volume (V_{blood}) will be estimated from age- and sex-specific formulas based on height and weight [54, 55]. It is anticipated that in the population under study, circulating iron accounts for the highest and most prominent share of Fe_{tot}, and will be used as a proxy for total body iron. The other compartments will be estimated as follows: Fe_{stor} can be estimated with a linear extrapolation using PF in the absence of inflammation as a proxy for iron stores [56], and Fe_{nca} has been established by other authors and has a typical value of 6 mg/kg [47].

During study 2, we will do bi-weekly morbidity monitoring for ID children during iron supplementation and monthly monitoring for IS children not receiving supplementation using phone calls. The following variables will be recorded during the studies and interrogated during analysis as potential confounders: 1) Tanner pubertal stage (specifically current menstrual pattern if post-menarcheal); 2) family home circumstances and socioeconomic metrics; 3) antibiotic exposures, tuberculosis treatment, antiretroviral therapy (specifically zidovudine and lamivudine exposure); and 4) hospital admissions (nosocomial pathogen exposure).

Participants becoming severely anemic (Hb < 80 g/L) at any time during the studies (Study 1 and 2) will receive standard of care treatment according to local guidelines, but will not be excluded from the study.

Participants developing secondary infections at any time during the study will receive standard of care treatment, and iron may be withheld for several days until the appropriate treatment is complete, but the participants will not be excluded.

Primary outcomes

The primary outcome in study 1 will be FIA (%) from the three different types of iron vehicles; and in study 2 iron turnover over 6 months, measured as K_{abs}, the slope of ^{57}Fe isotopic dilution from the end of the baseline enrichment equilibration period, which will define the daily iron requirement of both groups of children.

Secondary outcomes

Secondary outcomes will be: 1) iron status (FBC, PF, sTfR, PFe, Tsat, TIBC, EPO); 2) systemic and gut inflammation status (measured by hsCRP, AGP, Phep, IL-6, IFABP-1, IFABP-2, LBP, and fecal calprotectin); 3) gut microbiome; and 4) nutritional dietary status (zinc, vitamin A; intake of animal protein, iron, vitamin A, vitamin C, calcium, zinc and fiber).

Laboratory analysis and sample collection

Iron status and inflammation indices

FBC and PF (only for screening) will be measured right after blood withdrawal, using a hematology analyzer (Beckman Coulter LH750). Quality of Hb analysis will be ensured by regular measurement of controls provided by the manufacturer. The local study site coordinator will be responsible for the Hb measurements and further processing of whole blood samples at the study site. Blood withdrawal will be done by pediatric technicians or nurses with extensive experience with venipunctures with (trace element free) lithium-heparin vacutainers. Anemia for children 8-11 years old will be defined as Hb <11.5 g/dL, and for children 12 years old as Hb <12 g/dL [57]. After Hb measurement, whole blood aliquots of 2 mL will be frozen at the investigators facilities in Cape Town and will then be shipped to ETH Zurich, Switzerland, for the determination of the iron isotopic composition in blood. The remaining blood will be centrifuged and the plasma aliquoted and stored at −20°C at the study site until they will be shipped frozen to ETH Zurich, Switzerland, for analysis of iron status (PF, sTfR) and inflammation (AGP, hsCRP) using a multiplex immunoassay technique [58]. Phep, IFABP-2, LBP, IL-6 and EPO analyses using commercially available ELISAs. sCD14 will be measured by commercially available rapid test kits. Tsat is the percent of transferrin that has iron bound to it. It is the ratio PFe and TIBC, multiplied by 100. For PFe measurement, acid extraction of plasma with hydrochloric acid and trichloroacetic acid liberates the iron from transferrin and precipitates the protein. The acid reagent also contains thioglycolic acid, which reduces the iron to its ferrous oxidation state. Iron concentration is then determined by measuring the absorbance of the lilac color development. TIBC is a measure of iron-binding sites available on plasma transferrin. A solution of ferric chloride is added in sufficient quantity to the plasma sample to saturate the iron binding capacity of plasma. The excess unbound iron is removed and the iron concentration in the supernatant is measured colorimetrically. Iron concentration is determined by measuring the absorbance of the lilac color development [59].

To ensure quality of stored samples, the freezer at the study site is connected to a back-up power generator and a temperature log is completed daily by a laboratory technician. All samples (plasma, whole blood and stool) will be shipped with World Courier using temperature-controlled transport to ensure that the samples

remain frozen during shipment. During transport, World Courier will regularly control whether there is sufficient dry ice in the transport box and refill if necessary. Upon arrival in Switzerland, the samples will immediately be stored at −20°C at the ETH Zurich, Switzerland. The principal investigator (PI) and co-investigators located at ETH Zurich will be responsible for proper sample storage and further analysis of iron status and inflammation indices. ID will be defined as PF <30 μg/L [57] and/or elevated sTfR >8.3 mg/L [58], and IDA as Hb <11.5 or 12 g/dL and PF <30 μg/L [57] and/or sTfR >8.3 mg/L [58]. Expected CRP and AGP concentrations for healthy children are >5 mg/L and >1 g/L, respectively [58].

Only results of Hb from FBC (at screening also PF) can be provided immediately within the day of blood sampling. Results of analysis done in Switzerland due to the need of specific technologies not available in South Africa will be promptly shared with the local study team and will be later available on the public domain literature to inform supplementation practices in South Africa, Africa and worldwide.

The ratio of the iron forms in red blood cells

Two mL of whole blood will be frozen at −20°C immediately after blood was drawn at the study site and then transported frozen to ETH Zurich, Switzerland, in one batch at the end of the study for the measurement of the ratio of the forms (^{58}Fe, ^{56}Fe, ^{57}Fe) in the in the RBCs at ETH Zurich, Switzerland. All isotopically enriched blood samples will be mineralized in duplicate using an HNO_3/H_2O_2 mixture and microwave digestion followed by separation of the sample iron matrix by anion-exchange chromatography and a subsequent precipitation step with ammonium hydroxide [60]. All isotopic analysis will be performed by inductively coupled plasma mass spectrometry (ICP-MS) using a high-resolution magnetic sector mass spectrometer (Neptune, Thermo-Finnigan, Germany) equipped with a multi-collector system for simultaneous ion beam detection [61]. The PI and co-investigators in Switzerland will be responsible for this laboratory analysis.

Calculation of iron absorption

The amounts of ^{58}Fe, ^{56}Fe and ^{57}Fe in the RBCs will be calculated based on the shift in iron ratios and the estimated amount of iron circulating in the body. The circulating iron will be calculated based on the blood volume estimated from height and weight and measured Hb concentration [55]. The calculations will be based on the methods described by Turnlund et al. [62]. For calculation of FIA, 90% incorporation of the absorbed iron into RBCs will be assumed [63].

Stool collection and analysis

Fecal samples will be collected at study baseline, at equilibration period baseline (begin study 2) and after 3 months of supplementation during equilibration. The research team and field workers will carefully instruct the participating children and caregivers how to collect the children´s fecal samples. The children and caregivers will be provided with standard plastic containers, wooden spatulas, Anaerocult sachets (VWR International GmbH, Dietikon, Switzerland) to generate an anaerobic environment, along with an illustrated pamphlet to reinforce the sampling instructions. The fecal samples will be collected from the children in the morning at home, kept anaerobic in the sealed plastic containers and then brought to the visits at equilibration period baseline (day 31) and after 3 months of supplementation during equilibration (day 121). Stool aliquots will be frozen at −20°C on the day of collection. The local study coordinator will be responsible for quality assurance of sample collection and storage at the study site. Samples will then be

transported frozen (shipment on dry ice using World Courier service) to Switzerland for analysis. Upon arrival at ETH Zurich, Switzerland, samples will be stored at −20°C until further analysis. The PI and co-investigators in Switzerland will be responsible for proper sample storage and further stool sample management and analysis.

Fecal calprotectin, a gut inflammation marker, will be measured using the Calprest ELISA immunoassay for stool, following the manufacturer's procedures (Eurospital, Trieste, Italy). To ensure the quality of the measurements, controls provided by the manufacturer are analyzed together with the samples, standard curves are regularly performed in each run and all samples are measured in duplicate.
The gut microbiome analysis will be performed at the microbiology laboratory at Ku Leuven, Belgium. 16srDNA community profiling will be used to determine the microbial distribution with further quantitative PCR (qPCR) assessment of bacterial loads, microbiota phylogenetic profiling and quantitative microbiome profiling through parallelization of amplicon sequencing and flow cytometric enumeration of microbial cells [64].

Quality assurance and control

Quality assurance laboratory tests

All laboratory analyses will be done using controls/standards with known values and will be conducted by experienced personnel. The FBC measurement will be ensured using certified controls provided by the manufacturer of the Hematology Analyzer. The FBC measurement will be done by well-trained personnel for this measurement.
The measurement of PF, sTfR, CRP and AGP will be done by a technician who has developed the sophisticated method measuring all four parameters in a single aliquot of 100-200 µL [58]. To ensure the quality of these measurements certified quality control samples from the CDC/Atlanta as well as Biorad Liquicheck controls are analyzed together with the samples.
The measurement of the ratio of the four irons with different weights in the red blood cells will be controlled by using a special type of in-house controls, called spikes, which have a known ratio of the four iron forms. This allows quality assurance of the mass spectrometry used to measure the ratio in the blood samples. Technicians from ETH Zurich with extensive experience from numerous previous iron absorption studies will conduct the analysis.
The quality of the iron status, iron and gut inflammation analysis is ensured by performing standard curves routinely in each run; and external controls, provided by the manufacturer and are analyzed together with the samples and all samples are analyzed in duplicate. Analyses will be done by technicians from the ETH Zurich with extensive experience under close supervision of the PI and the Swiss co-investigators.

Data handling and record keeping / archiving

Data will be directly entered in the electronic case report form (eCRF, program Redcap) of each participant. The adverse events (AEs) including severe adverse events (SAEs) will be entered in the eCRFs. All SAEs must be reported immediately and within a maximum of 24 hours to the PI of the study. The investigator will re-evaluate the SAE and return the form to the site. SAEs resulting in death are reported to the local Ethics Committee (via local investigator) within 7 days.
Subjects will be given a unique identity number, the key to which will be kept in a protected data file. During data analysis, data will be connected only to ID numbers without personal information. Data will not be

forwarded to third parties and will only remain the property of ETH Zurich, Switzerland. Data on paper source files will be stored at ETH Zurich for a minimum of five years and a maximum of ten years after completion of the study. If publications result from the study, all data will be presented anonymously. The PI will notify the accredited Ethical Review Committee of the end of the study within a period of 8 weeks. The end of the study is defined as the last patient's last visit. In case the study is ended prematurely, the PI will notify the accredited Ethical Review Committee, including reasons for the premature termination. The investigator will report to the Ethical Review Committee according to the regulations. After completion of the trial, results will be presented to the caregivers of the participating children and as well to the community in village meetings. Results of the study will be published in a scientific journal and will be presented and discussed at conferences. The trial will be registered on clinicaltrial.gov.

Case Report Forms (CRF)

Data will be recorded using electronic CRFs. For each enrolled study participant, an eCRF is maintained. eCRFs will be kept current to reflect subjects' status at each phase during the course of the study. There will be no identifier on the eCRF (name or initials), but the study ID will be used.

Trained pediatric study nurses and the local study coordinator will fill in the eCRF. The local study site coordinator will review all data for completeness and accuracy. The co-investigator located both in Zurich and South Africa will regularly review all source data. Source data will be entered into a database using double entry.

Specification of source documents

The eCRF, including informed consent and the AE forms are considered as source data.

Record keeping / archiving

All study data will be archived for a minimum of 10 years after the termination of the study.

Data management

Data management system

Data will be double entered and a final data set will be created and locked. Copies of this final dataset will be stored at the study site (study site coordinator) and at ETH Zurich, Switzerland (PI).

Data security, access and back-up

The final dataset will be accessible to all investigators named in this application. Back-ups of this final dataset will be stored at the study site (study site coordinator) and at ETH Zurich, Switzerland (PI).

Analysis and archiving

Study data will be archived in our facilities in Cape Town (original study forms and electronic scanned copies). An electronic dataset will also be archived at ETH Zurich, Switzerland.

Electronic and central data validation

The data will be validated using range checks retrieved from previous studies in similar population groups.

Storage of biological material and related health data

Blood and fecal samples will be stored and kept at the HNL (ETH Zurich, Institute of Food, Nutrition and Health, Switzerland) until publication of results. The samples will be labelled with only a code. Access rights to this code are only granted to the PI (Prof. Michael Zimmermann) and co-investigators in Switzerland (Dr. Colin Cercamondi and Nadja Mikulic) from the HNL, Institute of Food, Nutrition and Health, ETH Zurich, Switzerland. Upon publication, the samples will be destroyed.

Publication and Dissemination policy

The results of Hb will be shared on the day of blood collection with the health provider and the participating families. The trial results will be summarized in a clear and understandable way to communicate them to the community (community meetings) and participating families.
During the final phase of the project, the ETH Zurich and Stellenbosch University Faculty of Medicine and Health Sciences, Cape Town, South Africa, will work together in the interpretation and dissemination of the findings of the study. Dr. Shaun Barnabas, a pediatric infectious diseases specialist, is the chief co-investigator for the South African team.
After statistical analysis of the study, the PI will make every endeavor to publish the data in a scientific journal.

Confidentiality, Data Protection

The PI and co-investigators are liable to treat the entire information related to the studies and the compiled data strictly confidentially. All assessed personal data will be analyzed in encrypted form and only used for scientific purpose. Data generation, transmission, archiving and analysis of personal data within these studies, strictly follows good clinical practice (GCP) regulation as well as the regulation on professional secrecy in clinical research. Prerequisite is the voluntary approval of the participant given by signing the informed consent prior start of participation of the study. Individual participant medical information obtained as a result of these studies is considered confidential and disclosure to third parties is prohibited. Participant confidentiality will be further ensured by utilizing participant identification code numbers to correspond to treatment data in the computer files. Such medical information may be given to the participant's personal physician or to other appropriate medical personnel responsible for the participant's welfare, if the patient has given his/her written consent to do so.

<u>Statistics</u>

Sample size calculations and statistical analysis

The sample size required for the single meal/supplement studies (Study 1) is based on previous iron isotope studies from the ETH Zurich. Assuming 80% power to detect a 30% difference in iron absorption between the two groups of children, a standard deviation (SD) of the log-transformed previous data of 0.228, and a type I error rate of 5%, we estimate we will need to include at least 40 iron deficient children in each group (HIV infection vs non-infection). For Study 2, sample size calculations indicate that with a pooled log SD of 0.01 and an expected difference in body iron losses of 0.015 mg/kg body weight/d [49], 22 subjects are needed for inter-subject comparison with a type I error rate of 5% and 90% power.

However, we will enroll 90 **iron deficient** (HIV infected (n=45) and uninfected (n=45)) and 90 **iron sufficient** (HIV infected (n=45 and uninfected (n=45)) children at the beginning of Study 1, because: a) the

isotope dilution method has not been applied before in this age group and variability in Studies 1 and 2 may be somewhat higher than in previous studies; and b) we anticipate there will be a 10-15% drop-out rate over the 2-year studies. The 180 children, will participate in the single meal absorption study (Study 1), based on the sample size calculation of 40 per group (above). The **iron deficient** children will get three different meals with a single iron dose each; the remaining 90 children from the **iron sufficient** group will receive the 12 mg ^{57}Fe as $FeSO_4$ given in fruit juice. All 180 children will enter Study 2.

Meal administration on days 1 and 3 will be randomized using a computer generated, stratified block randomization procedure. Data will be analyzed using R statistical programming environment (R software), double entered and their distribution checked for normality. Not normally distributed data will be log transformed for analysis. Normally distributed data will be expressed as means and SD. For log-transformed data, we will obtain geometric means (GM) and corresponding SD for absolute concentrations by taking the antilog of these values. To explore associations between FIA, iron utilization (isotopes), iron status and inflammation, we will fit linear regression models including FIA, systemic iron utilization, PHep, AGP, hsCRP, PF, sTfR, Tsat, EPO, LBP, IFABP-2, IL-6 and fecal calprotectin, followed by post hoc Bonferroni test for multiple comparisons. We will calculate fractional erythrocyte incorporation of oral labels [60]. To compare between the two groups, HIV infected and uninfected children, in both studies, we will use student t-tests for independent groups. Iron status will be assessed with univariate general linear models (GLM) using baseline variables as covariates. For all statistical analyses, p-values <0.05 will be considered as significant.

d. Timeframe and study schedule

Project activity	Year 1				Year 2			
Ethical approvals	■							
Assessment, recruitment		■						
Study 1		■	■	■				
Data analysis Study 1					■			
Study 2 Equilibration period				■	■	■	■	
Study 2 Turnover period							■	■
Study 2 Data analysis								■

	Baseline	Study period								Endpoint
**Visit**	**1**	**2**	**3**	**4**	**5**	**6**	**7**	**8**	**9**	**10**
**Day**	**-1**	**1**	**3**	**17**	**31**	**121**	**151**	**271**	**361**	**451**
Informed consent	x									
In/exclusion criteria	x									
Randomization	x									
Anthropometrics	x			x*	x	x	x	x	x	x
Blood sampling	x			x*	x	x	x	x	x	x
Maize porridge test meal*		x								
LNS/fruit juice test meal			x							
Iron tablet*				x	x					
Adverse events/morbidity	x	x*	x	x*	x	x*	x	x	x	x
Faecal sampling*					x	x				
Food frequency questionnaire (subgroup: ½ of cohort)			x		x					
Primary outcome (FIA (%), k_{abs})				x*	x		x	x	x	x
Secondary outcome:<br>- in blood samples[a]<br>- in stool samples[b]	x			x*	x<br>x*	x*	x	x	x	x

*If child is iron-deficient

[a]Iron status: FBC, PF, sTfR, PFe, Tsat, TIBC, EPO; inflammation status: hsCRP, AGP, PHep, IL-6, IFABP-1, IFABP-2, LBP

[b]Fecal calprotectin, gut microbiome measurements

e. Questionnaires/surveys if required

DD MM YY DD
MM YY

Participant Number: ☐☐☐☐☐☐ **Birth Date:** ☐☐☐☐☐☐ **Interview Date:** ☐☐☐☐☐☐

Interviewer: ___________________________

QUANTITATIVE FOOD FREQUENCY QUESTIONNAIRE

Greeting

Thank you for giving up your time to participate in this survey. We would like to find out what you / your child usually eat and drink. This information is important to know as it will tell us if children are eating enough, of the right foods, and if they are healthy.

Please think carefully about the food and drinks the child, that has been identified as a participant in this study survey, have consumed during the past 6 months. I will now go through a list of foods and drinks with you and I would like you to tell me:

- if the child eats these particular foods,
- how the food is prepared (by you or the child's caretaker),
- how much of the food the child eat at a time, and
- how many times a day the child eats it and if he or she does not eat it every day, how many times a week or a month it is eaten?

To help you to describe the amount of a food, I will show you models of different amounts of the food. Please say which model is the closest to the amount eaten, or if it is smaller, between sizes or bigger than the models. Amounts must be reported as cups (c), tablespoons (T), serving spoons (SP) or teaspoons (t).

- **THERE ARE NO RIGHT OR WRONG ANSWERS.**
- **EVERYTHING YOU TELL ME IS CONFIDENTIAL.**
- **IS THERE ANYTHING YOU WANT TO ASK NOW?**
- **ARE YOU WILLING TO GO ON WITH THE QUESTIONS?**

COPYRIGHT RESERVED: NATIONAL FOOD CONSUMPTION SURVEY 1999.

INSTRUCTIONS TO FIELDWORKERS:

CIRCLE THE CHOSEN ANSWER AND FILL IN THE AMOUNT AND TIMES EATEN IN THE APPROPRIATE COLUMNS.

I will ask you about the type and the amount of food the child has been eating during the last 6 months. Please tell me if the child eats the food, how much the child eats and how often the child eats it.

	FOOD	DESCRIPTION	CODE	QUANTITY (g/ml)	AMOUNT USUALLY EATEN (HHM)	AMOUNT USUALLY EATEN (g)	P/D	D/W	P/M	SEL/NEV
PORRIDGE	**Maize-meal Porridge**	Stiff (Pap) –Plain	**3400**	1c stiff = 250 g<br>1T = 75g						
		- Enriched	**4278**							
		Soft (Slappap) – Plain	**3399**	1c soft = 250g<br>1T = 75g						
		- Enriched	**4277**							
		Crumbly (Phutu) – Plain	**3401**	1 c crumbly = 140 g						
		- Enriched	**4279**	1T = 30g						
	**Sour Porridge**	Maize with Vinegar<br>Maize Fermented<br>Mabella with Vinegar<br>Mabella Fermented	**P0001**<br>**P0002**<br>**P0003**<br>**P0004**	½c = 125g<br>1c = 250g						
	**Mabella Porridge/Cornrice**	Stiff	**3437**	½ c = 125g						
		Soft	**3437**							
	**Maltabella Porridge**	Stiff	**3241**	½ c = 125g						
		Soft	**3241**							
	**Oats Porridge**	Brand Name:	**3239**	2c = 125g						
	**Other Cooked Cereals**	Specify Type:								
	**Milk on Porridge**<br>**(Circle type usually used)**	None								
		Whole/Fresh	**2718**	little = 30g<br>med = 60g<br>much = 125g						
		Sour	**2787**							
		2%	**2772**							
		Fat Free / Skim	**2775**							
		Milk Blend	**2771**							
		Soy Milk	**2737**							
		Condensed (Whole, Sweet)	**2714**	1t = 10g						
		Condensed (Skim, Sweet)	**2744**							
		Evaporated Whole	**2715**	1t = 3g						
		Evaporated Low Fat	**2827**							
		Non-Dairy Creamer	**2751**	1t = 4g						

	FOOD	DESCRIPTION	CODE	QUANTITY (g/ml)	AMOUNT USUALLY EATEN (HHM)	AMOUNT USUALLY EATEN (g)	P/D	D/W	P/M	SEL/NEV
PORRIDGE	**Is sugar added to porridge?** **(Circle type usually used)**	None								
		White	**3989**	1t sugar = 6g						
		Brown	**4005**							
		Syrup	**3988**	1t honey/syrup = 15g						
		Honey	**3984**							
		Sweetener: Type	**P0016**							
	**Is fat added to porridge?** **(Circle type usually used)**	None								
		Animal Fat (Butter)	**3479**	1t marg/oil = 5g						
		Hard Margarine	**3484**							
		Soft Margarine (PM)	**3496**							
		Soft Margarine (Med)	**3531**							
		Sunflower Oil	**3507**							
		Peanut Butter	**3485**	1t = 12g						
BREAKFAST CEREALS	**Breakfast Cereals**	Specify types usually eaten		**(See Manual)**						
	**Baby/Infant Cereals** **(Circle Type)**	Mixed Dry (Nestum 2)	**2834**	1t = 2g<br>1T = 8g<br>½ c = 20g						
		Mixed Dry (Purity)	**2842**							
		Wholewheat Dry (Purity)	**2861**							
		Rice and Maize Dry (Nestum)	**2835**							
		Rice Dry (Purity)	**2862**							
		Wheat Dry (Nestum 1)	**2832**							
		With Milk Dry (Cerelac)	**2836**							
		Junior Cereal Dry	**2833**							
		Other								
	**Milk on Cereal**	Specify Type		**(See Manual)**						
	**Is sugar added to cereal?**	Specify Type		**(See Manual)**						
	**Is fat added to cereal?**	Specify Type		**(See Manual)**						

How many times a week does the child eat porridge or breakfast cereals at any time of day (not only breakfast): ______

I am now going to ask about starchy foods:

	FOOD	DESCRIPTION	CODE	QUANTITY (g/ml)	AMOUNT USUALLY EATEN (HHM)	AMOUNT USUALLY EATEN (g)	P/D	D/W	P/M	SEL/NEV
STARCHES	**Samp/Maize Rice**	Samp, White	**3250**	1T = 55g; 1 SP = 125g; ½ c = 125g						
		Maize Rice	**3250**							
		Sweetcorn Boiled	**3725**	1T = 25g; 1 SP = 45g; ½ c 65g						
	**Samp and Beans**	Specify Ratio:	**3402**	1T = 50g 1SP = 125g ½ c = 125g						
	**Samp and Peanuts**	Specify Ratio:	**P0013**							
	**Rice: Specify Brands Names**	White	**3247**	1T = 25g; 1SP = 60g; ½ c = 65g						
		Brown	**3315**							
STARCHES	Stamped Wheat		**3249**	1T = 30g; 1SP = 80g; ½ c=80g						
	Pastas	Macaroni	**3262**	1T = 35g; 1SP = 70g; ½ c = 90g						
		Spaghetti Plain	**3262**							
		Spaghetti and Tomato Sauce	**3258**	1T =45g; 1SP =80g; ½ c=125g						
		Other: Specify								
	**Do you add fat to any of these starchy foods?**	Yes ____ No ____ If yes, specify types, amounts and to which food?		**(See Manual)**						
	**How many times a week does the child eat the above starchy foods? ______________**									
BREADS AND SPREADS	**Now we come to bread and bread spreads:**									
	**Bread/Bread Rolls**	White	**3210**	Wh+Br 10mm = 30g Wh + Br 20mm = 60g Wh + Br 30mm = 100g ½ loaf = 400g						
		Brown	**3211**							
		Whole Wheat	**3212**	Ww 10mm = 35g						
	**Other Breads (Specify Types)**	Raisin	**3214**	m/s = 30g; L/s = 50g						
		Maize Meal	**3278**							
		Sweetcorn	**3379**							
		Rye	**3213**							
		Pumpernickel	**3283**							
		Other								
	**How many times per week does the child eat bread? _________**									
	**FOOD**	**DESCRIPTION**	**CODE**	**QUANTITY (g/ml)**	**AMOUNT USUALLY EATEN (HHM)**	**AMOUNT USUALLY EATEN (g)**	**P/D**	**D/W**	**P/M**	**SEL/NEV**

	FOOD	DESCRIPTION	CODE	QUANTITY (g/ml)	AMOUNT USUALLY EATEN (HHM)	AMOUNT USUALLY EATEN (g)	P/D	D/W	P/M	SEL/NEV
BREADS AND SPREADS	**Dumpling**	(Depends on specific areas)		**(See Manual)**						
	**Vetkoek**	(Depends on specific areas)		8 cm diam = 60g						
	**Provita**		**3235**	6g						
	**Crackers**	Cream Crackers	**3230**	8g						
		Refined (eg. Tuc)	**3331**	4g						
		Wholewheat	**3391**	8g						
	**Pizza**	(Specify Toppings)		**(See Manual)**						
	**Hot Dogs**	(Specify Sausage)		**(See Manual)**						
	**Hamburgers**	(Specify Meat)		**(See Manual)**						
	**Are any of the following spreads on the child's bread?** **Fat Spreads: (Tick box)**	Butter	**3479**	1t = 5g						
		Butro	**3523**							
		Animal Fat (Beef Tallow)	**3494**							
		Lard	**3495**							
		Hard Margarine	**3484**							
		Soft Margarine (PM)	**3496**							
		Soft Margarine (Med)	**3531**							
	**PeanutButter**		**3485**	1t = 12g						
	**Sweet Spreads**	Jam	**3985**	1t = 15g						
		Syrup	**3988**							
		Honey	**3984**							
	**Marmite/OXO**	Marmite	**4030**	thin = 2g; med = 4g; thick=7g						
		Oxo	**4029**							
	**Paste**	Fish Paste	**3109**	thin = 5g; med = 7g;						
		Meat Paste	**2917**	thick = 10g						
	**Cheese (Specify Types)**	Cheddar	**2722**	grated: med = 10g; thick = 15g cubes = 30g; slice = 8g; cheezi = 20g						
		Gouda	**2723**							
		Cottage Low-Fat Cheese	**2760**	med = 20g; thick = 30g						
		Cream Cheese	**2725**	thin = 10g; med = 20g						
		Other								
	**Cheese Spreads (Specify Types)**		**2730**	med = 12g; thick = 25g						
	**Atchar**		**3117**	1T = 14g; 1SP = 60g						

	**Other Spreads (Specify Types)**									

You are being very helpful. Can I ask you about protein foods? These are: meat, beans, chicken, fish and eggs.

**CHICKEN**		Boiled with skin	**2926**	Breast + skin = 125g<br>Thigh =80g<br>Drumstick = 42g<br>Foot = 30g<br>Wing = 30g						
		Boiled without skin	**2963**							
		Fried in batter/crumbs	**3018**							
		Fried – not coated	**2925**							
		Roasted/grilled with skin	**2925**							
		Roasted/grilled without skin	**2950**							
	**Chicken Bones Stew**	(Specify ingredients)	**P0048**							
	**Chicken Heads**		**2999**							
	**Chicken Stew**	With Vegetables	**3005**	1SP = 90g;<br>½ c = 125g						
		With Tomato & Onion	**2985**							
	**Chicken Feet**		**2997**	Foot = 30g						
	**Chicken Offal**	Giblets	**2998**	stomach = 20g						
	**Chicken Liver**		**2970**	Liver = 30g						
	**Chicken Pie**	Commercial or homemade	**2954**	med = 150g						
**RED MEAT**	**Beef**	Roasted with Fat	**2944**	120 x 60 x 5 = 35g<br>120 x 60 x 10 = 70g						
		Roasted, Fat Trimmed	**2960**							
		Rump, Fried with Fat	**2908**	S/s 130 x 70 x 15 = 125g<br>L/s 165 x 70 x 30 = 270g						
		Rump, Fried, Fat Trimmed	**2959**							
		Stewed/Boiled With Fat (Cabbage)	**3006**	1SP = 105g;<br>½ c = 125g						
		Stewed/Boiled Without Fat (Vegetables)	**2909**							
		Mince With Tomato and Onion	**2987**	1T=40g;1SP=85g;<br>½ c=100g						
		Other Preparation Methods:								
	**Mutton**	Fried/Grilled: With Fat	**2927**	Loin chop = 60g;<br>Rib chop = 40g						
		Fried/Grilled: Without Fat	**2934**							
		Stew: Plain	**2974**	1SP = 105g;<br>½ c = 125g						
		Stew: Irish (Vegetables)	**2916**							
		Stew: Curry	**3039**							
		Stew: Greenbean	**3040**							
		Other Preparation Methods:								

	FOOD	DESCRIPTION	CODE	QUANTITY (g/ml)	AMOUNT USUALLY EATEN (HHM)	AMOUNT USUALLY EATEN (g)	P/D	D/W	P/M	SEL/NEV
RE	**Pork**	Fried/Grilled: With Fat	**2930**	Chop: 115 x 80 x 20 =						

		Fried/Grilled: Without Fat	**2977**	100g<br>Schnitzel: 115 x 80 x20 = 110g<br>Roast: 110x 65 x 5 = 30g<br>1SP = 105g;<br>½ c = 125g						
		Roast With Fat	**2958**							
		Roast Without Fat	**2978**							
		Other Preparation Methods:								
	**Goat**	Fried/Grilled: With Fat	**P0008**	120 x 60 x 5 = 35g						
		Fried/Grilled: Fat Trimmed	**P0009**	120 x 60 x 10 = 70g						
		Stewed (Plain)	**4281**	1SP = 105g						
		Stewed (With Vegetables)	**4282**	½ c = 125g						
		Other Preparation Methods:								
	**Offal**	"Vetderm" Fried	**P0023**	1SP = 105g;<br>½ c = 125g						
		Liver: Beef (Fried)	**2920**	80g						
		Liver: Sheep (Fried)	**2955**	55g						
		Kidney (Beef)	**2923**	85g						
		Kidney (Sheep)	**2956**	30g						
		Tripe, Beef, Cooked in Milk	**2951**	1SP = 105g;<br>½ c = 125g						
		Heart (Beef)	**2968**	60g						
		Heart (Sheep)	**2969**	60g						
		Lung (Beef)	**3019**	60g						
**MEAT: GENERAL**	**Wors/Sausage**	Fried	**2931**	Thin x 200mm = 45g;<br>Thick x 165mm = 90g						
	**Bacon**	Fat	**2906**	1 rasher = 10g						
		Lean	**2915**							
	**Cold Meats**	Polony	**2919**	Slice 5mm thick = 8g<br>Comm slice = 16g						
		Ham	**2967**	Med slice = 25g						
		Viennas	**2936**	100mm = 30g;<br>150mm =40g						
		Other								
	**Canned Meats**	Bully Beef	**2940**	138 x 85 x 3 = 20g;<br>½ c = 100g						
		Other (Specify)								
	**Meat Pie**	Bought (Steak & Kidney)	**2957**	120g						
		Other (Specify)								

	FOOD	DESCRIPTION	CODE	QUANTITY (g/ml)	AMOUNT USUALLY EATEN (HHM)	AMOUNT USUALLY EATEN (g)	P/D	D/W	P/M	SEL/NEV
**MEAT: GENER**	**Legumes (Specify dried beans/peas/legumes)**	Stews (Bean, Potato & Onion)	**3178**	1T=60g; 1SP = 120g; ½c=125g						
		Soups: Commercial	**3165**	½ c = 125g						

		Split Pea	**3157**	1T=35g; 1SP = 80g; ½ c = 130g						
		Lentil	**3153**							
		Beef & Vegetables	**3159**							
		Bean	**3145**							
		Legume Salad	**3174**	1T=40g; 1SP=105g; ½ c=135g						
	**Soya Products e.g. Toppers / Imana**	(Specify)	**3196**	1SP = 85g; ½ c = 120g						
**FISH**	**Fried Fish (Fresh or Frozen, Fried in Sun Oil)**	With Batter/Crumbs	**3094**	Small 50 x 55 x 30 = 60g;						
		Without Batter/Crumbs	**3084**	Med 100 x 55 x 30 = 120g						
	**Canned Fish**	Pilchards in Brine	**3055**	1 Pilchard = 75g						
		Pilchards in Tomato Sauce	**3102**							
		Pilchards, Mashed	**3102**	1 SP = 85g; ½ c = 100g						
		Sardines in Oil	**3104**	Ss = 7g; L/s = 25g						
		Sardines in Tomato Sauce	**3087**							
		Tuna in Oil	**3093**	¼ c = 50g						
		Tuna in Brine	**3054**							
		Other (Specify)								
	**Pickled Fish/Curried Fish**		**3076**	1 SP = 95g; ½ c=140g						
	**Do you remove fish bones before eating canned fish? Yes ___No ___**									
	**Fish Cakes**	Fried: Oil/Butter/Margarine	**3098**	65 x 15mm = 50g						
	**Fish Fingers**	Fried: Oil/Butter/Margarine	**3081**	85mm = 35g						
**EGGS**	**Eggs**	Boiled/Poached	**2867**	1 egg = 50g						
		Scrambled in Oil	**2889**	1T = 35g; 1SP = 80g; ½c=115g (approx. 2 eggs)						
		In Butter	**2886**							
		In Margarine	**2887**							
		Fried in Oil	**2869**	1 egg = 52g						
		In Butter	**2868**							
		In Margarine	**2877**							
		In Bacon Fat	**2870**							
		Curried	**2902**	1 egg + sauce (1T) = 75g						

How many times a week does the child eat meat, beans, chicken, fish or eggs? ______________

**Are there any other foods in this category that the child eats? If yes, please list these foods/dishes**					YES		NO	
**FOOD**	**DESCRIPTION**	**CODE**	**AMOUNT USUALLY EATEN (HHM)**	**AMOUNT USUALLY EATEN (g)**	**TIMES EATEN**			
					**Per day**	**Days per week**	**Per month**	**Seldom/ Never**

We now come to vegetables:

	FOOD	DESCRIPTION	CODE	QUANTITY (g/ml)	AMOUNT USUALLY EATEN (HHM)	AMOUNT USUALLY EATEN (g)	P/D	D/W	P/M	SEL/NEV
VEGETABLES	**Cabbage**	Boiled, Nothing Added	**3756**	1T=30g; 1SP=55g; ½ c=80g						
		Boiled with Potato, Onion and Fat	**3813**	1T=35g; 1SP=75g; ½ c=80g						
		Fried, Nothing added	**3812**	1T=30g; 1SP=55g; ½ c=80g						
		Boiled, then fried with potato, onion	**3815**	1T=35g; 1SP=75g; ½ c=80g						
		Other								
	**Spinach/Marog/Imifino/ Amaranth Leaves Other Green Leafy Vegetables: List Names**	Boiled, nothing added	**3980**	1T=40g; 1SP=105g; ½ c=90g						
		Boiled, fat added	**3898**	1T=40g; 1SP=105g; ½ c=90g						
		Boiled with Onion, Potato and Fat	**3901**	1T=50g; 1SP=105g; ½ c=110g						
		Boiled with Peanuts	**P0015**	1T=55g; 1SP=120g; ½ c=105g						
		Other:								
	**Tomato and Onion "Gravy"/ Relish/Chow/Sheshebo**	Home Made with Sugar	**3910**	1T = 35g; 1SP = 75g; ½ c = 140g						
		Home Made, no Sugar	**3925**							
		Canned	**4192**							
	**Pumpkin (Specify Type)**	Boiled, nothing added	**4164**	1T = 45g; 1SP = 85g; ½ c = 105g						
		Cooked in Fat and Sugar	**3893**							
		Other								
	**Carrots**	Boiled, Sugar and Fat	**3818**	1T = 25g; 1SP=50g; ½ c = 85g						
		With Potato/Onion (HM)	**3822**	1T=35g; 1SP=70g; 1/2 c=105g						
		Raw, Salad (Sugar added)	**3721**	1T = 25g						
		Chakalaka	**P0046**							
		Other								
	**Mealies/Sweet Corn**	On Cob	**3725**	1T =30g; 1SP = 60g; ½ c =95g						
		Off Cob – Creamed, Sweet Corn	**3726**	1T = 55g; 1SP = 125g; ½ c = 135g						
		Off Cob – Whole Kernel Canned	**3942**							
		Other								
	**Beetroot**	Cooked (No Sugar)	**3698**	1T=40g; 1SP = 70g; ½ c = 80g						
		(With Sugar)	**3699**							
		Salad (Grated)	**3699**	1T = 25g; 1SP = 65g						

	FOOD	DESCRIPTION	CODE	QUANTITY (g/ml)	AMOUNT USUALLY EATEN (HHM)	AMOUNT USUALLY EATEN (g)	P/D	D/W	P/M	SEL/NEV
VEGETABLES	**Potatoes**	Boiled/Baked with Skin	**4155**	S/s = 60g; m/s = 90g						
		Without Skin	**3737**							
		Mashed (WM)	**3876**	1T=50g; 1SP = 115g; ½ c = 125g						
		Roasted	**3878**	1 med = 70g						
		French Fries/Potato Chips	**3740**	½ c = 50g; med = 80g						
		Salad	**3928**	1T = 45g; 1SP = 105g; ½ c = 120g						
		Other								
	**Sweet Potatoes**	Boiled/Baked with Skin	**3748**	1T = 50g; 1SP = 110g; ½ c = 145g						
		Without Skin	**3903**							
		Mashed (With Sugar)	**3749**							
		Other								
	**Green Beans**	Green, Frozen	**4123**	1T = 25g; 1SP=60g; 1/2 c=80g						
		Cooked, Potato & Onion (HM)	**3792**	1T = 40g; 1SP = 75g; ½ c =120g						
		Other								
	**Peas**	Green, Frozen, Boiled	**4146**	1T=30g; 1SP = 65g; ½ c = 85g						
		Green, Frozen with Sugar, Boiled	**3720**							
		With Sugar and Butter	**3859**							
	**Green Peppers**	Raw	**3733**							
		Cooked	**3775**							
	**Brinjal/Egg Plant**	Cooked	**3700**	1 slice = 20g (70 mm)						
		Fried in Oil	**3802**	+ batter = 30g						
		Stew (oil, onions, tomato)	**3798**	1T=50g; 1SP=100g;1/2c=130g						
	**Mushrooms**	Raw	**3842**	1T=30g; 1SP = 65g; 1/2c = 80g						
		Sauteed in brick margarine	**3839**							
		Sauteed in oil	**3841**							
	**Onions**	Sauteed in Sun Oil	**3730**	1T = 50g						
	**Salad Vegetables**	Raw Tomato	**3750**	Med = 120g; slice = 15g						
		Lettuce	**3723**	1 med leaf = 30g						
		Cucumber	**3718**	Med slice = 10g; thick = 15g						
		Avocados	**3656**	¼ avo (80 x 50mm) = 40g						

	FOOD	DESCRIPTION	CODE	QUANTITY (g/ml)	AMOUNT USUALLY EATEN (HHM)	AMOUNT USUALLY EATEN (g)	P/D	D/W	P/M	SEL/NEV
VEGETABLES	Other Vegetables: Specify									
	If you fry vegetables or add fat, specify type of fat usually used	Butter	3479	1t = 5g						
		Butro	3523							
		Animal Fat (Beef Tallow)	3494							
		Lard	3495							
		Hard Margarine (Brick)	3484							
		Soft Margarine (Tub, PM)	3496							
		Soft Margarine (Med)	3531							
	Vegetable Purees with or without meat for babies or infants:<br><br>(Specify)	First Food average Vegetable (Jar)	2851	1t = 5g<br>1T = 15g<br>½ c = 47g						
		Junior Food Veg (Jar)	2849							
		Junior Food Veg Plus Meat	2848							
		Infant Dinner, Beef and Veg	2841							
		Infant Dinner, Chicken and Veg	2840							
		Infant Dinner, Mixed Veg	2839							
		Other								
DRESSINGS	Mayonnaise/Salad Dressing	Mayonnaise – Bought	3488	1t = 10g<br>1T = 40g						
		- Home-made	3506							
		Cooked Salad Dressing	3503	1t = 5g; 1T = 15g						
		Salad Dressing, low-oil	3505							
		Salad Dressing, French	3487							
		Oil – Olive Oil	3509	1t = 5g; 1T = 15g						
		- Sunflower Oil	3507							
		- Canola	4280							

How many times a week does the child eat vegetables? _____________

How many times will this be fresh? __________ Canned ___________ Frozen ____________

I will now ask about fruit:

	FOOD	DESCRIPTION	CODE	QUANTITY (g/ml)	AMOUNT USUALLY EATEN (HHM)	AMOUNT USUALLY EATEN (g)	P/D	D/W	P/M	SEL/NEV
FRUIT	**Apples**	Fresh	**3532**	1T=60g; ½ c = 120g; 1 med = 150g (52 x 66)						
		Canned, Pie, Unsweetened	**4216**							
	**Bananas**		**3540**	1 med = 75g						
	**Oranges/Naartjies**		**3560**	Med (7cm) = 180g						
	**Grapes**		**3550**	Med bunch = 230g; ½ c = 90g						
	**Peaches**	Fresh	**3565**	1 med = 150g (60 x 65)						
		Canned in Syrup	**3567**							
	**Apricots**	Fresh	**3534**	1 med = 35g						
		Canned in Syrup	**3535**							
	**Mangoes**	Fresh	**3556**	135mm = 350g						
		Canned in Syrup	**3633**							
	**Pawpaw**		**3563**	Wedge 165 x 26 x 27 = 90g						
	**Pineapple**	Raw	**3581**	1 slice (85 x 10mm) = 40g						
		Canned in Syrup	**3648**							
	**Guavas**	Fresh	**3551**	Med (6cm) = 95g						
		Canned in Syrup	**3553**							
	**Pears**	Fresh	**3582**	1 med (80 x 65mm) = 165g						
		Canned in Syrup	**3583**							
	**Wild Fruit and Berries: (Specify Type)**									
	**Dried Fruit (Also as Snacks)**	Raisins	**4232**	1 handful = 27g						
		Prunes (Raw)	**4230**	1T = 50g; ½ c = 110g; 1 = 12g						
		Prunes (Cooked with Sugar)	**3564**							
		Peaches (Raw)	**3568**	1 med = 150g (60 x 65)						
		Peach (Cooked with Sugar)	**3569**							
		Apples (Raw)	**3600**	1T=60g; ½ c = 120g; 1 med = 150g (52 x 66)						
		Dried Fruit Sweets	**3995**	**(See Manual)**						
		Other								
	**Fruit Purees for Babies or Infants (Specify Types)**	First Food Average (Jar)	**2852**	Jar = 200g 1t = 11g ½ c = 125g						
		Junior Fruit (Jar)	**2863**							
		Strained Fruit (Jar)	**2854**							
		Infant Dinner, Guava and Custard	**2837**							

	FOOD	DESCRIPTION	CODE	QUANTITY (g/ml)	AMOUNT USUALLY EATEN (HHM)	AMOUNT USUALLY EATEN (g)	P/D	D/W	P/M	SEL/NEV
		Other								
	**Other Fruit**									
	**How many times a week does the child eat fruit? ____________** **How many times will this be fresh ____________ Canned ____________ Frozen ____________**									
DRINKS	**Tea**	Ceylon	**4038**	Teacup = 180ml; mug = 250ml						
		Rooibos	**4054**							
	**Sugar Per Cup of Tea**	Specify Type: White<br>Brown	**3989**<br>**4005**	1t sugar = 6g						
	**Milk per Cup of Tea**	Fresh/Long Life Whole	**2718**	20ml – tea in cup<br>35ml – tea in mug<br>40ml – coffee in cup<br>75ml – coffee in mug						
		Fresh/Long Life 2%	**2772**							
		Goat	**2738**							
		Fresh/Long Life from (skimmed)	**2775**							
		Whole Milk Powder Reconstituted (Specify Brand)	**2831**	1t = 4g						
		Skimmed Milk Powder, reconstituted (Specify Brand)	**2719**	1t = 4g						
		Milk Blend, reconstituted (Specify Brand)	**2771**	20ml – tea in cup<br>35ml – tea in mug<br>40ml – coffee in cup<br>75ml – coffee in mug						
		Whitener/non-dairy creamer (Specify Brand)	**2751**	1t = 4g						
		Condensed Milk (Whole)	**2714**	1t = 10g						
		Condensed Milk (Skim)	**2744**							
		Evaporated Milk (Whole)	**2715**	1t = 3g						
		Evaporated Milk (Low-Fat)	**2827**							
		None								
	**Coffee**		**4037**	Teacup = 180ml; mug= 250ml						
	**Sugar per Cup of Coffee**	Specify Type: White<br>Brown	**3989**<br>**4005**	1t sugar = 6g						

	FOOD	DESCRIPTION	CODE	QUANTITY (g/ml)	AMOUNT USUALLY EATEN (HHM)	AMOUNT USUALLY EATEN (g)	P/D	D/W	P/M	SEL/NEV
	**Milk per Cup of Coffee**	Specify Type		**(See Manual)**						
DRINKS	**Milk as such:**<br>**What type of milk does the child drink as such?**	Fresh/Long Life/ Whole	**2718**	To drink ½ c = 125ml<br>Baby bottle = 250ml						
		Fresh/Long Life/2%	**2772**							
		Fresh/Long Life/Fat Free (skimmed)	**2775**							
		Goat	**2738**							
		Sour/Maas	**2787**							
		Brand:<br>Infant Formulas (Specify)								
	**Milk drinks. Specify Brands, including milk supplements and type of milk used**	Nestle Drinking Chocolate	**4287**	1t = 5g						
		Malted Milk Beverage, no Sugar (eg Milo)	**2735**	1t = 5g						
		Flavoured Milk:	**2774**	Carton = 250ml;<br>S/s plastic = 350 ml						
		Other								
	**Yoghurt**	Drinking Yoghurt	**2756**	S/s = 175ml<br>Yogisip = 350ml<br>½ c = 125g						
		Thick Yoghurt: Plain, Fat-Free	**2778**							
		WM Plain	**2757**							
		- Fruit, Low Fat	**2732**							
		Other								
	**Squash**	Sweeto, Sixo	**3982**	Small glass = 150ml<br>Medium glass = 250 ml<br>Large glass = 500 ml<br>S/s bottle = 350ml<br>L/s bottle = 500ml<br>S/s can = 350ml						
		Oros/Lecol with Sugar	**3982**							
		Artificial Sweetener	**3990**							
		Kool Aid	**3982**							
		Other								
	**Fruit Juice**	Fresh/Liquifruit/Ceres/Purity	**2866**	1 Liquifruit s/s = 250ml<br>1 Liquifruit L/s = 500 ml<br>S/s bottle = 350ml<br>L/s bottle = 500ml<br>S/s can = 350ml						
		"Tropica"/mixture with milk	**2791**							
	**Fruit Syrups**	Average	**2865**	1t = 5g						
		Guava Syrup	**2864**							
	**Fizzy Drinks (e.g. Coke, Fanta)**	Sweetened	**3981**	S/s bottle = 350ml<br>L/s bottle = 500ml<br>S/s can = 340ml						
		Diet	**3990**							

	FOOD	DESCRIPTION	CODE	QUANTITY (g/ml)	AMOUNT USUALLY EATEN (HHM)	AMOUNT USUALLY EATEN (g)	P/D	D/W	P/M	SEL/NEV
DRINKS	**Magou/Motogo**		**4056**	1 carton = 500 ml						
	**Alcoholic Beverages such as Sorghum Beer**	Specify: Sorghum Beer	**4039**	(See Manual)						
	**Other (Please Specify)**									
SNACKS	**Please indicate what types and amounts of snacks, puddings and sweets the child eat:**									
	**Potato Crisps**		**3417**	**(See Manual)**						
	**Peanuts**	Roasted Unsalted	**3452**							
		Roasted, Salted	**3458**							
	**Cheese Curls (Nik Naks, etc.)**	Average	**3267**							
		Savoury	**3418**							
	**Popcorn**	Plain	**3332**							
		Sugar Coated	**3359**							
	**Peanuts and Raisins (mixed)**	Roasted, Salted	**P0047**							
	**Chocolates**	Specify types and names: Assorted	**3992**							
	**Candies**	Sugus, gums, hard sweets (Specify)	**3986**							
	**Sweets**	Toffee, fudge, caramels (Specify)	**3991**							
	**How many times a week does the child eat snack food? ____________**									
CAKES, BISCUITS AND COOKIES	**Biscuits/Cookies**	Specify Type		**(See Manual)**						
	**Cakes & Tarts**	Specify Type								
	**Pancakes/Crumpets**	Specify Type								
	**Rusks**	Specify Types								
	**Scones**	White, WM	**3237**	6cm diam=35g; 8cm diam=60g						
	**Muffins**	Plain	**3408**							
		Bran	**3407**							
	**Koeksisters**		**3231**	100 x 35 = 60g						
	**Savouries**	Sausage Rolls	**2939**	Roll x 135mm = 165g						
		Samoosas (Meat)	**3355**	S/s = 42g						

	FOOD	DESCRIPTION	CODE	QUANTITY (g/ml)	AMOUNT USUALLY EATEN (HHM)	AMOUNT USUALLY EATEN (g)	P/D	D/W	P/M	SEL/NEV
		Biscuits e.g. Bacon Kips	**3331**	4g						
		Other								
	**How many times a week does the child eat cakes/cookies? ______ less than 1/week _______**									
PUDDINGS	**Jelly**		**3983**	1T=35g; 1SP=75g; ½ c = 110g						
	**Baked Puddings**	Specify Types		Med serving = 30g<br>30 x 65 x 65 = 50g						
	**Instant Puddings**	Specify Types		1T = 45g; SP = 95g;<br>½ c =145g						
	**Infant Deserts**	Specify Types		Jar=200g; 1t = 11g;<br>½ c = 125g						
	**Ice Cream**	Commercial Regular	**3483**	Scoop = 40g; 1SP=65g;						
		Commercial Rich	**3519**	½ c = 75g						
		Soft serve	**3518**	Plain = 135g; + flake = 155g						
		Sorbet	**3491**	Scoop = 40g; 1SP=65g;						
		Ice Lollies	**3982**	½ c = 75g						
		Chocolate Coated Individual Ice Creams (E.g. Magnum)	**P0036**							
	**Custard**	Home Made (WM)	**2716**	T=13g; SP = 40g						
		(SM)	**2717**							
	**Other Puddings**<br>**Specify**									
	**How many times a week does the child eat pudding? ______ less than 1/week _______**									
SAUCES, GRAVIES, CONDIMENTS	**Tomato Sauce**		3139	1t = 6g; 1T = 25g						
	**Worcester Sauce**		P0037							
	**Chutney**	Fruit	3168	1t = 14g; 1T = 60g						
		Tomato	3114							
	**Pickles**		3866	1 = 10g						
	**Packet Soups**		3165	½ c = 125g						
	**Others**									

	FOOD	DESCRIPTION	CODE	QUANTITY (g/ml)	AMOUNT USUALLY EATEN (HHM)	AMOUNT USUALLY EATEN (g)	P/D	D/W	P/M	SEL/NEV
	**Wild birds, animals, insects or fruits and berries (hunted or collected in rural areas or on farms): Specify**									
	**Please mention any other foods eaten by the child more than once every two weeks which we have not talked about or foods eaten in other homes or places during the past week.**									

1. Are there any foods that the child does not eat? Please list them and give reasons why the child does not eat them (e.g. because of religious beliefs).

FOODS NOT EATEN	CODES	REASON

	YES	NO	DON'T KNOW
2. For the main meal, do you buy, and/or cook and/or serve the child's food separately from that of adults in the house?	1	2	3

3. EATING PATTERNS: (FREQUENCY OF EATING)<br>Please indicate which of the following best describes the eating pattern the child usually follows **(mark only one)**	
More than three meals with eating between meals	1
Three meals with eating between meals	2
Three meals with no eating between meals	3
Two meals with eating between meals	4
Two meals with no eating between meals	5
One meal with eating between meals	6
One meal with no eating between meals	7
Nibble the whole day, no specific meals	8
Others (Please specify):	9

4. Are there any foods that the child eats which we haven't talked about? Please list them.

FOODS	DESCRIPTION	AMOUNT USUALLY EATEN	TIMES EATEN				CODE
			Per day	Per week	Per month	Seldom/ Never	

5. Does your child sometimes eat elsewhere? YES ☐ NO ☐

6. If yes, for what reason?

(1)	(2)	(3)	(4)	(5)	(6)	(7)
Take food to school	Buy food at school	School nutrition program	Creche	Eating out	Eat with relatives or friends	Other

7. How often?

(1) > once a week	(2) weekly	(3) monthly	(4) < once a month

8. Indicate where:

(1) Family	(2) Friends	(3) Café	(4) Restaurant, Fast food	(5) School
(6) Other (specify):				

Thank you for your co-operation. We appreciate your contribution.

ABBREVIATIONS:

<u>Measures</u><br>1t = 1 rounded teaspoon<br>1T = 1 rounded tablespoon (15ml)<br>1SP = 1 rounded servingspoon (30ml)<br>c = measuring cup (250ml)<br>s/s = small size<br>m/s medium<br>L/s = large<br>E = enriched<br>P = plain<br><br><u>Milk</u>:<br>SM = skim milk<br>WM = whole milk<br>BL = blend<br>CON = condensed	B<u>read</u>:<br>Wh = white<br>Br = brown<br>Ww = wholewheat<br><br><u>Meat</u>:<br>F = with fat<br>FT = fat trimmed<br><br><u>Oil/Fat</u><br>B = butter<br>HM = hard margarine<br>Med = medium fat/light<br>PM =- polyunsaturated<br>SO = sunflower oil<br>WF = white fat<br>PB = peanut butter	BR = breakfast (Up to 09h00)<br>IS = in-between snack<br>L = lunch (midday (12h00-14h00)<br>D = dinner (evening) (17h00 - 19h00)<br>AD = after dinner<br>Comm = commercial<br>Home = homemade<br>Pot = potato<br>Cab = cabbage<br>Carr = carrot<br>Fill = filling<br>Usually = at least 4x/week<br><br><u>Other</u><br>HHM = Household Measure<br>P/D = Per day<br>D/W = Days Per Week<br>P/M = Per Month<br>SEL/NEV = Seldom / Never

3. Expected risks and corresponding precautionary measures

This study will be conducted in accordance with internationally accepted ethical standards and guidelines, including the International Conference on Harmonization for GCP, and the Declaration of Helsinki (version 2013). Ethics committee of both the ETH Zurich, in Switzerland, and the Health Research Ethics Committee of Stellenbosch University, in South Africa, will approve the protocol and all procedures involving human study participants.

Detailed oral and written information explaining the study purposes, potential risks and benefits will be provided to the participants and their caregivers.

The participation includes the small risks of bruise, infection and/or phlebitis, and discomforts during having blood drawn from the child. These risks will be explained to the caregivers and children. Nonetheless, morbidity of the children will be closely monitored weekly during study 1 and biweekly during study 2. Participants becoming severely anemic (Hb <8 g/dL) at any time during the study (studies 1 and 2) will receive standard of care treatment according to local guidelines, but will not be excluded from the study. Participants developing secondary infections at any time during the study will receive standard of care treatment and iron may be withheld for several days until the appropriate treatment is complete, but the participants will not be excluded.

There is a chance that some of the participants may experience gastrointestinal side effects of the iron supplementation (iron tablet). These could include epigastric discomfort, vomiting, and/or constipation. It is also possible that a child could have a food allergy to a component of the LNS or fortified meal. Therefore, we exclude children with known lactose and/or peanut allergies.

The benefits of the study include the iron supplementation, which is likely to have a positive effect on the child's iron status. Furthermore, the child's health will be monitored closely throughout the study and sick children will be treated free of charge at the Tygerberg Children's Hospital. Caregivers will receive an allowance to compensate for the invested time and for transport costs (R150 – 300 / 10 – 20 €) per study visit, depending on the distance traveled, the length of the stay and the number of samples needed. Furthermore, the investigators believe that this study will directly benefit the community from which the data is drawn since it will make recommendations about best practice within public sector clinics.

Safety aspects of measurement of iron absorption

Within the study, the children will receive two forms of naturally occurring iron differing only in their weight (^{57}Fe, ^{58}Fe). These two forms of iron (isotopes) are naturally present in fixed amounts in the environment and in foods. Iron absorption is measured by altering the ratio of these forms of natural iron in a specially prepared test meal. These forms of natural iron are entirely safe and stable (non-radioactive) and their consumption presents no health risks. This technique is widely used to measure iron absorption and is the gold standard to assess iron bioavailability in humans [65]. The Institute of Food, Nutrition and Health has used this technique in numerous studies worldwide, including studies in pre-school Kenyan [66] and Beninese children [53], European infants [67], school aged Jamaican children [68], and Swiss, Rwandese, Beninese and Thai women of reproductive age [25, 50, 60, 69-71].

Blood sampling

Blood withdrawal will be done by pediatric technicians or nurses with extensive experience with child venipuncture in the Tygerberg Children's Hospital, using sterile equipment and technique. The small risk of irritation, minor bruising, inflammation or phlebitis after venipuncture will be explained to the caregivers of the participating child. Since the children are under active surveillance, the venipuncture site will be checked at the next visit. The purpose of the blood withdrawals in the study will be clearly explained to the caregivers and children, as well as the methods through which these samples will be examined and stored. Results of blood analysis (Hb) will be communicated and explained to the caregivers.

Severely anemic and severely underweight

During recruitment, Hb and BMI will be assessed. Severely anemic or severely underweight children will not be included in the study and will be referred to the pediatrics at Tygerberg Children's Hospital for appropriate treatment free of charge.

Stool sampling

The collection of stool samples entails no risk for the child.

Safety aspect of interventions

No study drug will be administered in the study. The interventions consists of a traditional maize based meal, a LNS and oral $FeSO_4$ supplements. An increased risk for infectious disease, such as malaria or diarrhea, from using iron supplements is not expected. Nonetheless, morbidity of the children will be closely monitored and infectious diseases, such as malaria and diarrhea, will be surveyed and treated according to the national guidelines.

Illness during study

Caregivers will be instructed to bring children that fall sick during the study to the nearest appropriate facility, where children will be examined, and to report us of the event. Furthermore, morbidity will be assessed weekly in study 1 and bi-weekly using phone calls in study 2; and AEs will be reported. Participants becoming severely anemic (Hb <8 g/dL) at any time during the study (studies 1 and 2) will receive standard of care treatment according to local guidelines, but will not be excluded from the study. Participants developing secondary infections at any time during the study will receive standard of care treatment and iron may be withheld for several days until the appropriate treatment is complete, but the participants will not be excluded.

Monitoring

The investigators are responsible for the validity of all data collected at the study site and must accept the various monitoring procedures employed by the PI. A risk based monitoring approach will be followed. The purpose of monitoring is to verify that the rights and well-being of participants are protected; that trial data are accurate, complete and verifiable with source data; and that the trial is conducted in compliance with the protocol, International GCP, the ethical principles that have their origin in the Declaration of Helsinki and the applicable regulatory requirements.

Monitors assigned by the PI will conduct regular site visits for monitoring various aspects of the study. Visits will take place usually within a predetermined interval, but this may vary during the study. The co-investigators and site staff will allow the study monitor and authorized representatives of the PI to (1) inspect all eCRFs, written ICFs and corresponding source documents (e.g. original medical records), participant records and laboratory raw data, and (2) access clinical supplies, dispensing and storage areas. The investigators and site staff should also agree to assist with monitoring activities if requested and provide adequate time and space for monitoring visits.

The monitor will query any missing, confusing, spurious, or otherwise ambiguous data with the investigator. All queries should be resolved in a timely manner. A monitoring log will be maintained recording each visit, the reason for the visit, the monitor's signature and Investigator or designee's confirmation signature.

Data safety monitoring board (DSMB)

The primary responsibility of the DSMB will be to act in an advisory capacity to the PI to safeguard the interests of trial participants by monitoring participant safety, assess participants risk versus benefit, and assess data quality and general evaluation of the trial progress. The activity of the DSMB will be delineated in a charter that will define the membership, responsibilities and the scope and frequency of data reviews. The DSMB will operate on a conflict-free basis independently of the PI and the study team. The DSMB will comprise two nutritionists from Switzerland (Dr. Isabelle Herter-Aeberli, ETH Zurich, Switzerland; the second nutritionist still needs to confirm), two scientists from the South African team (Dr. Mark Cotton, Stellenbosch University, South Africa; Dr. Shaun Barnabas, Stellenbosch University, South Africa) and one hematologist/pediatrician (Prof. Dr. med Gary Brittenham, Colombia University, USA).

The DSMB may have an organizational meeting prior to commencement of the trial. The DSMB will have one meeting where it will review data during a closed session. This meeting is planned to occur once 33% of the planned participants' complete treatment. The PI or the DSMB may convene ad hoc meetings if safety concerns arise during the trial. After its assessment, the DSMB will recommend to the PI continuation, modification or termination of the study.

Definition and reporting of adverse events (AEs)

During the entire duration of the study, all AEs and all SAEs are collected, fully investigated and documented in eCRFs. Study duration encompassed the time from when the participant signs the informed consent until the last protocol-specific procedure has been completed, including a safety follow-up period for recently reported AEs or SAEs.

Definition

Adverse Event (AE)

An AE is any untoward medical occurrence in a patient or clinical investigation, which does not necessarily have a causal relationship with the investigational product. An AE can therefore be any unfavorable and unintended sign (including an abnormal laboratory finding), symptom, or disease temporally associated with the use of an investigational product, whether related to the investigational product.

Serious Adverse Event (SAE)

Any untoward medical occurrence that at any dose:

- results in death;
- is life threatening (any event in which the participant was at risk of death at the time of the event; it does not refer to an event, which hypothetically might have caused death if it were more severe);
- requires inpatient hospitalization or prolongation of existing hospitalization;
- results in persistent or significant disability/incapacity;
- is a congenital anomaly/birth defect; or
- is a medically important event.

<u>Note:</u>

Medical and scientific judgment should be exercised in deciding which is a medically important event that may not be immediately life-threatening or result in death or hospitalization, but may jeopardize the participant or may require medical or surgical intervention to prevent one of the outcomes listed above. A “suspected transmission of infectious agent by a medicinal product” is also considered a SAE under the SAE criterion “Other medically important condition”.

Unlisted (Unexpected) Adverse Event (AE)

An adverse reaction, the nature or severity of which is not consistent with the applicable product information.

Suspected Unexpected Serious Adverse Reactions (SUSARs)

An adverse reaction that is both unexpected (not consistent with the applicable product information) and meets the definition of a SAE/Reaction. The PI evaluates any SAE that has been reported regarding seriousness, causality and expectedness. If the event is related to the investigational product and is both serious and unexpected, it is classified as a SUSAR. The occurrence of SUSARs in the present studies is unlikely.

Life threatening

Any event in which the participant was at risk of death at the time of the event; it does not refer to an event, which hypothetically might have caused death if it were more severe.

Attribution/Causality

An AE is considered associated with the use of the iron supplements and test meals if the attribution is possible, probable or very likely. The definitions for rating attribution/causality will be as described below:

Not Related: An AE, which is not related to the use of the supplement.

Unlikely: An AE for which an alternative explanation is more likely, e.g. concomitant drug(s) or concomitant disease(s), and/or the relationship in time suggests that a causal relationship is unlikely.

Possible: An AE, which might be due to the use of the supplement. An alternative explanation, e.g. concomitant drug(s) or concomitant disease(s), is inconclusive. The relationship in time is reasonable; therefore, the causal relationship cannot be excluded.

Probable: An AE, which might be due to the use of the supplementation. The relationship in time is suggestive, e.g. confirmed by dechallenge. An alternative explanation is less likely, e.g. concomitant drug(s) or concomitant disease(s).

Certain: An AE, which is listed as a possible adverse reaction and cannot be reasonably explained by an alternative explanation, e.g. concomitant drug(s) or concomitant disease(s).

Severity

The scale described below is to be used to estimate grade of AE severity:

GRADE 1 Mild transient or mild discomfort (< 48 hours); no medical intervention/therapy required.

GRADE 2 Moderate mild to moderate limitation in activity - some assistance may be needed; no or minimal medical intervention/therapy required.

GRADE 3 Severe marked limitation in activity, some assistance usually required; medical intervention/therapy required, hospitalizations possible.

GRADE 4 Potentially life-threatening extreme limitation in activity, significant assistance required; significant medical intervention/therapy required, hospitalization or hospice care probable.

Other relevant adverse event (AE) Definitions

The following definitions will be used for AE reporting:

Action Taken with iron supplements

- Supplement unchanged
- Supplement interrupted
- Supplement stopped
- Not applicable (Follow-up period)

Other Action taken

- None
- Medication given
- Hospitalization or prolongation of hospitalization
- Therapeutic or diagnostic procedure

Outcome

- Resolved
- Improved
- Unchanged
- Worse
- Fatal
- Unknown

Occurrence

- Once
- Intermittent
- Continuous

Reporting

All AEs will be reported to the PI. All SAE will be reported by the co-investigators to the study monitor and the PI within 24 hours of the site being aware of the SAE, whether the serious event is deemed associated with the use of the drug.

Adverse Event (AE)

All AEs and SAEs will be collected by the investigator from the time a participant signs the ICF up to the study end. Any AE (serious or non-serious) observed by the investigator or reported by the participant will be recorded in an AE eCRF. The investigator will review each AE and assess its relationship to the iron supplements and test meals based on all available information at the time of the completion of the eCRF. The following information will be recorded for each AE reported:

- Diagnosis of the AE, if possible. In the case where an overall diagnosis cannot be made, each specific sign and/or symptom will be recorded as individual AEs;
- Date of onset;
- Stop Date (duration) if applicable;
- Severity;
- Action Taken with iron supplements;
- Other Action Taken;
- Outcome;
- Relationship to iron supplements;
- Occurrence;
- Seriousness.

Serious Adverse Event (SAE)

Any SAE that occurs, which is serious, must be reported by the investigators to the study monitor and copied to the PI within 24 hours of the site first being aware of the SAE, whether the serious event is deemed associated with the use of the drug.

In addition, the investigator will provide a detailed, signed, written, and complete SAE report form that addresses the investigator's estimates of the attribution/causality of the SAE to the study drug in question to the study monitor within 24 hours of becoming aware of the SAE.

The study monitor will confirm receipt of the SAE Form with the investigator and review the initial information on the SAE for diagnosis, consistency and completeness of data.

For submission of updated or additional information on a previously reported SAE, the investigator will provide the study monitor and medical monitor with a newly completed SAE form, designated as a follow-up report. This will be submitted to the study monitor and medical monitor within 24 hours of the investigator receiving the information.

The study monitor will query for additional information from the investigator, if necessary, to complete the profile of the SAE reported.

The PI/co-investigator/designee will inform CEC (JKUAT IERC) of all SAEs in accordance with local requirements and ICH guidelines for GCP. SAEs (including SUSARs) resulting in death are reported to the

JKUAT IERC within 7 days. SUSARs that are not fatal will be reported within 15 days. The PI/designee will forward Safety Notification letters to the investigator for submission to the CEC (JKUAT IERC).

Follow up of Adverse Events (AEs)

All AEs will be followed until:

- Satisfactory clinical resolution or stabilization; or
- Until the end of the follow-up period; and
- Until all queries on these AEs have been resolved.

Certain long-term AEs cannot be followed until resolution within the setting of this protocol. In these cases, follow-up will be the responsibility of the treating physician. However, this will have to be agreed upon with the PI.

Post-Trial Adverse Events (AEs)

Any new SAEs reported by the participant to the investigator that occur after the last scheduled contact, and are determined by the investigator to be possible, probable or certainly related to the use of the iron supplements and test meals, will be reported to the PI, CECs on an expedited basis as required in accordance with local requirements and ICH guidelines for GCP.

Clinical Laboratory Adverse Events (AEs)

Changes in the results of the Clinical Laboratory assessment results, which the investigator feels are clinically significant, will be reported as AEs (e.g. decrease in Hb throughout the study). It is the investigators' responsibility to review the results of all laboratory tests as they become available. This review must be documented by the investigators' dated signature on the laboratory report. For each abnormal laboratory test result, the investigator needs to ascertain and document if this is a clinically significant change from baseline for that individual participant. This determination, however, does not necessarily need to be made the first time an abnormal value is observed.

The investigator may repeat the laboratory test or request additional tests to verify the results of the original laboratory tests. If this laboratory value is determined by the investigator to be a clinically significant change from baseline for that participant, it is considered an AE.

4. Curricula vitae

a. Curriculum vitae of principle investigator
b. Publication list of principle investigator (from the past 5 years)
c. Curricula vitae of all collaborators

See attachments

5. ***Participants***

a. Exact number of participants

180 children, 8-12 years old

b. Selection and exclusion criteria

Inclusion criteria

- Age 8-12 years at baseline
- Hb ≥8 g/dL
- BMI −3 to 2 SD of reference population
- HIV criteria: sCD4 ≥500 cells/mm^3, HIV RNA viral load <50 copies/mL (measured as part of routine care)
- PF <30 µg/L
- The caregiver is willing to participate in the study.
- Caregiver and participant speak English, Afrikaans or isiXhosa.
- The informed consent form has been read and signed by the caregiver (or has been read out to the caregiver in case of illiteracy) plus assent needs to be obtained from the child.
- Residence in the study site for the period of the study.

For non-iron deficient children (3rd and 4th group):

- Hb ≥11.5 g/dL
- PF ≥30 µg/L

Exclusion criteria:

- Iron supplements 4 weeks prior to study start.
- Food allergy or intolerance against peanuts or milk.
- Acute illness or other conditions that in the opinion of the PI or co-researchers would jeopardize the safety or rights of a participant in the trial or would render the participant unable to comply with the protocol.

c. Recruitment of participants:

The study participants will be recruited from the Cape Town long-term birth cohort, the FAM CRU, of perinatally HIV infected children and uninfected controls. The FAM CRU is located inside the Tygerberg Academic Hospital complex in Cape Town. The cohort undergoes extensive annual assessment, having exited two local clinical trials a few years ago during which they were very carefully watched. After a baseline assessment comparing iron and inflammatory status, we will recruit iron deficient children from each group.

The participants in these studies will mainly be recruited from the FAM-CRU long-term ART birth cohort including:

- Perinatally HIV infected children 8-12years old who initiated very early ART within the first few weeks after delivery (n=71).

- Perinatally HIV infected children 8-12 years old who initiated ART at 6-36 months of age (n=67).
- HIV-exposed uninfected (HEU) children from the same communities and socioeconomic background (n=68).
- HIV-unexposed uninfected (HU) children from the same communities and socioeconomic background (n=74).
- Newly recruited HIV infected children 8-12 years from the same communities and socioeconomic background who are not from the FAM-CRU long-term ART birth cohort (n = depends on the number of included children from the FAM-CRU and the required sample size).

6. Information sheet for participants

PARTICIPANT INFORMATION LEAFLET AND CONSENT FORM

TITLE OF THE RESEARCH PROJECT:

A novel stable iron isotope method to define iron needs and improve iron nutrition in HIV-positive and HIV-negative children.

REFERENCE NUMBER: M18/05/017

PRINCIPAL INVESTIGATORS: Dr S Barnabas, Prof MB Zimmermann, Prof R Blaauw

ADDRESS: FAMCRU, Ward J8, Tygerberg Hospital

CONTACT NUMBER: 021 938 4295

You are being invited to take part in a research project. Please take some time to read the information presented here, which will explain the details of this project. Please ask the study staff or doctor any questions about any part of this project that you do not fully understand. It is very important that you are fully satisfied that you clearly understand what this research entails and how you could be involved. Also, your participation is **entirely voluntary** and you are free to decline to participate. If you say no, this will not affect you negatively in any way whatsoever. You are also free to withdraw from the study at any point, even if you do agree to take part.

This study has been approved by the **Health Research Ethics Committee at Stellenbosch University** and will be conducted according to the ethical guidelines and principles of the international Declaration of Helsinki, October 2013, South African Guidelines for Good Clinical Practice and the Medical Research Council (MRC) Ethical Guidelines for Research.

What is this research study all about?

- This study will involve 180 children aged 8 to 12 years and will be done at the FAMCRU unit.
- In this study, we want to learn how to improve iron intake in HIV-negative and HIV-positive children to prevent low iron levels and thereby improving development and health.
- If you are willing to participate and your child fulfils all the inclusion criteria, you have to sign the informed consent form. As a participant, you will be asked to bring your child to the FAMCRU unit at Tygerberg Hospital for 7 to 10 study visits, depending on the iron status of your child, over a period of around 15 months.
- You will see from the study title that we will be studying a stable iron isotope method. This is a description for specific forms of iron that differ in weight. We will add these different forms of iron to the three test meals that your child will receive. These forms of iron are entirely safe, have been used in many other studies, and will not harm your child's health in any way.

The following will happen at every visit:

- **Study visit 1:** We will take a blood sample (7.5 mL = 2 teaspoons) from your child to see how much iron and signs of infection your child has in his or her blood. During this visit, we will also look at your child's health, and measure your child's height and weight.
- **Study visit 2, 3 and 4:** If your child has ***low iron levels*** based on the blood sample taken on visit 1, we will ask you and your child to participate on study visits 2, 3 and 4. On these visits, you will be asked to come to the

hospital at 7 am in the morning. Your child has to be fasted, meaning that the evening before the visits, no foods are allowed after 10 PM and no drinks after midnight. We will do a short interview with you and your child about the foods typically eaten. On visits 2 and 3, we will look at your child's health and your child will eat a special form of iron added to maize porridge or peanut butter. On visit 4, we will take a blood sample (3 mL = 1 teaspoon) from your child to see how much iron from the maize porridge and the peanut butter your child has taken up in the body. We will also look at your child's health, weight and height. Then, your child will drink an iron tablet together with a glass of water. After your child has eaten the maize porridge, peanut butter or drunk the iron tablet, you and your child will be asked to stay at the hospital for 3 hours and your child should not eat or drink during these 3 hours. During this time, we encourage that all children do school work (please arrange the day before and bring with) or homework. After the three hours on visits 2, 3 and 4, your child will receive a meal free of charge.

If your child ***does not have low iron levels***, then he or she will not eat the maize porridge and peanut butter but rather drink a fruit juice with the special form of iron added on visit 3, and skip visits 2 and 4. After your child has drunk the fruit juice, you and your child will be asked to stay at the hospital for 3 hours and your child should not eat or drink during these 3 hours. After the three hours, your child will receive a meal free of charge.

On study visit 3 ***(all),*** we will have an interview with you and your child about the foods typically eaten.

On study visit 3 ***(if iron levels are low)*** or 5 ***(if iron levels are not low),*** we will provide you with a container to collect a stool sample from your child at home and bring with on study visit 5. Stool sample collection will be explained to you and your child in detail by the study staff. This sample and the following one will be used to look at signs of infection and the balance of healthy and unhealthy bacteria in the gut of your child.

- **Study visit 5:** You will bring the stool sample collected at home. We will take a blood sample (7.5 mL = 2 teaspoons) from your child to see how much iron from the iron tablet your child has taken up. We will also see how much iron and zinc your child has in his or her blood and look for any signs of infection. We may perform a second interview with you and your child about the foods typically eaten. Additionally, we will look at your child's health, weight and height and provide you with a container to collect a stool sample at home and bring with on study visit 6. If your child has low iron levels in the blood, he or she will consume iron tablets for 3 months. Your child should take the iron tablet provided every day. We will give you a quick and easy diary to complete every day and we will follow-up with phone calls every second week.

- **Study visit 6:** You will bring the stool sample collected at home. We will take a blood sample (2 mL = 1 teaspoon) to look for any signs of infection and to measure the amount of iron in your child's blood.

- **Study visit 7, 8, 9 and 10:** We will take blood samples (7.5 mL = 2 teaspoons) from your child four times in the next 14 months during study visits 7, 8, 9 and 10 (5 months, 9 months, 12 months and 15 months after the start of study) and look at your child's health. We will monitor the health of your child every month by phone calls and will ask if your child had any signs or symptoms of illness. If at any point your child is found to have low iron levels in the blood, we will provide iron tablets.

For study visits 5 to 10, please ask you child not to eat anything 2 hours before the visit.

The blood and stool samples collected will be sent to laboratories in Switzerland and Belgium for the different tests.

	DAY	VISIT	Children with low iron levels	Children without low iron levels
			AT THE HOSPITAL	
**SCREENING**	- 1	**1**	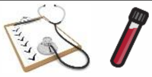	
**START OF STUDY**	1	**2**	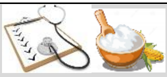	
	3	**3**	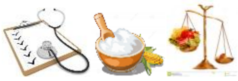	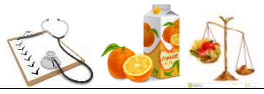
	17	**4**	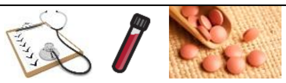	
	31<br>(1 month)	**5**	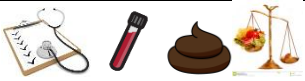	
	121<br>(4 months)	**6**	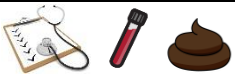	
	151<br>(5 months)	**7**	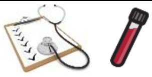	
	271<br>(9 months)	**8**	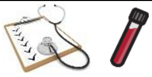	
	361<br>(12 months)	**9**	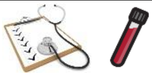	
**ENDPOINT**	451<br>(15 months)	**10**	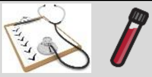	

Key:

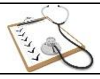	Look at child's health, questions, weight and height
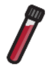	Blood sample
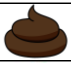	Stool sample
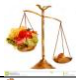	Interview: Foods typically eaten
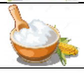	Maize porridge or peanut butter
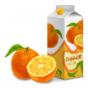	Fruit juice
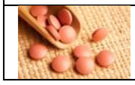	Iron tablets

Why have you been invited to participate?

- In order to improve iron levels in children, we need to study children with and without low iron levels, and with and without HIV. We think that your child might fit the inclusion criteria for any of these groups and that is why we invite you and your child to consider taking part in this study.

What will your responsibilities be?

If you are participating in the study, you will have to respect certain rules. They are necessary to make the study successful.

- Be at the hospital in time for the scheduled appointments.
- Comply with the study plan.
- Inform the person in charge of the study of any disease, infection, symptoms, pain or change of condition in your child.
- Inform the person in charge of the study about other treatments and medicines.

Will you benefit from taking part in this research?

- The benefit of the study is that your child will get the tests that check to see if it has enough red blood cells and iron in the body. Furthermore, your child's health will be closely watched throughout the study and sick children will be treated free of charge. This study will also benefit other children in future since we hope to find ways in preventing low iron levels.

Are there any risks involved in your taking part in this research?

- Participation includes the small risks of bruising, infection, and discomfort during blood drawn from the child. To minimize these risks, trained and experienced nurses will draw blood using sterile equipment and techniques. A cream, Emla, will be used to numb the skin before each blood draw.
- There is no risk associated with the special forms of iron, they are entirely safe, and their consumption presents no health risks.
- If your child has low iron levels, then he or she will miss three school days since there are three visits in the morning. If your child does not have low iron levels, then he or she will miss one school day since there is one morning visit. All other visits are in the afternoon. Please arrange with the teacher for school work before the visit(s). In the 3-hour waiting time after the test meal, we will encourage the children to complete their school work. Should your child not be able to attend any of the visits due to compulsory tests or exams, then we will reschedule the visit.
- Should you as caregiver not be able to attend, then another adult family member or friend may attend the visit with your child, and if no one is available, we will reschedule the visit. We will issue a letter to confirm attendance of the study visit should your employer require a letter.

If you do not agree to take part, what alternatives do you have?

- You and your child will only participate in this study if you are both willing to participate. No one can force you or your child in any other way to participate. You will not have to comment on why you or your child do not want to participate. If you or your child have made your decision, your child can withdraw from the study at any time, without giving reasons and without consequences. If your child has low iron levels and you or your child decide to withdraw at any time, iron tablets can be accessed at any primary health care clinic.

Who will have access to your medical records?

- The researchers will treat all information collected as private and confidential. This means that your name or your child's names will not appear on the research forms or any of the reports. A separate list which connects your name to the study number will be kept locked in a separate file from the data and will not be seen by anyone except the study team. Your and your child's identity will not be given when our findings are communicated, including publication of the data in science journals. Only information about the entire group that participated will be reported. No information about you or your child, or information that connects you

to the study, will be given to anyone without your wish and your written permission. At any time during the study, you can ask to see your child's data.

What will happen in the unlikely event of some form of injury occurring as a direct result of your taking part in this research study?

- In the unlikely case of harm associated with the study, you child will receive immediate medical treatment. All cost for medical treatment caused by harmful consequences of the study will be covered by insurance from Stellenbosch University. Accidents on the way to and from Tygerberg Hospital are not included in this insurance.

Will you be paid to take part in this study and are there any costs involved?

- You as caregiver will receive an incentive for your time, inconvenience and expenses for each scheduled study visit. Your child will receive age-appropriate gifts at selected visits as note of thanks. There will be no costs involved for you, if you do take part.

Is there anything else that you should know or do?

- You should inform your family practitioner or usual doctor that you are taking part in a research study.
- You can contact Dr. S Barnabas on 021 938 5295 or after hours on 084 641 0735 if you have any further queries or problems.
- You can contact the Health Research Ethics Committee at 021 938 9207 if you have any concerns or complaints that have not been adequately addressed by your study doctor/staff.
- You will receive a copy of this information and consent form for your own records.

Declaration by participant

By signing below, I *(name, surname)* ...., legal guardian of *(child's name, surname)* ...., agree to take part in a research study entitled: *A novel stable iron isotope method to define iron needs and improve iron nutrition in HIV-positive and HIV-negative children.*

I declare that:

- I have read or had read to me this information and consent form and it is written in a language with which I am fluent and comfortable.
- I have had a chance to ask questions and all my questions have been adequately answered.
- I understand that taking part in this study is **voluntary** and I have not been pressurised to take part.
- I may choose to leave the study at any time and will not be penalised or prejudiced in any way.
- I may be asked to leave the study before it has finished, if the study doctor or researcher feels it is in my best interests, or if I do not follow the study plan, as agreed to.

Signed at (*place*) .... on (*date*) .... at (*time*) ....

....

Signature of participant Signature of witness

Declaration by investigator

I *(name, surname)* .... declare that:

- I explained the information in this document to *(name, surname of child's legal guardian)* ....
- I encouraged him/her to ask questions and took adequate time to answer them.
- I am satisfied that he/she adequately understands all aspects of the research, as discussed above
- I did/did not use an interpreter *(if an interpreter is used then the interpreter must sign the declaration below).*

Signed at (*place*) .... on (*date*) .... at (*time*) ....

....

Signature of investigator Signature of witness

Declaration by interpreter

I *(name, surname)* .... declare that:

- I assisted the investigator (*name, surname*) .... to explain the information

  in this document to (*name, surname of child's legal guardian*) ....

  using the language medium of Afrikaans/Xhosa.

- We encouraged him/her to ask questions and took adequate time to answer them.
- I conveyed a factually correct version of what was related to me.
- I am satisfied that the participant fully understands the content of this informed consent document and has had all his/her question satisfactorily answered.

Signed at (*place*) .... on (*date*) .... at (*time*) ....

....	....
Signature of interpreter	Signature of witness

PARTICIPANT INFORMATION LEAFLET AND ASSENT FORM FOR CHILDREN

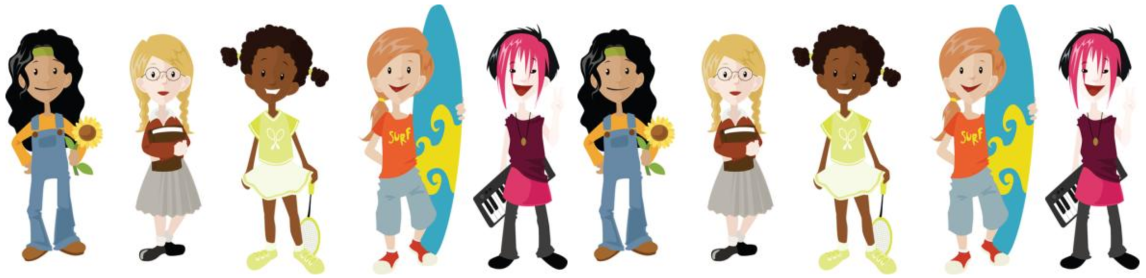

TITLE OF THE RESEARCH PROJECT: A new way to find out how much iron is needed by our bodies and how to improve iron intake in HIV-positive and HIV-negative children.

RESEARCHERS NAME(S): Dr S Barnabas, Prof MB Zimmermann, Prof R Blaauw

ADDRESS: FAMCRU, Ward J8, Tygerberg Hospital

CONTACT NUMBER: 021 938 4295

Hello, my name is *(name, surname)* and I would like to ask if you would like to take part in a research project.

What is research?

- Research is something we do to find new answers about the way things (and people) work. We use research projects or studies to help us find out more about disease or illness. Research also helps us to find better ways of helping or treating children who are sick.

What is this research project all about?

- Food has building blocks to keep us healthy and to help us grow. We want to find out more about iron, one of the building blocks in food, and see how much iron is needed by our bodies and how we can prevent low levels of iron.

Why have I been invited to take part in this research project?

- To find the answers we are looking for, we need to do a few tests in children with and without low iron levels, and with and without HIV. We think that you might fall into any of these groups and that is why we ask you to think about taking part in this study.

Who is doing the research?

- The research is done by researchers from Stellenbosch University (Tygerberg) and a university in Switzerland in Europe, ETH Zurich.

What will happen to me in this study?

- There will be 180 children from 8 to 12 years in this study.
- If you say yes to take part, then you have to sign a special form. As a participant, you will visit the FAMCRU unit at Tygerberg Hospital 7 to 10 times over a period of 15 months, depending on how much iron is in your blood.
- The visits will not be the same each time and the following will happen at different visits:
    - We will look at your health and ask some questions.
    - We will measure your weight and height.
    - We will draw blood.

- We will ask you to bring a stool (poo) sample (this you will collect at home and bring with in a closed plastic container).
- We will talk about foods that you eat on most days.
- You will eat maize porridge with special iron once if your iron levels are low.
- You will eat peanut butter with special iron once if your iron levels are low.
- You will drink iron tablets with special iron once if your iron levels are low.
- You will drink fruit juice with special iron once if your iron levels are not low.
- You will drink iron tablets for 3 months if you have low iron levels.

The blood and stool samples collected will be sent to laboratories in Switzerland and Belgium for the different tests.

			**Children with low iron levels**	**Children without low iron levels**
	**DAY**	**VISIT**	**AT THE HOSPITAL**	
**SCREENING**	- 1	**1**	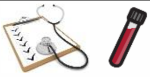	
**START OF STUDY**	1	**2**	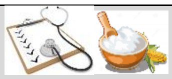	
	3	**3**	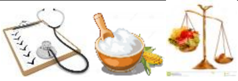	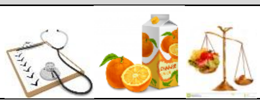
	17	**4**	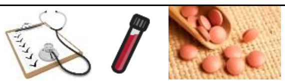	
	31 (1 month)	**5**	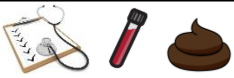	
	121 (4 months)	**6**	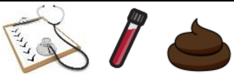	
	151 (5 months)	**7**	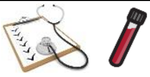	
	271 (9 months)	**8**	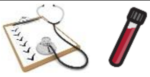	
	361 (12 months)	**9**	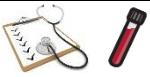	
**ENDPOINT**	451 (15 months)	**10**	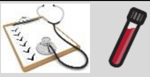	

Key:

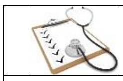	Look at child's health, questions, weight and height
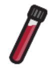	Blood sample
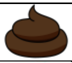	Stool sample
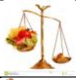	Interview: Foods typically eaten
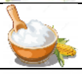	Maize porridge or peanut butter
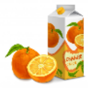	Fruit juice
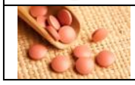	Iron tablets

Can anything bad happen to me?

- There is a small chance of bruising, infection, and soreness during blood draw. Only a trained nurse will draw blood. A cream will be used to numb the skin before each blood draw.
- If the iron level in your blood is low, then you will miss three school days since there are three visits in the morning. If the iron level in your blood is not low, then you will miss one school day since there is one morning visit. All other visits are in the afternoon. Please arrange with the teacher for school work before the visit(s). In the 3-hour waiting time after the test meal, we will encourage you to complete your school work. Should you write a test or exam on your visit date, then we will reschedule the visit.

Can anything good happen to me?

- Yes, you will know how much iron you have in the body. We will also keep a close eye on your health and treat any illness. Other children can also be helped in future if we find out how to prevent low iron levels.

Will anyone know I am in the study?

- No, the researchers will not tell anyone and your information will only be seen by the research team. Your name will not be on any forms or reports. When we share the information of the group with others, no names will be mentioned.
- Please ask your teacher for school work for the morning visits, and please bring homework with for the afternoon visits.
- You will receive age-appropriate gifts at certain visits as a note of thanks.

Who can I talk to about the study?

- You can talk to Dr S Barnabas at FAMCRU if you have any questions or problems. His number is 021 938 5295 and after hours you can phone 084 641 0735.

What if I do not want to do this?

- It is your choice to take part or not. It is okay if you do not want to take part in this study. No one can force you and you do not have to tell anyone why you do not want to take part. You can decide not to take part even if your parent said yes to take part in the study. If you decide to take part, you can stop being in the study at any time without getting in trouble.

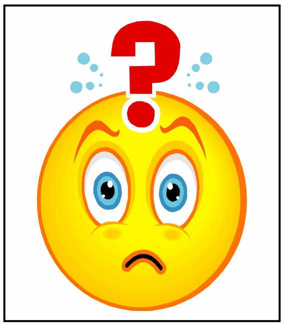

Do you understand this research study and are you willing to take part in it?

YES NO

Has the researcher answered all your questions?

YES NO

Do you understand that you can pull out of the study at any time?

YES NO

______________________ ______________________

Name and Surname of child **Signature of child**

______________ ______________ ______________

Place Date Time

Person obtaining informed consent and assent process:

I am satisfied that the child understands what the consent form is about and that his/her questions have been answered.

______________________ ______________________

Name and Surname **Signature**

______________ ______________ ______________

Place Date Time

References

1. UNAIDS. *Global report: report on the global AIDS epidemic*. 2010 [cited 2017 November]; Available from: http://files.unaids.org/en/media/unaids/contentassets/documents/unaidspublication/2010/20101123_globalreport_en[1].pdf.
2. WHO, *The global burden of disease: 2004 update*. 2008, Geneva: World Health Organization.
3. Kassebaum, N.J., et al., *A systematic analysis of global anemia burden from 1990 to 2010.* Blood, 2014. **123**(5): p. 615-24.
4. Zimmermann, M.B. and R.F. Hurrell, *Nutritional iron deficiency.* Lancet, 2007. **370**(9586): p. 511-20.
5. Horton, S. and J. Ross, *The economics of iron deficiency.* Food Policy, 2003. **28**(1): p. 51-75.
6. Rawat, R., et al., *Elevated iron stores are associated with HIV disease severity and mortality among postpartum women in Zimbabwe.* Public health nutrition, 2009. **12**(9): p. 1321-9.
7. Ellaurie, M., E.R. Burns, and A. Rubinstein, *Hematologic manifestations in pediatric HIV infection: severe anemia as a prognostic factor.* Journal of Pediatric Hematology/Oncology, 1990. **12**(4): p. 449-53.
8. Clark, T., et al., *Risk factors and cumulative incidence of anaemia among human immunodeficiency virus-infected children in Uganda.* Annals of tropical paediatrics, 2002. **22**(1): p. 11-7.
9. Belperio, P.S. and D.C. Rhew, *Prevalence and outcomes of anemia in individuals with human immunodeficiency virus: a systematic review of the literature.* The American journal of medicine, 2004. **116**(7): p. 27-43.
10. Adetifa, I. and U. Okomo, *Iron supplementation for reducing morbidity and mortality in children with HIV.* The Cochrane Library, 2009.
11. Eley, B.S., et al., *A prospective, cross-sectional study of anaemia and peripheral iron status in antiretroviral naive, HIV-1 infected children in Cape Town, South Africa.* BMC infectious diseases, 2002. **2**(1): p. 3.
12. James, E.B., et al., *Altered iron metabolism in children with human immunodeficiency virus disease.* Pediatric hematology and oncology, 2009. **26**(2): p. 69-84.
13. Weiss, G. and L.T. Goodnough, *Anemia of chronic disease.* New England Journal of Medicine, 2005. **352**(10): p. 1011-23.
14. Drakesmith, H. and A.M. Prentice, *Hepcidin and the iron-infection axis.* Science, 2012. **338**(6108): p. 768-72.
15. Nasi, M., et al., *Persistent inflammation in HIV infection: established concepts, new perspectives.* Immunology letters, 2014. **161**(2): p. 184-8.
16. Armitage, A.E., et al., *Distinct patterns of hepcidin and iron regulation during HIV-1, HBV, and HCV infections.* Proceedings of the National Academy of Sciences, 2014. **111**(33): p. 12187-92.
17. O'brien, M.E., et al., *Anemia is an independent predictor of mortality and immunologic progression of disease among women with HIV in Tanzania.* Journal of Acquired Immune Deficiency Syndromes, 2005. **40**(2): p. 219-25.
18. Savarino, A., G.P. Pescarmona, and J.R. Boelaert, *Iron metabolism and HIV infection: reciprocal interactions with potentially harmful consequences?* Cell biochemistry and function, 1999. **17**(4): p. 279-87.
19. Wisaksana, R., et al., *Inverse relationship of serum hepcidin levels with CD4 cell counts in HIV-infected patients selected from an Indonesian prospective cohort study.* PLoS One, 2013. **8**(11): p. e79904.
20. Al-Khafaji, B., S. Kralovic, and R.D. Smith, *Increased hepatic iron in the acquired immunodeficiency syndrome: an autopsy study.* Modern pathology: an official journal of the United States and Canadian Academy of Pathology, Inc, 1997. **10**(5): p. 474-80.
21. De Monyé, C., et al., *Bone marrow macrophage iron grade and survival of HIV-seropositive patients.* Aids, 1999. **13**(3): p. 375-80.
22. Minchella, P., et al., *Elevated hepcidin at HIV diagnosis is associated with incident tuberculosis in a retrospective cohort study.* The international journal of tuberculosis and lung disease, 2014. **18**(11): p. 1337-9.
23. Calis, J.C., et al., *HIV-associated anemia in children: a systematic review from a global perspective.* Aids, 2008. **22**(10): p. 1099-112.
24. Kruger, H.S., et al., *Positive association between dietary iron intake and iron status in HIV-infected children in Johannesburg, South Africa.* Nutrition research, 2013. **33**(1): p. 50-8.
25. Cercamondi, C.I., et al., *Afebrile Plasmodium falciparum parasitemia decreases absorption of fortification iron but does not affect systemic iron utilization: a double stable-isotope study in young Beninese women.* The American journal of clinical nutrition, 2010. **92**(6): p. 1385-92.
26. Zimmermann, M.B. and R.F. Hurrell, *Nutritional iron deficiency.* The Lancet, 2007. **370**(9586): p. 511-20.
27. Moore, R.D., J.C. Keruly, and R.E. Chaisson, *Anemia and survival in HIV infection.* Journal of Acquired Immune Deficiency Syndromes, 1998. **19**(1): p. 29-33.

28. Sullivan, P.S., et al., *Epidemiology of anemia in human immunodeficiency virus (HIV)-infected persons: results from the multistate adult and adolescent spectrum of HIV disease surveillance project.* Blood, 1998. **91**(1): p. 301-8.
29. Boelaert, J., G. Weinberg, and E. Weinberg, *Altered iron metabolism in HIV infection: mechanisms, possible consequences, and proposals for management.* Infectious agents and disease, 1996. **5**(1): p. 36-46.
30. McDermid, J.M., et al., *Elevated iron status strongly predicts mortality in West African adults with HIV infection.* Journal of Acquired Immune Deficiency Syndromes, 2007. **46**(4): p. 498-507.
31. Kupka, R., et al., *Iron status is an important cause of anemia in HIV-infected Tanzanian women but is not related to accelerated HIV disease progression.* The Journal of nutrition, 2007. **137**(10): p. 2317-23.
32. Schreck, R., P. Rieber, and P.A. Baeuerle, *Reactive oxygen intermediates as apparently widely used messengers in the activation of the NF-kappa B transcription factor and HIV-1.* The EMBO journal, 1991. **10**(8): p. 2247.
33. Gordeuk, V.R., et al., *Iron status and the outcome of HIV infection: an overview.* Journal of Clinical virology, 2001. **20**(3): p. 111-5.
34. Weinberg, G., J. Boelaert, and E. Weinberg, *Iron and HIV infection.* Micronutrients and HIV infection, 2002. **200**: p. 135-57.
35. Esan, M.O., et al., *Iron supplementation in HIV-infected Malawian children with anemia: a double-blind, randomized, controlled trial.* Clinical infectious diseases, 2013. **57**(11): p. 1626-34.
36. Olsen, A., et al., *Low-dose iron supplementation does not increase HIV-1 load.* Journal of Acquired Immune Deficiency Syndromes, 2004. **36**(1): p. 637-8.
37. Semba, R.D., et al., *Effect of micronutrients and iron supplementation on hemoglobin, iron status, and plasma hepatitis C and HIV RNA levels in female injection drug users: a controlled clinical trial.* Journal of Acquired Immune Deficiency Syndromes, 2007. **45**(3): p. 298-303.
38. Zimmermann, M.B., et al., *The effects of iron fortification on the gut microbiota in African children: a randomized controlled trial in Cote d'Ivoire.* The American journal of clinical nutrition, 2010. **92**(6): p. 1406-15.
39. Jaeggi, T., et al., *Iron fortification adversely affects the gut microbiome, increases pathogen abundance and induces intestinal inflammation in Kenyan infants.* Gut, 2014: p. gutjnl-2014-307720.
40. Klatt, N.R., N.T. Funderburg, and J.M. Brenchley, *Microbial translocation, immune activation, and HIV disease.* Trends in microbiology, 2013. **21**(1): p. 6-13.
41. Hunt, P.W., et al., *Gut epithelial barrier dysfunction and innate immune activation predict mortality in treated HIV infection.* The Journal of infectious diseases, 2014. **210**(8): p. 1228-38.
42. National Department of Health in South Africa. *Guidelines for the management of HIV-infected children*. 2010 [cited 2017 November]; Available from: https://www.google.ch/url?sa=t&rct=j&q=&esrc=s&source=web&cd=1&ved=0ahUKEwjjoZOcta_XAhVDLhoKHX6hAKgQFggyMAA&url=http%3A%2F%2Ffamilymedicine.ukzn.ac.za%2FLibraries%2FGuidelines_Protocols%2F2010_Paediatric_Guidelines.sflb.ashx&usg=AOvVaw2yqhX6LB9Ug2RcTn-LiA8D.
43. Brentlinger, P.E., et al., *Practical Management of HIV-Associated Anemia in Resource-Limited Settings: Prospective Observational Evaluation of a New Mozambican Guideline.* AIDS research and human retroviruses, 2016. **32**(1): p. 12-25.
44. Righetti, A.A., et al., *Etiology of anemia among infants, school-aged children, and young non-pregnant women in different settings of south-central Côte d'Ivoire.* The American journal of tropical medicine and hygiene, 2012. **87**(3): p. 425-34.
45. Zimmermann, M.B., et al., *Serum transferrin receptor and zinc protoporphyrin as indicators of iron status in African children.* American Journal of Clinical Nutrition, 2005. **81**(3): p. 615-23.
46. WHO, *Assessing the iron status of populations: report of a Joint World Health Organization/Centers for Disease Control and Prevention Technical Consultation on the Assessment of Iron Status at the Population Level, Geneva, Switzerland, 6-8 April 2004*, in *Assessing the iron status of populations: report of a Joint World Health Organization/Centers for Disease Control and Prevention Technical Consultation on the Assessment of Iron Status at the Population Level, Geneva, Switzerland, 6-8 April 2004*. 2005.
47. Fomon, S.J., et al., *Absorption and loss of iron in toddlers are highly correlated.* The Journal of nutrition, 2005. **135**(4): p. 771-7.
48. Green, R., et al., *Body iron excretion in man: a collaborative study.* The American journal of medicine, 1968. **45**(3): p. 336-53.
49. Hunt, J.R., C.A. Zito, and L.K. Johnson, *Body iron excretion by healthy men and women.* The American journal of clinical nutrition, 2009. **89**(6): p. 1792-8.
50. Troesch, B., et al., *Optimization of a phytase-containing micronutrient powder with low amounts of highly bioavailable iron for in-home fortification of complementary foods.* American journal of clinical nutrition 2009. **89**(2): p. 539-44.

51. Stoltzfus, R.J. and M.L. Dreyfuss, *Guidelines for the use of iron supplements to prevent and treat iron deficiency anemia*. Vol. 2. 1998: Ilsi Press Washington, DC.
52. Venter, C., U. MacIntyre, and H. Vorster, *The development and testing of a food portion photograph book for use in an African population.* Journal of Human Nutrition and Dietetics, 2000. **13**(3): p. 205-218.
53. Cercamondi, C.I., et al., *Iron bioavailability from a lipid-based complementary food fortificant mixed with millet porridge can be optimized by adding phytase and ascorbic acid but not by using a mixture of ferrous sulfate and sodium iron EDTA.* The Journal of nutrition, 2013. **143**(8): p. 1233-9.
54. Brown, E., et al., *Red cell, plasma, and blood volume in healthy women measured by radiochromium cell-labeling and hematocrit.* Journal of Clinical Investigation, 1962. **41**(12): p. 2182.
55. Linderkamp, O., et al., *Estimation and prediction of blood volume in infants and children.* European journal of pediatrics, 1977. **125**(4): p. 227-34.
56. Hallberg, L., L. Hulthen, and L. Garby, *Iron stores in man in relation to diet and iron requirements.* European journal of clinical nutrition, 1998. **52**(9): p. 623-31.
57. WHO, *Iron deficiency anaemia: assessment, prevention and control: a guide for programme managers.* 2001.
58. Erhardt, J.G., et al., *Combined measurement of ferritin, soluble transferrin receptor, retinol binding protein, and C-reactive protein by an inexpensive, sensitive, and simple sandwich enzyme-linked immunosorbent assay technique.* Journal of nutrition, 2004. **134**(11): p. 3127-32.
59. Walmsley, T., P. George, and R. Fowler, *Colorimetric measurement of iron in plasma samples anticoagulated with EDTA.* Journal of clinical pathology, 1992. **45**(2): p. 151-4.
60. Zimmermann, M.B., et al., *Iron metabolism in heterozygotes for hemoglobin E (HbE), α-thalassemia 1, or β-thalassemia and in compound heterozygotes for HbE/β-thalassemia.* The American journal of clinical nutrition, 2008. **88**(4): p. 1026-31.
61. Hotz, K., P.-A. Krayenbuehl, and T. Walczyk, *Mobilization of storage iron is reflected in the iron isotopic composition of blood in humans.* Journal of Biological Inorganic Chemistry, 2012. **17**(2): p. 301-9.
62. Turnlund, J.R., W.R. Keyes, and G.L. Peiffer, *Isotope ratios of molybdenum determined by thermal ionization mass spectrometry for stable isotope studies of molybdenum metabolism in humans.* Analytical chemistry, 1993. **65**(13): p. 1717-22.
63. Rios, E., et al., *The absorption of iron as supplements in infant cereal and infant formulas.* Pediatrics, 1975. **55**(5): p. 686-93.
64. Vandeputte, D., et al., *Quantitative microbiome profiling links gut community variation to microbial load.* Nature, 2017. **551**(7681).
65. International Atomic Energy Agency, *Assessment of iron bioavailability in humans using stable iron isotope techniques* IAEA human health series, 2012. **21**.
66. Paganini, D., et al., *Consumption of galacto-oligosaccharides increases iron absorption from a micronutrient powder containing ferrous fumarate and sodium iron EDTA: a stable-isotope study in Kenyan infants.* The American Journal of Clinical Nutrition, 2017: p. 1020-31.
67. Davidsson, L., et al., *Iron bioavailability in infants from an infant cereal fortified with ferric pyrophosphate or ferrous fumarate.* The American journal of clinical nutrition, 2000. **71**(6): p. 1597-602.
68. Davidsson, L., et al., *Influence of ascorbic acid on iron absorption from an iron-fortified, chocolate-flavored milk drink in Jamaican children.* American Journal of Clinical Nutrition, 1998. **67**(5): p. 873-7.
69. Cercamondi, C.I., et al., *Total iron absorption by young women from iron-biofortified pearl millet composite meals is double that from regular millet meals but less than that from post-harvest iron-fortified millet meals.* The Journal of nutrition, 2013. **143**(9): p. 1376-82.
70. Petry, N., et al., *Stable iron isotope studies in Rwandese women indicate that the common bean has limited potential as a vehicle for iron biofortification.* The Journal of nutrition, 2012. **142**(3): p. 492-7.
71. Cercamondi, C.I., et al., *Sodium iron EDTA and ascorbic acid, but not polyphenol oxidase treatment, counteract the strong inhibitory effect of polyphenols from brown sorghum on the absorption of fortification iron in young women.* British Journal of Nutrition, 2014. **111**(3): p. 481-9.

Proposal to the ETH Zurich Ethics Commission

A. General information

Project title:

Improving efficacy and safety of oral iron supplementation in HIV-infected children by providing prebiotic galacto-oligosaccharides as adjunct treatment: A randomized controlled trial

Principal Investigator(s):

Name	Title	Group / chair / institute	University
Jeannine Baumgartner	Dr.	Laboratory of Human Nutrition; Institute of Food, Nutrition and Health	ETH Zurich
Shaun Barnabas	Dr. MD	FAM-CRU, Department of Pediatrics and Child Health; Department of Pediatrics and Child Health; Faculty of Medicine and Health Sciences	Stellenbosch University

Involved researchers:

Name	Title	Group / chair / institute	University
Michael Zimmermann	Prof. MD	Laboratory of Human Nutrition; Institute of Food, Nutrition and Health	ETH Zurich
Renée Blaauw	Prof. PhD	Human Nutrition Division; Faculty of Medicine and Health Sciences	Stellenbosch University
Mark Cotton	Prof. MD	FAM-CRU, Department of Pediatrics and Child Health; Department of Pediatrics and Child Health; Faculty of Medicine and Health Sciences	Stellenbosch University

Source(s) of funding:

Thrasher Research Fund

Number of participants:

Minimum: 84

Maximum: 100

Study duration:

Beginning: September 2021

End: April 2022

Project type:

☒ Research project

☐ Bachelor/Master/Doctoral thesis

B. Scientific background

The study involves:

☐ Survey (online) ☐ Smartphone based data collection ☐ Interviews / group discussions

☐ Physical exercises / other activities ☒ Clinical trial abroad

1. Abstract

Background: In 2018, an estimated 260’000 South African children below 15 years of age were living with HIV. Especially in low-resource settings, these children are also at risk of developing iron deficiency (ID) and ID anaemia (IDA) due to poor quality diets and/or poor iron absorption. We hypothesize that this may be due to HIV infection-related inflammation reducing iron absorption by upregulating the iron-regulating hormone hepcidin. Recent studies propose that loss of gut mucosal integrity and gut microbiota dysbiosis are mechanisms contributing to chronic inflammation and increased morbidity in HIV disease.

Previous stable iron isotope studies from the ETH Laboratory of Human Nutrition showed that the consumption of prebiotic galacto-oligosaccharides (GOS) together with supplemental doses of iron can increase iron absorption, and mitigate gut inflammation induced by supplemental iron.

Thus, we hypothesize that providing GOS as adjunct treatment to oral iron supplementation will improve efficacy (iron absorption and iron status), reduce systemic and gut inflammation, improve mucosal integrity, and mitigated iron-induced alterations in the gut microbiome, adverse events and gastrointestinal side-effects in virally suppressed HIV-infected children.

Objectives: To determine the effect of prebiotic galacto-oligosaccharides (GOS) as adjunct treatment to 12 weeks of oral iron supplementation on: 1) iron status measured by conventional iron status biomarkers, 2) fractional absorption of iron (fraction of total body iron per day, measured as K_{abs}, the slope of ^{57}Fe isotopic dilution) and mean total amount of iron absorbed each day (mg Fe/day, calculated as K_{abs} x mean total body iron), 3) systemic and gut inflammation, as well as gut mucosal integrity, 4) gut microbiome composition, and 5) adverse effects and gastrointestinal side-effects, in virally suppressed HIV-infected children with anaemia and/or depleted iron stores.

Design: The participants of this randomized, double-blind, placebo-controlled 12-week trial will be recruited from a cohort of HIV-infected (n=90) South African children who previously (>14 month ago) received 12 mg isotopically labeled iron (^{57}Fe) as part of a stable iron isotope study, and who therefore have uniformly labelled body iron with ^{57}Fe. Additional subjects will be recruited from previous research cohorts of the Family Centre for Research with Ubuntu (FAM-CRU) at the Tygerberg Hospital in Cape Town and by their community outreach team. For this RCT, we will enroll 100 virally suppressed (viral load <50 copies per mL, measured as part of routine care) HIV-infected micro-/normocytic anaemic (haemoglobin <11.5 g/dL / 12 g/dL [children 8–11 / 12–15 years] and mean corpuscular volume [MCV] ≤91.5 fL) and/or iron deficient (ferritin <30 µg/L or soluble transferrin receptor [sTfR] >8.3 mg/L) children (10–15 years of age). Subjects will be randomly allocated to receive: 1) 50 mg oral iron as ferrous fumarate (FeFum) with 7.5 g of GOS or 2) 50 mg oral iron as FeFum with placebo (7.5 g of maltodextrin) daily for a period of 12 weeks. At baseline, 6 weeks and 12 weeks we will collect venous blood and faecal samples. Supplement compliance, adverse events and gastrointestinal and respiratory side-effects will be recorded daily by the caregiver using a compliance/morbidity diary, as well as by fieldworkers during monthly calls. In blood, we will perform a full blood count, measure biomarkers of iron status, hepcidin, systemic inflammation and gut mucosal integrity, viral loads and CD4+ T cells. We will further determine fractional

iron absorbed during the supplementation period by determining the isotopic composition of whole blood at all three time points and calculating the rate of decrease in the ^{57}Fe tracer concentration (measured as k_{abs}, the slope of ^{57}Fe enrichment). The mean total amount of iron absorbed and lost each day can then be calculated individually for each child. In fecal samples, we will measure gut microbiome composition, faecal pH, markers of inflammation (calprotectin and myeloperoxidase), as well as iron loss by measuring faecal isotopic composition.

Significance and overall goal: This study will form part of our current research efforts aimed at providing optimized recommendations on the treatment of ID and IDA in HIV-infected children in South Africa in order to improve their health, well-being, cognitive development and school performance, and therefore their long-term prognosis and quality of life. Furthermore, for the first time, we will apply the principle of long-term isotope dilution in assessing the efficacy of an oral iron intervention in children with HIV infection.

2. Project

2.1 Background

Iron deficiency anaemia is a common co-morbidity of HIV infection

In Sub-Saharan Africa, HIV is a major cause of mortality and morbidity among children. In 2018, an estimated 7.5 million South Africans lived with HIV, including ~260'000 children below 15 years of age (1). Worldwide, more individuals have iron deficiency (ID) than any other nutritional problem (2), and similar to HIV, the highest burden of iron deficiency anaemia (IDA) is in Sub-Saharan Africa (3). IDA in childhood can impair growth and cognition, reduce school performance, and impair immunity (4). Anaemia is an independent predictor of disease progression and mortality in HIV-infected children (5, 6), and is common before antiretroviral therapy (ART) is initiated. Although ART may reduce the prevalence of anaemia, many patients fail to resolve or develop anaemia after ART initiation (7). Observational studies show that prevalence rates of ID and IDA are often much higher in HIV-infected than uninfected children (8). The dual burden of anaemia and HIV in Sub-Saharan Africa has substantial health and economic costs (9). However, the relationship between these two often-overlapping disorders is complex and poorly understood (10).

Iron metabolism is altered by inflammation during chronic HIV infection

The pathophysiology of HIV-associated anaemia includes decreased dietary iron absorption, increased iron turnover, increased iron sequestration in the reticuloendothelial system and ineffective erythropoiesis (11). During infections, iron metabolism is carefully regulated: Iron is tightly bound in the intra- and extracellular compartments to reduce oxidative damage and limit its availability to potential pathogens (12). This effect is mediated by inflammation and is protective during acute infections. However, during chronic infections, inflammation has detrimental effects on iron status and haemoglobin (Hb). Chronic immune activation and inflammation are characteristic of both untreated and treated HIV infection (13, 14). During HIV infection, increases in interleukin (IL)-6, IL-22, and type I interferon (IFN) stimulate hepatic synthesis of hepcidin, which mediates redistribution of iron within the body (14), a process that becomes more pronounced with HIV disease progression (15, 16). Upregulated hepcidin inactivates ferroportin, which decreases intestinal iron absorption and increases the sequestration of iron in macrophages (12), limiting erythropoiesis (14, 17) and causing anaemia (18, 19). In adults, hepcidin remains significantly up regulated (roughly twofold) during chronic HIV infection, in concert with other inflammatory proteins (14). Greater inflammation, higher hepcidin levels, and impairments in iron metabolism predict a more rapid progression

of HIV disease (20). Even sub-clinical inflammation associated with protracted infections can increase hepcidin and sharply reduce iron absorption (21), thus efficacy of iron fortification and supplementation is likely compromised in HIV-infected children.

Risks of iron supplementation in anaemic HIV-infected individuals are unclear

ID and IDA in children may impair immune function (4). On the other hand, it remains unclear whether iron supplementation affects disease prognosis, related co-morbidities, and mortality in individuals with HIV (22). Two iron intervention trials in HIV-infected adults have reported reductions in anaemia without an increase in viral load or any other evidence of harm (23, 24). However, these studies were conducted in the era before access to ART became optimal. Some recent studies have suggested that oral iron supplements and fortificants alter the gut microbiota, and may increase gut inflammation in African children (25, 26). This maybe particularly detrimental in HIV-infected children, who may have gut immune activation, enteropathy and adverse shifts in the gut microbiome (27, 28). Because of concerns around safety and efficacy, there is no international consensus about iron supplementation for HIV-infected children. According to current South African standard treatment guidelines, HIV-infected and uninfected children >5 years of age with IDA should be treated with 3–6 months of oral iron supplementation in the form of ferrous sulfate ($FeSO_4$) or ferrous fumarate (FeFum), with haemoglobin (Hb) response monitoring at monthly intervals (29). However, routine iron supplementation is currently not recommended for HIV-infected children in most Sub-Saharan African countries (30). To our knowledge, there has been only one randomized controlled trial of iron supplementation, combined with multivitamins, in anaemic HIV-infected children. Iron supplementation improved Hb and immunity but also increased the frequency of malaria (22). Thus, current clinical practice is based on limited evidence and expert opinion (31), and whether iron supplementation and/or fortification is effective and safe in HIV infected children remains unclear.

The potential of prebiotic galacto-oligosaccharides in improving efficacy and safety of supplemental iron

Previous iron isotope trials in Kenyan infants and Swiss women performed by the Laboratory of Human Nutrition at ETH Zürich showed that prebiotic fibres, specifically galacto-oligosaccharides (GOS), enhance iron bioavailability (32) (33), and may mitigate gastrointestinal (GI) side effects caused by unabsorbed iron (34). Prebiotic fibres, typically oligosaccharides, such as fructo-oligosaccharides (FOS) and GOS, selectively enhance growth of beneficial colonic bacteria (35). The prebiotic GOS consists of glucose- and galactose-based di- and oligosaccharides of varying structure, and is not digested by enzymes in the human digestive tract. GOS reach the colon almost intact, where they are available for fermentation by commensal bacteria (36). This colonic fermentation produces short chain fatty acids (SCFA) that contribute to lower luminal pH, which may increase iron dissolution and absorption. In a single-blind, randomized trial, 50 infants (96% were anaemic) aged 6–14 months consumed maize porridge fortified with a multiple micronutrient powder (MNP) containing 5 mg iron (as FeFum and sodium iron ethylendiaminetetraacetate [NaFeEDTA]), with or without the addition of 7.5 g GOS, for 3 weeks (33). After the intervention period, infants were fed maize porridge and MNP test meals containing 5 mg isotopically labelled iron as ^{57}FeFum + Na^{58}FeEDTA or ferrous sulfate ($^{54}FeSO_4$), with or without GOS, respectively, on two consecutive days. Fractional iron absorption (FIA) was measured as the erythrocyte incorporation of the stable iron isotopes. The addition of GOS increased iron absorption from FeFum+NaFeEDTA by 62% (median FIA of 18.8% with vs. 11.6% without GOS), but only non-significantly increased iron absorption from $FeSO_4$ by 26% (median FIA of 25.5% with vs. 20.3% without GOS). The consumption of GOS further increased

Bifidobacterium and maintained higher amounts of *Lactobacillus/Pediococcus/Leuconostoc*, which correlated negatively with faecal pH and positively with iron absorption (33). In a controlled, prospective crossover study in iron deficient Swiss women (n=34) aged 18–34 years, the effect of 15 g GOS on iron absorption (doses of 14 mg) from FeFum or $FeSO_4$ was determined in test meals or water, before and after daily consumption of GOS for four weeks (32). At baseline, GOS significantly increased FIA from FeFum in water by 61% and in a test meal by 28%. After four weeks of GOS consumption, the effect of GOS on FIA from FeFum in the test meal persisted (+28%), with no further enhancement after chronic GOS exposure. The FIA from $FeSO_4$ in a test meal increased non-significantly from baseline to endpoint (after four-week consumption of GOS) by ~17%. In a more recent study, the ETH Laboratory of Human Nutrition determined whether the enhancing effect of GOS on FeFum (providing 14 mg of elemental iron) is dose-dependent, and showed that 7 g of GOS provided with FeFum significantly increased iron absorption, whereas 3.5 mg of GOS did not (37).

Recent studies propose that loss of gut mucosal integrity and gut microbiota dysbiosis are mechanisms contributing to chronic inflammation and increased morbidity in HIV disease (38). Thus, providing GOS as adjunct treatment to oral iron supplementation may not only improve efficacy, but also mitigate iron-induced alterations in the microbiome, as well as related gastrointestinal side-effects in virally suppressed HIV-infected children.

Assessing the efficacy of iron interventions during chronic infection, such as HIV, using conventional iron biomarkers is notoriously difficult

A major barrier to judging the efficacy of iron interventions in HIV-infected individuals is the limitations of conventional iron biomarkers in the face of chronic infection. The accurate assessment of iron status and longitudinal iron balance in HIV-infected populations are currently not possible because the commonly used blood indices of iron status (plasma ferritin [PF], soluble transferrin receptor [sTfR] and plasma iron [PFe]) are confounded by inflammation (11, 39, 40). Hb has low sensitivity and specificity in detecting ID and relatively large amounts of iron may be required to produce a measurable increase in Hb (41). Hepcidin can help interpret the interplay between anaemia, iron status and inflammation (12), but its levels may be increased by infections during IDA. Thus, there is no reliable method to evaluate the potential efficacy of iron fortification and supplementation during chronic infections, such as HIV, and there is no internationally agreed methodology to define iron status in such groups (42). This is a major challenge in global efforts to control anaemia in HIV infected populations.

Studies using isotopic dilution of whole body iron labeled with stable, non-radioactive isotopes of iron (^{58}Fe, ^{57}Fe, ^{54}Fe) could directly quantify iron absorption from interventions completely free of bias and confounding by infection or inflammation. This method could offer an accurate and quantitative measure of iron absorption and balance from iron interventions (43). After equilibration of an isotopic label in the body, the dilution of the label is solely dependent on the amount of unlabeled iron entering the body. Equilibration in adults takes 1 year, while in children, isotopic exchange with total body iron pools is complete in about 6-8 months (44). A basis for the isotopic dilution method is the slow turnover of the erythrocyte-iron compartment, modulated mainly by the rate of erythrocyte renewal. After equilibration in the body of a stable iron isotope (*tracer*), dilution of the *tracer* in erythrocytes is exclusively dependent upon the amount of iron with natural isotopic composition (*tracee*) entering the body iron pool where a mixture of *tracee* and *tracer* is present. In other words, assuming that all the iron in the body has a homogeneous iron isotopic composition, the amount of absorbed dietary and supplemental iron entering the body at

steady state can be assessed quantitatively by measuring isotopic enrichment of the *tracer* (45, 46). By measuring the dilution of labeled body iron, an extremely precise measure of iron absorption (k_{abs}) is obtained with an estimated capacity to detect a difference in iron absorption between the iron-fortified and placebo groups of as little as 12.5 µg Fe/kg body weight/day (Zimmermann M., ETH Zurich internal data). Furthermore, this method will allow a less burdensome (i.e., requiring fewer subject numbers per study), more accurate and cost-effective measurement of the efficacy and effectiveness of iron interventions. This could be a major advance over conventional study designs to monitor the effectiveness of iron interventions that require large numbers of subjects because of the inherent variability of blood biomarkers of iron status during chronic infections such as HIV. In this study, we will, for the first time, apply the principle of long-term isotope dilution in assessing the efficacy of iron supplementation, with and without GOS as adjunct treatment, in HIV-infected children.

2.2 Study objectives, hypotheses and outcomes

The aim of this study is to determine whether prebiotic GOS as adjunct treatment to iron supplementation can improve efficacy and safety/tolerability of oral iron supplementation in virally suppressed HIV-infected children.

The specific objectives are:

1) To determine the effect of prebiotic GOS as adjunct treatment to 12 weeks of oral iron supplementation in virally suppressed HIV-infected children with ID and/or anaemia on:

    a. iron status measured by conventional iron status biomarkers;

    b. fractional absorption of iron (fraction of total body iron per day, measured as K_{abs}, the slope of ^{57}Fe isotopic dilution) and mean total amount of iron absorbed each day (mg Fe/day, calculated as K_{abs} x mean total body iron);

    c. systemic and gut inflammation, as well as gut mucosal integrity;

    d. gut microbiome composition;

    e. fecal isotopic composition (measure of iron loss);

    f. HIV viral load and CD4+ T cells;

    g. adverse events, and gastro-intestinal and respiratory side effects.

2) To evaluate the stable iron isotope dilution method as a “reference” method to judge treatment efficacy.

We hypothesize that:

1) In virally suppressed HIV-infected children with ID and/or anaemia:

    a. Providing GOS as adjunct treatment to iron supplementation will improve iron status measured by conventional iron status indicators compared to iron supplementation without GOS.

    b. Providing GOS as adjunct treatment to iron supplementation will improve fractional absorption of iron (fraction of total body iron per day, measured as K_{abs}, the slope of ^{57}Fe

isotopic dilution) and mean total amount of iron absorbed each day (mg Fe/day, calculated as K_{abs}x mean total body iron) compared to iron supplementation without GOS.

c. Providing GOS as adjunct treatment to iron supplementation will reduce systemic and gut inflammation and increase gut mucosal integrity compared to iron supplementation without GOS.

d. Providing GOS as adjunct treatment to iron supplementation will mitigate alterations in gut microbiome composition induced by iron supplementation without GOS.

e. Providing GOS as adjunct treatment to iron supplementation will reduce iron loss due to occult blood loss.

f. Providing GOS as adjunct treatment to iron supplementation will mitigate a potential increase in HIV viral load induced by iron supplementation without GOS.

g. Providing GOS as adjunct treatment to iron supplementation will mitigate adverse events and gastro-intestinal and respiratory side effects induced by iron supplementation without GOS.

2) In HIV-infected children, the stable iron isotope dilution method will be a more sensitive and accurate method to determine the efficacy of iron interventions than measuring changes in conventional iron status biomarkers.

Co-primary outcomes

The co-primary outcomes of this study are related to the efficacy of oral iron supplementation with and without GOS as adjunct treatment, namely iron status measured using conventional iron status biomarkers (PF, sTfR, transferrin saturation [TSAT], Hb), as well as (in a sub-sample) fractional absorption of iron (fraction of total body iron per day, measured as K_{abs}, the slope of ^{57}Fe isotopic dilution) and mean total amount of iron absorbed (mg Fe/day, calculated as K_{abs} x mean total body iron) and lost each day. These outcome measures will be assessed at baseline, midpoint (6 weeks of supplementation) and endpoint (12 weeks of supplementation).

Secondary outcomes

The secondary outcomes of this study are related to the safety/tolerability of oral iron supplementation with and without GOS as adjunct treatment, namely:

- Systemic inflammation (C-reactive protein [CRP], alpha-1-acid glycoprotein [AGP])
- Hepcidin
- Gut inflammation (faecal calprotecin and myeloperoxidase [MPO]) and gut mucosal integrity (plasma intestinal fatty acid binding protein [I-FABP])
- Gut microbiome composition (specifically *Bifidobacterium* spp and *Lactobacillus/Pediococcus /Leuconosstoc* spp.)
- Iron loss by measuring faecal isotopic composition
- HIV viral load and CD4+ T cell counts
- Adverse events and gastrointestinal and respiratory side effects

2.3 Project schedule

Project activity	Duration	Time
Ethical approvals obtained at both institutions, set-up	9 months	February – September 2021
Subject recruitment and screening	2 months	September & October 2021
12-week randomized controlled trial	4 months	September – December 2021
Sample and data analysis (main outcomes)	3 months	January 2022 – April 2022
Writing and submission of manuscripts	3 months	April – June 2022

2.4 Methods

2.4.1 Study site, design and subjects

This randomized, double-blind, placebo-controlled 12-week trial (RCT) will be conducted at the Family Centre for Research with Ubuntu (FAM-CRU) of the Department of Paediatrics and Child Health of the Stellenbosch University, which is located inside the Tygerberg Academic Hospital complex in Cape Town (Western Cape Province), and is adjacent to the Family Clinic for HIV. FAM-CRU has 1000m^2 research space for clinical trials and prospective cohort studies. It has over 100 full-time research staff including 15 nurses and 12 doctors. The Western Cape Province is a malaria free area. The subjects for this RCT will be recruited from a cohort of HIV-infected (n=90) South African children who previously (>14 month ago) received 12 mg isotopically labeled iron (Fe^{57}) as part of a stable iron isotope study ("A novel stable iron isotope method to define iron needs and improve iron nutrition in HIV+ and HIV- children"; EK 2018-N-40, S18/06/136 and M18/05/017), and who therefore have uniformly labelled body iron with Fe^{57}. Additional subjects will be recruited from previous research cohorts of FAM-CRU and by their community outreach team. For this RCT, we will enroll 100 virally suppressed (viral load ≥50 copies per mL; from routine care) HIV-infected children (10–15 years of age) with mild to moderate micro-/normocytic anaemia (Hb ≥8.0 and <11.5/12.0 g/dL plus mean corpuscular volume [MCV] ≤91.5 fL) and/or ID (ferritin <30 µg/L or sTfR >8.3 mg/L).

Subjects will be randomly allocated to receive: 1) 50 mg oral iron as ferrous fumarate (FeFum) with 7.5 g of GOS or 2) 50 mg oral iron as FeFum with placebo (7.5 g of maltodextrin) daily for a period of 12 weeks. Blood and fecal samples for the assessment of primary and secondary outcomes will be collected at baseline, 6 weeks and 12 weeks. Supplement compliance, adverse events and gastrointestinal and respiratory side-effects will be recorded daily by the caregiver using a compliance/morbidity diary, as well as by fieldworkers every three weeks during the intervention period.

2.3.2 Study procedures

An outline of the study is shown in **Figure 1.** An overview of the different procedures and assessments is shown in **Table 1.**

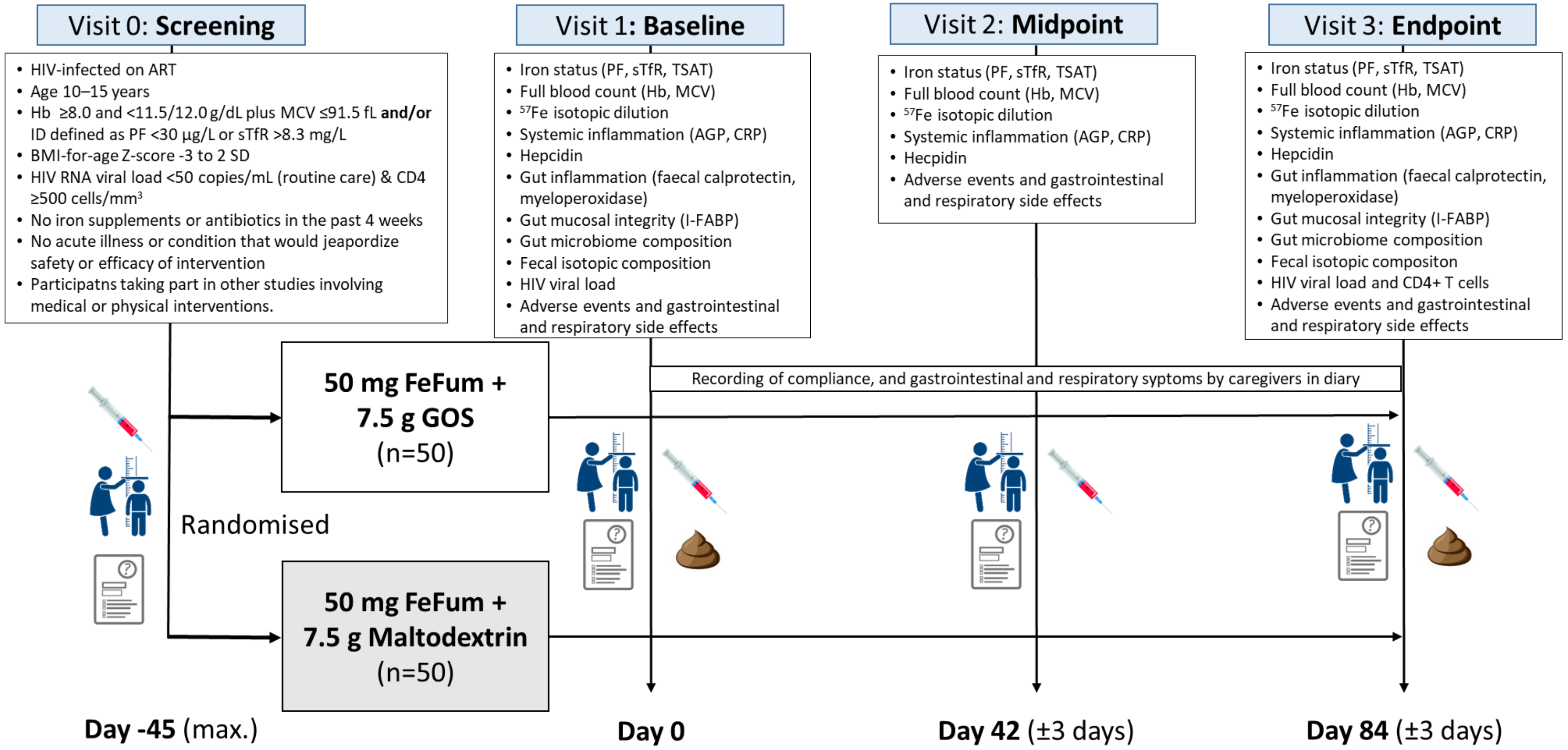

Figure 1: Study outline. HIV, human immunodeficiency virus; ART, antiretroviral therapy; Hb, haemoglobin; MCV, mean corpuscular volume; PF, plasma ferritin; BMI, body mass index; sTfR, soluble transferrin receptor; TSAT, transferrin saturation; AGP, alpha-1 glycoprotein; CRP, C-reactive protein; I-FABP, intestinal fatty acid binding protein.

Table 1: Plan of procedures and assessments

	Screening	Baseline	Midpoint	Endpoint
			12-week intervention period	
**Visit**	**0**	**1**	**2**	**3**
**Day**	**-45**	**0**	**42 (±3)**	**84 (±3)**
Informed consent	X			
Assignment to subject ID	X			
Checking in/exclusion criteria	X			
Socio-demographic information	X			
Health check/medical history	X	X	X	X
Randomisation to intervention	X			
Anthropometry (height/weight)	X	X	X	X
Venous blood sampling	X[a]	X[b]	X[c]	X[b]
Faecal sample collection		X		X
Distribution of supplements		X	X	X
Compliance monitoring			X	X
Adverse event / side effect monitoring[d]		X	X	X

[a]Iron status: Full blood count, plasma ferritin, soluble transferrin receptor; CD4+ T cell count.
[b]Iron status: Full blood count, plasma ferritin, soluble transferrin receptor, transferrin saturation; ^{57}Fe isotopic dilution; hepcidin; systemic inflammation (C-reactive protein, alpha-1 glycolic acid); gut mucosal integrity (intestinal fatty acid binding protein); HIV viral load.
[c]Iron status: Full blood count, plasma ferritin, soluble transferrin receptor, transferrin saturation; ^{57}Fe isotopic dilution; hepcidin; systemic inflammation (C-reactive protein, alpha-1 glycolic acid).
[d]Caregives will further be contacted by study team members to follow-up on adverse events / side effects 3 weeks after baseline (day 21) and 3 weeks after the mid-point (day 61).

Recruitment and screening (visit 0)

The caregivers of HIV-infected children (n=90) who participated in our previous stable iron isotope study ("A novel stable iron isotope method to define iron needs and improve iron nutrition in HIV+ and HIV- children"; EK 2018-N-40, S18/06/136 and M18/05/017), and who therefore have uniformly labelled body iron with ^{57}Fe, will be contacted by the FAM-CRU staff and invited to participate in the screening for this study. All participants and their caregivers have previously been informed about an additional study that will form part of the larger project, and have given permission to being contacted by FAM-CRU for participating in this study. Additionally, FAM-CRU staff will contact and invite caregivers of children who participated in previous research cohorts of FAM-CRU or new caregivers-child pairs recruited by the FAM-CRU community outreach team. All potentially interested participants will be sent/handed out the written study information sheet (available in English, Afrikaans and isiXhosa) before the screening visit and will therefore have time to think about it and discuss it with their child.

At the screening visit (visit 0), the caregiver will again be provided with written and oral information about the study and will be asked to sign the informed consent form (ICF) (Attachment 1). Assent will be obtained from the child (Attachment 2). After obtaining informed consent and assent, the child will be assigned a participant ID (will stay the same if enrolled into the study) and will be screened for eligibility. Screening will include the withdrawal of a venous blood sample (10 ml) for the performance of a full blood count (Hb and MCV) and the analysis of plasma ferritin, HIV RNA viral loads and CD4+ T cell counts. Anthropometric measurements (height and weight) will be taken and socio-demographic

information collected. Data on medical history will be collected and a general health check performed by the study nurses in order to identify subjects who are ineligible based on a chronic disease or acute illness).

Children who are eligible for the study will be enrolled into the study and contacted telephonically to schedule the dates for the study visits (visit 1–3).

Baseline, midpoint and endpoint visits

The baseline visit (visit 1) will take place a maximum 45 days after the screening visit. The midpoint visit (visit 2) will take place after 6 weeks of intervention (day 42 [± 3 days]), and the endpoint visit (visit 3) will take place after 12 weeks of intervention (day 84 [± 3 days]). During all three visits, anthropometric measurements will be taken, a health check will be performed, as well as compliance and adverse event and side effect monitoring sheets collected (see below for more detail). We will also record the following variables: 1) Tanner pubertal stage (specifically current menstrual pattern if post-menarcheal); 2) antibiotic exposures, tuberculosis treatment, antiretroviral therapy (specifically zidovudine and lamivudine exposure); and 3) hospital admissions (nosocomial pathogen exposure). A venous blood sample (10 ml) will be collected during all three visits for a full blood count and biochemical analysis of iron status (PF, sTfR, TSAT), ^{57}Fe isotopic dilution, systemic inflammation (AGP and CRP), hepcidin and gut mucosal integrity (I-FABP) (only visits 1 & 3). At baseline and endpoint, we will additionally collect a faecal sample for the analysis of gut inflammation (faecal calprotectin), faecal pH, gut microbiome composition and faecal isotopic composition. If the child is not able to provide a faecal sample while on site, the research team will carefully explain and demonstrate the collection procedure to the caregiver.

Intervention, randomization and blinding

Stratified by sex and age, children will be randomly assigned in a 1:1 ratio to one of two intervention groups (n=50/group): 1) Oral iron supplements (50 mg of elemental iron as FeFUM) with 7.5 g of GOS (FeFUM+GOS) or 2) oral iron supplements (50 mg) with 7.4 g of maltodextrin (FeFUM+placebo). Randomization will be done using the randomization sequence generated by the RANNOR function of the SAS software package version 9.4. by an experienced biostatistician. The two intervention groups will be assigned a code and colour facilitate implementation and monitoring.

Researchers, research staff and subjects will be blinded to the intervention. To blind data analysis, the colour codes will be re-coded to two random alpha- numerical codes by two members of the research team not directly involved with the analysis or initial interpretation of the data. This will be done immediately prior to data analysis and after all data have been collected. One written document containing the key to these new codes will be held in the office of an independent researcher at the Stellenbosch University and another copy will be held by a member of the data safety monitoring board (DSMB). The codes will be broken once data analyses have been completed.

Intervention products and delivery

Both intervention groups will receive daily oral iron supplements, providing 50 mg elemental iron in the form of FeFum (Rulofer N, Lohmapharm GmbH, Hannover, Germany), for an intervention period of 12

weeks. The dose and duration of intervention is aligned with the South African Standard Treatment Guidelines and Essential Medicines List (47). The children assigned to the FeFUM+GOS group will receive a daily dose of 7.5 g GOS, which will be provided in sachets containing 10.5 g powder (75% GOS; Vivinal GOS 75 Powder; FrieslandCampina) that has to be dissolved in ~200 ml of water before consumption. We chose this GOS dose based on our previous studies showing an enhancing effect of GOS on iron absorption at this dose (32, 33, 37), and on the basis of studies that reported a bifidogenic effect in infants and adults at a range of doses from 2.5 to >10 g/d (48). The children in the FeFum+Placebo group will receive 10.5 g of maltodextrin powder, which will have to be prepared the same way as the GOS powder and does look identical to the GOS powder and solution. The caregivers will be carefully instructed on how to prepare the GOS and placebo solutions, and that children should consume the iron tables together with the respective solutions. The labels on the intervention products will be identical but will contain the respective treatment code and colour.

Compliance, and adverse event and side effect monitoring

Data on compliance, adverse events and side effects will be collected by means of a weekly compliance and morbidity diary that the caregivers will be asked to complete every day after administering the intervention products to their child (Appendix 4). Each caregiver will be provided with a 6-week supply of intervention product at the baseline visit (visit 1) and another 6-week supply at the midpoint visit (visit 2). At the midpoint and endpoint visit, the compliance and morbidity dairies will be collected, as well as left-over product and empty sachets / supplement containers. In addition, FAM-CRU staff and fieldworkers will call the caregiver on a monthly basis to ask/remind on supplement compliance and record potential adverse events that have occurred in the previous month. Compliance to treatment will be assessed by counting of tablets according to the following formula: Compliance to treatment (%) = number of capsules actually taken from the last count /number of tablets should be taken at the same stage x 100% (49), as well as based on the recorded compliance on the compliance/morbidity diary that was completed by the caregiver. We will consider optimal compliance to be higher than 80%.

2.3.3 Data/biological sample collection and laboratory analysis

Anthropometry

Anthropometric measurements will be performed using standardised techniques [24]. Weight and height will be measured using a Micro 1023 electronic platform scale and stadiometer (Scalerite, Johannesburg, South Africa). Children will be measured barefoot in a single layer of clothing. When applicable, hair will be undone and hair accessories removed. Weight will be measured to the nearest 0.1 kg and height to the nearest 0.1 cm. Stunting will be defined as height-for-age Z-scores (HAZ) < -2 SD from the WHO reference median. Underweight and severe underweight will be defined as BAZ < -2 SD and < -3 SD from the WHO reference median, respectively [23]. Overweight will be defined as BAZ > 1 SD from the WHO reference median.

Iron status and inflammation indices

Venous blood samples will be drawn by an experienced paediatric phlebotomist or nurse into trace metal free lithium-heparin and EDTA coated vaccutainers. Full blood counts will be performed in EDTA whole blood using a Siemens Advia 2120i Haematology System at the National Health Laboratory Services (NHLS) at Tygerberg Hospital. Anemia for children 8-11 years old will be defined as Hb <11.5 g/dL, and for children 12 years old as Hb <12 g/dL (50). Macrocytosis will be defined as an MCV >91.5 fL. Aliquots of whole blood (2 x 0.5 ml) drawn into the lithium-heparin tubes will be frozen at -20°C until shipment to ETH Zurich in Switzerland for the analysis of ^{57}Fe isotopic dilution. Remaining blood will be processed within 1 hour after blood sampling. Plasma and erythrocytes will be separated by centrifugation and immediately aliquoted and stored at -20°C.

For screening of children with ID, PF and sTfR will be measured at Lancet Pathology Laboratories in Johannesburg, South Africa using the Roche Cobas system. sTfR values obtained using the Tina-quant Assay of the Roche Cobas system can be converted to Ramco equivalents using the following equation: Roche [mg/L] = 0.631 * Ramco [mg/L] + 0.299 (51). Thus, an sTfR cut-off of 5.54 mg/L will correspond to the defined screening cut-off of 8.3 mg/L, which is based on the Ramco assay that will be used for the baseline and endpoint iron status assessment.

Conventional biomarkers of iron status (PF, sTfR) and inflammation (AGP, CRP) will be analyzed in lithium-heparin plasma using a multiplex immunoassay technique (52). ID will be defined as unadjusted PF <30 µg/L or inflammation-adjusted PF <15 µg/L (50) and/or elevated sTfR >8.3 mg/L (52), and IDA as Hb <11.5 or 12 g/dL and PF <30 µg/L (50) and/or sTfR >8.3 mg/L (52). Expected CRP and AGP concentrations for healthy children are >5 mg/L and >1 g/L, respectively (52). Plasma hepcidin concentrations will be measured using ELISA (DRG Instruments GmbH, Marburg, Germany). Intestinal fatty acid binding protein (I-FABP), a plasma marker of intestinal integrity, will be measured using a commercially available ELISA kit (Hycult Biotech, Uden, The Netherlands). TSAT is the percentage of transferrin that has iron bound to it. It is the ratio plasma iron (PFe) and total iron binding capacity (TIBC) multiplied by 100. For PFe measurement, acid extraction of plasma with hydrochloric acid and trichloroacetic acid liberates the iron from transferrin and precipitates the protein. The acid reagent also contains thioglycolic acid, which reduces the iron to its ferrous oxidation state. Iron concentration is then determined by measuring the absorbance of the lilac color development. TIBC is a measure of iron-binding sites available on plasma transferrin. A solution of ferric chloride is added in sufficient quantity to the plasma sample to saturate the iron binding capacity of plasma. The excess unbound iron is removed and the iron concentration in the supernatant is measured colorimetrically. Iron concentration is determined by measuring the absorbance of the lilac color development (53). Viral loads will be measured at NHLS using the Roche COBAS AmpliPrep/TaqMan HIV-1 Test, v2, with an input volume of 1 mL allowing detection of viral loads from 20 copies per mL. CD4+ T cells (numbers and %) will be measured in whole blood using immunophenotyping by multicolour flow cytometry at the NHLS.

Gut microbiome and gut inflammation analysis

Faecal samples will be collected at baseline (visit 1) and endpoint (visit 3) on site or at home using a user-friendly faecal collection kit consisting of a lunchbox size cooler bag and two ice packs, a plastic container and lid that seal airtight once closed, and an Anaerocult A mini bag (Merck Millipore) to maintain anaerobiosis, along with illustrated sample collection instructions in the child and caregiver's

home language. A study nurse will unpack the kit and carefully explained the sampling process in a preceding study visit. Stool samples will be aliquotted on-site and stored at −70°C until shipment to ETH Zurich, Switzerland for analysis.

At ETH Zurich, faecal samples will be thawed at 4°C before further preparation for measurement of pH and calprotectin. For measurement of pH, 100 mg (±10%) of faeces will be added to 1 mL 36 nanopure water, vortexed for 30 sec and centrifuged for 3 min at 5000 rpm at 4°C; pH in the liquid phase will be measured using a digital pH meter (Metrohm, Zofingen, Switzerland). Faecal calprotectin, a gut inflammation marker, will be measured using the Calprest ELISA assay for stool, following the manufacturer's procedures (Eurospital, Trieste, Italy). Faecal myeloperoxidase (MPO), a marker of gut neutrophil activation (inflammation) will be measured using ELISA (manufacturer to be determine), following the manufacturer's procedures. To ensure the quality of the measurements, controls provided by the manufacturer will be analyzed together with the samples, standard curves will be regularly performed in each run and all samples are measured in duplicate. Targeted qPCR will be performed using specific primers for the bacterial subgroups most prevalent in the human gut and expected low-abundant pathogens (54, 55). Specifically, we will target *Bifidobacterium* ssp., *Lactobacillus/Pediococcus /Leuconostoc* spp. and *Bacteroides* spp. because they are major commensals with growth that is selectively enhanced in children by consumption of prebiotics (56). In addition to the targeted qPCR analysis, we may also perform untargeted microbiota analysis using 16S rDNA sequencing.

Measurement of iron absorption and losses with stable iron isotope (^{56}Fe)

In the sub-sample of children who previously (>14 month ago) received 12 mg isotopically labeled iron (^{57}Fe) as part of a stable iron isotope study, and who therefore have uniformly labelled body iron with ^{57}Fe, we will directly measure iron absorption and losses from the interventions by determining the isotopic composition of whole blood at baseline, midpoint and endpoint. All isotopically enriched blood samples will be mineralized in duplicate using an HNO_3/H_2O_2 mixture and microwave digestion followed by separation of the sample iron matrix by anion-exchange chromatography and a subsequent precipitation step with ammonium hydroxide (57). All isotopic analysis will be performed by inductively coupled plasma mass spectrometry (ICP-MS) using a high-resolution magnetic sector mass spectrometer (Neptune, Thermo-Finnigan, Germany) equipped with a multi-collector system for simultaneous ion beam detection at ETH Zurich (58).

The addition of iron of natural isotopic composition by iron absorption increases body iron, decreasing the concentration of ^{57}Fe tracer (i.e., the ratio ^{57}Fe/body iron) but does not alter the amount of ^{57}Fe (43). Conversely, loss of iron from the body results in parallel decreases in the amounts of both ^{57}Fe tracer and iron of natural isotopic composition without changing the concentration of ^{57}Fe tracer. The decrease in the concentration of ^{57}Fe tracer in the circulation from addition of iron of natural isotopic composition is proportional to the absorption of dietary or supplemental iron. This relationship is described by the slope of the natural logarithm of the ^{57}Fe concentration plotted over time (k_{abs}), measuring the fraction of total body iron absorbed per unit of time. Similarly, a decrease in the amount of ^{57}Fe tracer in the body with loss of ^{57}Fe and the parallel loss of body iron of natural isotopic composition is described by the slope of the natural logarithm of the ^{57}Fe amount plotted over time (k_{loss}), the fraction of total body iron lost per unit of time.

Mean iron absorption (Fe_{abs}) over the intervention period will be calculated as: $Fe_{abs} = -k_{abs} \times Fe_{total}$
Fe_{total} is the total body iron at the midpoint calculated from the linear regression of Fe_{total} against time individually for each observational period, and k_{abs}, the fraction of total body iron absorbed per unit of time, is the slope of the linear regression of the natural logarithm of the ^{57}Fe tracer concentration plotted against time. Mean total amount of iron absorbed each day (mg Fe/day) is calculated as k_{abs} x mean total body iron.

In addition to the estimation of iron losses determine using $k_{abs,}$ we will also estimate iron loss due to occult blood loss by measuring changes in isotopic composition in faecal samples from baseline to endpoint.

2.3.4 Quality assurance and control

Quality assurance of biological samples

To ensure quality of stored samples, the freezer at the study site is connected to a back-up power generator and a temperature log is completed daily by a laboratory technician. All samples (plasma, whole blood and faeces) will be shipped to Switzerland with World Courier using temperature-controlled transport to ensure that the samples remain frozen during shipment. During transport, World Courier will regularly control whether there is sufficient dry ice in the transport box and refill if necessary. Upon arrival in Switzerland, the samples will immediately be stored at −20°C at the ETH Zurich, Switzerland. The principal investigator (PI) and co-investigators located at ETH Zurich will be responsible for proper sample storage and further analysis of iron status and inflammation indices.

Quality assurance laboratory tests

All laboratory analyses will be done using controls/standards with known values and will be conducted by experienced personnel.

The measurement of PF, sTfR, CRP and AGP will be done by a technician who has developed the sophisticated method measuring all four parameters in a single aliquot of 100-200 µL (52). To ensure the quality of these measurements certified quality control samples from the CDC/Atlanta as well as Biorad Liquicheck controls are analyzed together with the samples.

The isotopic analysis in while blood will be controlled by using a special type of in-house controls, called spikes, which have a known ratio of the four iron forms. This allows quality assurance of the mass spectrometry used to measure the ratio in the blood samples. Technicians from ETH Zurich with extensive experience from numerous previous iron absorption studies will conduct the analysis.

The quality of the iron status, iron and gut inflammation analysis is ensured by performing standard curves routinely in each run; and external controls, provided by the manufacturer and are analyzed together with the samples and all samples are analyzed in duplicate. Analyses will be done by technicians from the ETH Zurich with extensive experience under close supervision of the PI and the Swiss co-investigators.

2.3.5 Statistics

Sample size calculations

Sample size was calculated based on an expected 25% difference in ferritin at endpoint between the children in the FeFUM group (50 µg/L) and in the FeFUM+GOS group (62.5 µg/L), and an SD of 20 µg/L (ferritin values and SD based on previously observed values in iron-replete HIV-positive children from the same cohort), which corresponds to a medium-to-large effect size of 0.625. Using an alpha of 95% and a power of 80%, the estimated sample size is 42 children per group. Considering a dropout rate of 20%, results in a final sample size of 50 children per group (n=100 in total).

The minimum sample size for the sub-sample of isotopically labelled children from our ongoing cohort, was calculated based on an observed K_{abs} of -0.00355 (SD = 0.00099) in Gambian toddlers who received 12 mg iron as FeFUM for 12 weeks, and an expected 35% decrease in K_{abs} with GOS to -0.004615 (effect size = 1.076) (43). Using an alpha of 95% and a power of 80%, the estimated sample size is 15 children per group. Considering a dropout rate of 20%, results in a final sample size of 18 children per group (n=36 in total).

Data management and statistical analysis

Data will be captured and managed using REDCap (Research Electronic Data Capture), programmed with quality-check parameters to minimize errors during data entry. Data will be backed up on an external hard drive routinely at the end of each day and after each set of data was entered.

Data will be analysed according to an intention-to-treat principle, and missing values will be imputed using multiple imputations. Data will be tested for normality by means of Q-Q plots, histograms, and Shapiro-Wilk tests. Normally distributed data will be expressed as means ± SD, and not normally distributed data (if normally distributed after log transformation) as geometric mean values (95% CI). Data still not normally distributed after log transformation will be presented as median (IQR). We will estimate intervention effects (β-coefficients [95% CIs]) for FeFUM+GOS versus FeFUM+Placebo on continuous primary and secondary outcome variables measured at endpoint using Analysis of Covariance (ANCOVA) and adjusting for respective baseline values and potential confounders (e.g. age and sex). Binary outcomes (e.g. anaemic vs. non-anaemic at the end of intervention) will be analysed using logistic regression models. Statistical analysis will be performed using IBM SPSS Statistics software (version 26), and the level of significance will be set at $P < 0.05$.

3. Ethical considerations

This study will be conducted in accordance with internationally accepted ethical standards and guidelines, including the International Conference on Harmonization for GCP, and the Declaration of Helsinki (version 2013). Both, the Ethics Committee of ETH Zurich in Switzerland, and the Health Research Ethics Committee of Stellenbosch University in South Africa, will approve the protocol and all procedures involving human study participants.

Detailed oral and written information explaining the study purposes, potential risks and benefits will be provided to the participants and their caregivers.

The participation includes the small risks of bruise, infection and/or phlebitis, and discomforts during having blood drawn from the child. These risks will be explained to the caregivers and children. Nonetheless, morbidity of the children will be closely followed-up by a fieldworker every three weeks. Furthermore, caregivers will record adverse events and symptoms daily in a weekly compliance/morbidity diary. Participants becoming severely anemic (Hb <8 g/dL) at any time during the study will receive standard of care treatment according to local guidelines, but will not be excluded from the study. Participants developing secondary infections at any time during the study will receive standard of care treatment and iron may be withheld for several days until the appropriate treatment is complete, but the participants will not be excluded.
The benefits of the study include the iron supplementation, which is likely to have a positive effect on the child's iron status. Furthermore, the child's health will be monitored closely throughout the study and sick children will be treated free of charge at the Tygerberg Children's Hospital. Caregivers will receive an allowance to compensate for the invested time and for transport costs (R150 – 300) per study visit, depending on the distance traveled. Furthermore, the investigators believe that this study will directly benefit the community from which the data is drawn since it will make recommendations about best practice within public sector clinics.

3.1 Participants

3.1.1 Exact number of participants

100 HIV+ children, 10–15 years old

3.1.2 Inclusion and exclusion criteria

Inclusion criteria

- Age 10–15 years at baseline;
- Mild to moderate micro- and normocytic anaemia defined as Hb ≥8.0 and <11.5 / 12 g/dL (children 8–11 / 12–15 years) plus MCV ≤91.5 fL **and/or** ID defined as ferritin <30 µg/L or sTfR >8.3 mg/L;
- Body-Mass-Index-for-age Z-scores (BAZ) −3 to 2 SD of reference population;
- HIV criteria: CD4 ≥500 cells/mm^3; HIV RNA viral load <50 copies/mL (measured as part of routine care);
- Willingness of caregiver to participate in the study;
- Caregiver speaks English, Afrikaans or isiXhosa;
- The informed consent form has been read and signed by the caregiver (or has been read out to the caregiver in case of illiteracy) plus assent needs to be obtained from the child;
- Residence in the study area for the period of the study.

Exclusion criteria

- Child received iron supplements or antibiotic treatment in the past 4 weeks;

- Acute illness or other conditions that in the opinion of the PI or co-researchers would jeopardize the safety or efficacy of intervention or would render the participant unable to comply with the protocol;
- Participants taking part in other studies involving medical or physical interventions.

3.2 Risks and countermeasures

Blood sampling

Blood withdrawal will be done by paediatric technicians or nurses with extensive experience with child venipuncture in the Tygerberg Children's Hospital, using sterile equipment and technique. The small risk of irritation, minor bruising, inflammation or phlebitis after venipuncture will be explained to the caregivers of the participating child. Since the children are under active surveillance, the venipuncture site will be checked at the next visit. The purpose of the blood withdrawals in the study will be clearly explained to the caregivers and children, as well as the methods through which these samples will be examined and stored. Results of blood analysis (Hb and PF) will be communicated and explained to the caregivers.

Severely anemic and severely underweight

During screening, Hb, PF and BMI-for-age z-scores will be assessed. Severely anemic (Hb <8 g/dL) or severely underweight (BMI-for-age z-scores <-3 SD) will not be included in the study and will be referred to the paediatricians at Tygerberg Children's Hospital for appropriate treatment free of charge.

Stool sampling

The collection of stool samples entails no risk for the child.

Safety aspect of interventions

No study drug will be administered in the study. The interventions consists of oral FeFum supplements and GOS. Iron supplementation may lead to side effects, such as stomach upset and pain, constipation, diarrhoea, nausea or vomiting, especially in the first two weeks of taking the supplements. An increased risk for infectious disease, as well as an increase in HIV viral loads from using iron supplements is not expected. Nonetheless, morbidity of the children will be closely monitored, and infectious diseases will be surveyed and treated according to the national guidelines.

Illness during study

Caregivers will be instructed to bring children that fall sick during the study to the nearest appropriate facility, where children will be examined, and to report us of the event. Furthermore, morbidity will be recorded by the caregiver using the weekly compliance/morbidity diary and by fieldworkers every three weeks during the intervention period. Participants becoming severely anemic (Hb <8 g/dL) at any time during the study will receive standard of care treatment according to local guidelines, but will not be excluded from the study. Participants developing secondary infections at any time during the study will receive standard of care treatment and iron may be withheld for several days until the appropriate treatment is complete, but the participants will not be excluded.

Data safety monitoring board (DSMB)

The primary responsibility of the DSMB will be to protect the ethical and safety interests of subjects enrolled into the trial. Hence, the DSMB will be established to review and monitor the safety data of the project and to provide independent advice on safe and ethical conduct of this research project involving human subjects. The DSMB will provide recommendations about stopping, continuing or modifying the trial based on continued review of the safety data during the study. The activity of the DSMB will be delineated in a charter that will define the membership, responsibilities and the scope and frequency of data reviews. The DSMB will operate on a conflict-free basis independently of the PI and the study team. The DSMB will comprise of four independent members of nutrition scientists and pediatricians (Prof. Dr. Gary Brittenham; Prof. Dr. Etienne Nel; Prof. Marius Smuts; Dr. Diego Moretti). All potential DSMB members have seen the protocol/outline before agreeing to join the committee. DSMB members should be independent and constructively critical of the ongoing project, but also supportive of aims and methods of the project. The DSMB shall meet (via teleconference) before the start of the intervention to discuss the protocol, the trial, analysis plan, future meetings, and to have the opportunity to clarify any project aspects with the PI. The PI or the DSMB may convene ad hoc meetings if safety concerns arise during the trial. After its assessment, the DSMB will recommend to the PI continuation, modification or termination of the study.

The DSMB will be asked to evaluate with particular attention the following issues on participant safety concern and study compliance:

1. Adverse events will be recorded by the PI and presented to the DSMB during the regular, scheduled meetings (summarized in a letter).

2. Serious adverse events will be reported to the DSMB within 2-3 calendar days after first becoming aware thereof.

3. Drop-out rates.

Furthermore, the DSMB will be asked to randomly check that the health status of the participating children is being followed every three weeks with the caregivers by the responsible person(s) of the research team.

Data handling and record keeping / archiving

Data will be directly entered in the electronic case report form (eCRF, program Redcap) of each participant. The adverse events (AEs) including severe adverse events (SAEs) will be entered in the eCRFs. All SAEs must be reported immediately and within a maximum of 24 hours to the PI of the study. The investigator will re-evaluate the SAE and return the form to the site. SAEs resulting in death are reported to the local Ethics Committee (via local investigator) within 7 days.

Subjects will be given a unique identity number, the key to which will be kept in a protected data file. During data analysis, data will be connected only to ID numbers without personal information. Data will not be forwarded to third parties and will only remain the property of Stellenbosch University and ETH

Zurich, Switzerland. Data on paper source files will be stored at Stellenbosch University for a minimum of five years and a maximum of ten years after completion of the study. If publications result from the study, all data will be presented anonymously. The PI will notify the accredited Ethical Review Committees of the end of the study within a period of 8 weeks. The end of the study is defined as the last patient's last visit. In case the study is ended prematurely, the PI will notify the accredited Ethical Review Committee, including reasons for the premature termination. The investigator will report to the Ethical Review Committee according to the regulations. After completion of the trial, results will be presented to the caregivers of the participating children and as well to the community in village meetings. Results of the study will be published in a scientific journal and will be presented and discussed at conferences. The trial will be registered on clinicaltrial.gov.

Case Report Forms (CRF)

Data will be recorded using paper and electronic CRFs (Appendix 3). For each enrolled study participant, an eCRF is maintained. eCRFs will be kept current to reflect subjects' status at each phase during the course of the study. There will be no identifier on the eCRF (name or initials), but the study ID will be used.

Trained pediatric study nurses and the local study coordinator will fill in the eCRF. The local study site coordinator will review all data for completeness and accuracy. The co-investigator located both in Zurich and South Africa will regularly review all source data. Source data will be entered into a database using double entry.

Specification of source documents

The eCRF, including informed consent and the AE forms are considered as source data.

Record keeping / archiving

All study data will be archived for a minimum of 10 years after the termination of the study.

Data management system

Coded data will be captured and managed using REDCap (Research Electronic Data Capture), programmed with quality-check parameters to minimize errors during data entry. Data will be backed up on an external hard drive routinely at the end of each day and after each set of data was entered. Copies of this final dataset will be stored at the study site (study site coordinator) and at ETH Zurich, Switzerland (PI).

Data security, access and back-up

The final dataset will be accessible to all investigators named in this application. Back-ups of this final dataset will be stored at the study site (study site coordinator) and at ETH Zurich, Switzerland (PI).

Analysis and archiving

Study data will be archived in our facilities in Cape Town (original study forms and electronic scanned copies). An electronic dataset will also be archived at ETH Zurich, Switzerland.

Electronic and central data validation

The data will be validated using range checks retrieved from previous studies in similar population groups.

Storage of biological material and related health data

Blood and faecal samples will be stored and kept at the HNL (ETH Zurich, Institute of Food, Nutrition and Health, Switzerland) until publication of results. The samples will be labelled with only a code. Access rights to this code are only granted to the PI (Prof. Michael Zimmermann) and co-investigators in Switzerland (Dr. Jeannine Baumgartner) from the HNL, Institute of Food, Nutrition and Health, ETH Zurich, Switzerland. Upon publication, the samples will be destroyed.

Publication and Dissemination Policy

The results of Hb will be shared on the day of blood collection with the health provider and the participating families. The trial results will be summarized in a clear and understandable way to communicate them to the community (community meetings) and participating families.

During the final phase of the project, the ETH Zurich and Stellenbosch University Faculty of Medicine and Health Sciences, Cape Town, South Africa, will work together in the interpretation and dissemination of the findings of the study. Dr. Shaun Barnabas, a pediatric infectious diseases specialist, is the chief co-investigator for the South African team.

After statistical analysis of the study, the PI will make every endeavor to publish the data in an open-access journal or in a pay-walled journal with open-access to ensure global availability.

Confidentiality, Data Protection

The PI and co-investigators are liable to treat the entire information related to the studies and the compiled data strictly confidentially. All assessed personal data will be analyzed in coded form and only used for scientific purpose. Data generation, transmission, archiving and analysis of personal data within these studies, strictly follows good clinical practice (GCP) regulation as well as the regulation on professional secrecy in clinical research. Prerequisite is the voluntary approval of the participant given by signing the informed consent prior start of participation of the study. Individual participant medical information obtained as a result of these studies is considered confidential and disclosure to third parties is prohibited. Participant confidentiality will be further ensured by utilizing participant identification code numbers to correspond to treatment data in the computer files. Such medical information may be given to the participant's personal physician or to other appropriate medical personnel responsible for the participant's welfare, if the patient has given his/her written consent to do so.

3.3 Risk-benefit analysis

This study will form part of our current research efforts aimed at providing optimized recommendations on the treatment of ID and IDA in HIV-infected children in South Africa in order to improve their health, well-being, cognitive development and school performance, and therefore their long-term prognosis and quality of life. All children participating in this study will receive daily oral iron supplements, providing 50

mg elemental iron in the form of FeFum, for an intervention period of 12 weeks, which is aligned with the South African Standard Treatment Guidelines and Essential Medicines List for the treatment of iron deficiency and anaemia in children, including children living with HIV (47). Thus, all children may benefit from improving their iron status by the iron intervention. The addition of GOS, which is a prebiotic fiber, does not pose any known risk to the children. In contrary, we hypothesize that the addition of GOS will mitigate some of the potentially adverse effects of iron on the gut microbiome and gut inflammation, and will enhance iron absorption. GOS is considered a novel food supplement with a consumption up to 12 g GOS/day regarded as safe by EFSA (59), and is already routinely added to infant formula and cereals around the world.

4. Bibliography

1. UNAIDS. Global report: report on the global AIDS epidemic Geneva: WHO Library; 2010 [Available from: http://files.unaids.org/en/media/unaids/contentassets/documents/unaidspublication/2010/20101123_globalreport_en[1].pdf.
2. WHO. The global burden of disease: 2004 update. Geneva: World Health Organization2008.
3. Kassebaum NJ, Jasrasaria R, Naghavi M, Wulf SK, Johns N, Lozano R, et al. A systematic analysis of global anemia burden from 1990 to 2010. Blood. 2014;123(5):615-24.
4. Jonker FAM, te Poel E, Bates I, Boele van Hensbroek M. Anaemia, iron deficiency and susceptibility to infection in children in sub-Saharan Africa, guideline dilemmas. Br J Haematol. 2017;177(6):878-83.
5. Ellaurie M, Burns ER, Rubinstein A. Hematologic manifestations in pediatric HIV infection: severe anemia as a prognostic factor. Journal of Pediatric Hematology/Oncology. 1990;12(4):449-53.
6. Clark T, Mmiro F, Ndugwa C, Perry R, Jackson J, Melikian G, et al. Risk factors and cumulative incidence of anaemia among human immunodeficiency virus-infected children in Uganda. Annals of tropical paediatrics. 2002;22(1):11-7.
7. Belperio PS, Rhew DC. Prevalence and outcomes of anemia in individuals with human immunodeficiency virus: a systematic review of the literature. The American journal of medicine. 2004;116(7):27-43.
8. Abioye AI, Andersen CT, Sudfeld CR, Fawzi WW. Anemia, Iron Status, and HIV: A Systematic Review of the Evidence. Adv Nutr. 2020;11(5):1334-63.
9. Horton S, Ross J. The economics of iron deficiency. Food Policy. 2003;28(1):51-75.
10. Rawat R, Humphrey JH, Ntozini R, Mutasa K, Iliff P, Stoltzfus R. Elevated iron stores are associated with HIV disease severity and mortality among postpartum women in Zimbabwe. Public health nutrition. 2009;12(9):1321-9.
11. Weiss G, Goodnough LT. Anemia of chronic disease. New England Journal of Medicine. 2005;352(10):1011-23.
12. Drakesmith H, Prentice AM. Hepcidin and the iron-infection axis. Science. 2012;338(6108):768-72.
13. Nasi M, Pinti M, Mussini C, Cossarizza A. Persistent inflammation in HIV infection: established concepts, new perspectives. Immunology letters. 2014;161(2):184-8.
14. Armitage AE, Stacey AR, Giannoulatou E, Marshall E, Sturges P, Chatha K, et al. Distinct patterns of hepcidin and iron regulation during HIV-1, HBV, and HCV infections. Proceedings of the National Academy of Sciences. 2014;111(33):12187-92.
15. O'brien ME, Kupka R, Msamanga GI, Saathoff E, Hunter DJ, Fawzi WW. Anemia is an independent predictor of mortality and immunologic progression of disease among women with HIV in Tanzania. Journal of Acquired Immune Deficiency Syndromes. 2005;40(2):219-25.
16. Savarino A, Pescarmona GP, Boelaert JR. Iron metabolism and HIV infection: reciprocal interactions with potentially harmful consequences? Cell biochemistry and function. 1999;17(4):279-87.
17. Wisaksana R, de Mast Q, Alisjahbana B, Jusuf H, Sudjana P, Indrati AR, et al. Inverse relationship of serum hepcidin levels with CD4 cell counts in HIV-infected patients selected from an Indonesian prospective cohort study. PLoS One. 2013;8(11):e79904.

18. Al-Khafaji B, Kralovic S, Smith RD. Increased hepatic iron in the acquired immunodeficiency syndrome: an autopsy study. Modern pathology: an official journal of the United States and Canadian Academy of Pathology, Inc. 1997;10(5):474-80.
19. De Monyé C, Karcher DS, Boelaert JR, Gordeuk VR. Bone marrow macrophage iron grade and survival of HIV-seropositive patients. Aids. 1999;13(3):375-80.
20. Minchella P, Armitage A, Darboe B, Jallow M, Drakesmith H, Jaye A, et al. Elevated hepcidin at HIV diagnosis is associated with incident tuberculosis in a retrospective cohort study. The international journal of tuberculosis and lung disease. 2014;18(11):1337-9.
21. Cercamondi CI, Egli IM, Ahouandjinou E, Dossa R, Zeder C, Salami L, et al. Afebrile Plasmodium falciparum parasitemia decreases absorption of fortification iron but does not affect systemic iron utilization: a double stable-isotope study in young Beninese women. The American journal of clinical nutrition. 2010;92(6):1385-92.
22. Esan MO, van Hensbroek MB, Nkhoma E, Musicha C, White SA, Ter Kuile FO, et al. Iron supplementation in HIV-infected Malawian children with anemia: a double-blind, randomized, controlled trial. Clinical infectious diseases. 2013;57(11):1626-34.
23. Olsen A, Mwaniki D, Krarup H, Friis H. Low-dose iron supplementation does not increase HIV-1 load. Journal of Acquired Immune Deficiency Syndromes. 2004;36(1):637-8.
24. Semba RD, Ricketts EP, Mehta S, Netski D, Thomas D, Kirk G, et al. Effect of micronutrients and iron supplementation on hemoglobin, iron status, and plasma hepatitis C and HIV RNA levels in female injection drug users: a controlled clinical trial. Journal of Acquired Immune Deficiency Syndromes. 2007;45(3):298-303.
25. Zimmermann MB, Chassard C, Rohner F, N'Goran EK, Nindjin C, Dostal A, et al. The effects of iron fortification on the gut microbiota in African children: a randomized controlled trial in Cote d'Ivoire. The American journal of clinical nutrition. 2010;92(6):1406-15.
26. Jaeggi T, Kortman GA, Moretti D, Chassard C, Holding P, Dostal A, et al. Iron fortification adversely affects the gut microbiome, increases pathogen abundance and induces intestinal inflammation in Kenyan infants. Gut. 2014:gutjnl-2014-307720.
27. Klatt NR, Funderburg NT, Brenchley JM. Microbial translocation, immune activation, and HIV disease. Trends in microbiology. 2013;21(1):6-13.
28. Hunt PW, Sinclair E, Rodriguez B, Shive C, Clagett B, Funderburg N, et al. Gut epithelial barrier dysfunction and innate immune activation predict mortality in treated HIV infection. The Journal of infectious diseases. 2014;210(8):1228-38.
29. Africa. RoS. Essential Drugs Programme. Primary Healthcare Standard Treatment Guidelines and Essential Medicine List. In: Health NDo, editor. 6 ed2018.
30. Brentlinger PE, Silva WP, Vermund SH, Valverde E, Buene M, Moon TD. Practical Management of HIV-Associated Anemia in Resource-Limited Settings: Prospective Observational Evaluation of a New Mozambican Guideline. AIDS research and human retroviruses. 2016;32(1):12-25.
31. Adetifa I, Okomo U. Iron supplementation for reducing morbidity and mortality in children with HIV. The Cochrane Library. 2009.
32. Jeroense FMD, Michel L, Zeder C, Herter-Aeberli I, Zimmermann MB. Consumption of Galacto-Oligosaccharides Increases Iron Absorption from Ferrous Fumarate: A Stable Iron Isotope Study in Iron-Depleted Young Women. J Nutr. 2019;149(5):738-46.
33. Paganini D, Uyoga MA, Cercamondi CI, Moretti D, Mwasi E, Schwab C, et al. Consumption of galacto-oligosaccharides increases iron absorption from a micronutrient powder containing ferrous fumarate and sodium iron EDTA: a stable-isotope study in Kenyan infants. Am J Clin Nutr. 2017;106(4):1020-31.
34. Paganini D, Uyoga MA, Kortman GAM, Cercamondi CI, Moretti D, Barth-Jaeggi T, et al. Prebiotic galacto-oligosaccharides mitigate the adverse effects of iron fortification on the gut microbiome: a randomised controlled study in Kenyan infants. Gut. 2017;66(11):1956-67.
35. Carlson JL, Erickson JM, Lloyd BB, Slavin JL. Health Effects and Sources of Prebiotic Dietary Fiber. Curr Dev Nutr. 2018;2(3):nzy005-nzy.
36. Davani-Davari D, Negahdaripour M, Karimzadeh I, Seifan M, Mohkam M, Masoumi SJ, et al. Prebiotics: Definition, Types, Sources, Mechanisms, and Clinical Applications. Foods. 2019;8(3):92.
37. Jeroense FMD, Zeder C, Zimmermann MB, Herter-Aeberli I. Acute Consumption of Prebiotic Galacto-Oligosaccharides Increases Iron Absorption from Ferrous Fumarate, but not from Ferrous Sulfate and Ferric Pyrophosphate: Stable Iron Isotope Studies in Iron-Depleted Young Women. The Journal of Nutrition. 2020;150(9):2391-7.
38. Vujkovic-Cvijin I, Sortino O, Verheij E, Sklar J, Wit FW, Kootstra NA, et al. HIV-associated gut dysbiosis is independent of sexual practice and correlates with noncommunicable diseases. Nature Communications. 2020;11(1):2448.
39. Zimmermann MB, Hurrell RF. Nutritional iron deficiency. The Lancet. 2007;370(9586):511-20.

40. Righetti AA, Koua A-YG, Adiossan LG, Glinz D, Hurrell RF, N'Goran EK, et al. Etiology of anemia among infants, school-aged children, and young non-pregnant women in different settings of south-central Côte d'Ivoire. The American journal of tropical medicine and hygiene. 2012;87(3):425-34.
41. Zimmermann MB, Molinari L, Staubli-Asobayire F, Hess SY, Chaouki N, Adou P, et al. Serum transferrin receptor and zinc protoporphyrin as indicators of iron status in African children. Am J Clin Nutr. 2005;81(3):615-23.
42. WHO. Assessing the iron status of populations: report of a Joint World Health Organization/Centers for Disease Control and Prevention Technical Consultation on the Assessment of Iron Status at the Population Level, Geneva, Switzerland, 6-8 April 2004. Assessing the iron status of populations: report of a Joint World Health Organization/Centers for Disease Control and Prevention Technical Consultation on the Assessment of Iron Status at the Population Level, Geneva, Switzerland, 6-8 April 20042005.
43. Speich C, Wegmüller R, Brittenham GM, Zeder C, Cercamondi CI, Buhl D, et al. Measurement of long-term iron absorption and loss during iron supplementation using a stable isotope of iron (57Fe). Br J Haematol.n/a(n/a).
44. Fomon SJ, Nelson SE, Serfass RE, Ziegler EE. Absorption and loss of iron in toddlers are highly correlated. The Journal of nutrition. 2005;135(4):771-7.
45. Green R, Charlton R, Seftel H, Bothwell T, Mayet F, Adams B, et al. Body iron excretion in man: a collaborative study. The American journal of medicine. 1968;45(3):336-53.
46. Hunt JR, Zito CA, Johnson LK. Body iron excretion by healthy men and women. The American journal of clinical nutrition. 2009;89(6):1792-8.
47. NDOH. Essential Drugs Programme. Primary Healthcare Standard Treatment Guideline and Essential Medicine List. Pretoria, Republic of South Africa2018.
48. Bertelsen RJ, Jensen ET, Ringel-Kulka T. Use of probiotics and prebiotics in infant feeding. Best Pract Res Clin Gastroenterol. 2016;30(1):39-48.
49. Amador-Licona N, Díaz-Murillo TA, Gabriel-Ortiz G, Pacheco-Moises FP, Pereyra-Nobara TA, Guízar-Mendoza JM, et al. Omega 3 fatty acids supplementation and oxidative stress in HIV-seropositive patients. A clinical trial. PloS one. 2016;11(3):e0151637.
50. WHO. Iron deficiency anaemia: assessment, prevention and control: a guide for programme managers. 2001.
51. Pfeiffer CM, Cook JD, Mei Z, Cogswell ME, Looker AC, Lacher DA. Evaluation of an automated soluble transferrin receptor (sTfR) assay on the Roche Hitachi analyzer and its comparison to two ELISA assays. Clinica chimica acta; international journal of clinical chemistry. 2007;382(1-2):112-6.
52. Erhardt JG, Estes JE, Pfeiffer CM, Biesalski HK, Craft NE. Combined measurement of ferritin, soluble transferrin receptor, retinol binding protein, and C-reactive protein by an inexpensive, sensitive, and simple sandwich enzyme-linked immunosorbent assay technique. Journal of nutrition. 2004;134(11):3127-32.
53. Walmsley T, George P, Fowler R. Colorimetric measurement of iron in plasma samples anticoagulated with EDTA. Journal of clinical pathology. 1992;45(2):151-4.
54. Jaeggi T, Kortman GA, Moretti D, Chassard C, Holding P, Dostal A, et al. Iron fortification adversely affects the gut microbiome, increases pathogen abundance and induces intestinal inflammation in Kenyan infants. Gut. 2015;64(5):731-42.
55. Paganini D, Uyoga MA, Kortman GA, Cercamondi CI, Moretti D, Barth-Jaeggi T, et al. Prebiotic galacto-oligosaccharides mitigate the adverse effects of iron fortification on the gut microbiome: a randomised controlled study in Kenyan infants. Gut. 2017;66(11):1956-67.
56. Markowiak P, Śliżewska K. Effects of Probiotics, Prebiotics, and Synbiotics on Human Health. Nutrients. 2017;9(9):1021.
57. Zimmermann MB, Fucharoen S, Winichagoon P, Sirankapracha P, Zeder C, Gowachirapant S, et al. Iron metabolism in heterozygotes for hemoglobin E (HbE), α-thalassemia 1, or β-thalassemia and in compound heterozygotes for HbE/β-thalassemia. The American journal of clinical nutrition. 2008;88(4):1026-31.
58. Hotz K, Krayenbuehl P-A, Walczyk T. Mobilization of storage iron is reflected in the iron isotopic composition of blood in humans. Journal of Biological Inorganic Chemistry. 2012;17(2):301-9.
59. EFSA Panel on Nutrition NF, Allergens F, Turck D, Castenmiller J, De Henauw S, Hirsch-Ernst KI, et al. Safety of a change in the conditions of use of galacto-oligosaccharides as a novel food ingredient in food supplements pursuant to Regulation (EU) 2015/2283. EFSA Journal. 2021;19(1):e06384.

C. Attachments

Attachment 1

PARTICIPANT INFORMATION LEAFLET AND CONSENT FORM

Please see Section 8 of our Health Research Ethics Committee (HREC) Standard Operating Procedures (SOPs) for more detailed information about requirements for Informed Consent (IC). You will find the SOPs here: http://www.sun.ac.za/english/faculty/healthsciences/rdsd/Pages/Ethics/SOP.aspx.
(Please delete this paragraph before submitting your Informed Consent Form (ICF) to the HREC)

**TITLE OF RESEARCH PROJECT:**	
Improving efficacy and safety of oral iron supplementation in HIV-infected children by providing prebiotic galacto-oligosaccharides as adjunct treatment: A randomized controlled trial	
**DETAILS OF PRINCIPAL INVESTIGATOR (PI):**	
**Title, first name, surname:** Dr Shaun Barnabas	**Ethics reference number:**
**Full postal address:** FAM-CRU, Ward J8, Tygerberg Hospital	**PI Contact number:** 021 938 4295

We would like to invite your child to take part in a research project. Please take some time to read the information presented here, which will explain the details of this project. Please ask the study staff or doctor any questions about any part of this project that you do not fully understand. It is very important that you are completely satisfied that you clearly understand what this research entails and how you could be involved. Also, your participation is **entirely voluntary** and you are free to decline to participate. In other words, you may choose to take part, or you may choose not to take part. Nothing bad will come of it if you say no: it will not affect you negatively in any way whatsoever. Refusal to participate will involve no penalty or loss of benefits or reduction in the level of care to which you are otherwise entitled. You are also free to withdraw from the study at any point, even if you do agree to take part initially.

The Health Research Ethics Committee at Stellenbosch University has approved this study. The study will be conducted according to the ethical guidelines and principles of the international Declaration of Helsinki, the South African Guidelines for Good Clinical Practice (2006), the Medical Research Council (MRC) Ethical Guidelines for Research (2002), and the Department of Health Ethics in Health Research: Principles, Processes and Studies (2015).

What is this research study all about?

- This study will involve 100 children aged 10 to 15 years and will be done at the FAM-CRU unit.
- In this study, we want to learn how to improve iron absorption in HIV-positive children to prevent low iron levels and thereby improving development and health. All children with low iron stores will receive iron supplements for a 3-month period. Half of the children will also receive a natural fibre product (galacto-oligosaccharides) to consume together with the iron every day. This soluble fibre product acts as a prebiotic. Prebiotics are non-digestible food ingredients that cannot be digested by our body, but serve as food for bacteria in the gut, especially healthy bacteria. We have previously

shown in healthy Kenyan infants that taking this natural fibre product can improve the absorption of supplemental iron and reduce some of the side-effects of the iron on the gut.

In this study, we want to find out, whether the soluble fibre product can help improving iron absorption and make iron supplementation safer in HIV-positive children with low iron levels.

- If you are willing to participate and your child fulfils all the inclusion criteria, you have to sign the informed consent form. As a participant, you will be asked to bring your child to the FAM-CRU unit at Tygerberg Hospital for 4 study visits, over a period of 4 months.

The following will happen at every visit:

- **Study visit 1:** We will take a blood sample (10 mL = 2 teaspoons) from your child to see how much iron and signs of infection your child has in his or her blood. During this visit, we will also look at your child's health, and measure your child's height and weight. We will also provide you with a kit to collect a stool sample at home and bring with on your next visit. Stool sample collection will be explained to you and your child in detail by the study staff. This sample and all following ones will be used to look at signs of infection and the balance of healthy and unhealthy bacteria in the gut of your child.

- **Study visit 2, 3 and 4:** If you child has ***low iron levels*** based on the blood sample taken on visit 1, we will ask you and your child to participate in the study and you will be asked to come for study visits 2, 3 and 4. On visits 2, 3 and 4, we will do a short interview with you and your child about the foods typically eaten, we will look at your child's health and measure weight and height. We will take another blood sample (10 mL = 2 teaspoons) from your child to look for any signs of infection and to measure the amount of iron in your child's blood. On visit 3 we will again provide you with a kit to collect a stool sample at home and bring with on study visit 4.

- All children will be provided with iron tables to take daily for 3 months to treat the low iron levels in the blood. All children will also receive a powder product to take in daily together with the iron tablets. In half of the cases this powder will consist of fibre and in the other half of cases this will be a natural sugar. You will receive clear instructions on how to take all supplements.

- We will monitor the health of your child every month by phone calls and will ask if your child had any signs or symptoms of illness.

Why have you been invited to participate?

- In order to study ways to improve iron levels in children, we need to study children with HIV and low iron levels. We think that your child might fit the inclusion criteria and that is why we invite you and your child to consider taking part in this study.

What will your responsibilities be?

If you are participating in the study, you will have to respect certain rules. They are necessary to make the study successful.

- Be at the hospital in time for the scheduled appointments.
- Comply with the study plan.
- Inform the person in charge of the study of any disease, infection, symptoms, pain or change of condition in your child.
- Inform the person in charge of the study about other treatments and medicines.

Will you benefit from taking part in this research?

- Benefits of the study include the iron tablets which is likely to have a positive effect on your child's iron levels. Your child's health will be closely watched throughout the study and sick children will be treated free of charge. This study will also benefit other children in future since we hope to find better ways in preventing and treating low iron levels.

Are there any risks involved in your taking part in this research?

- Participation includes the small risks of bruising, infection, and discomfort during blood drawn from the child. To minimize these risks, trained and experienced nurses will draw blood using sterile equipment and techniques. A cream, Emla, will be used to numb the skin before each blood draw.
- Taking iron supplements can have some side effects, such as stomach upset and pain, constipation, diarrhoea, nausea or vomiting. Especially when starting to take the supplements. If your child is experiencing any of these side effects or any other health problem, please contact the study team.
- There is no known risk associated with the fibre supplement.

If you do not agree to take part, what alternatives do you have?

- You and your child will only participate in this study if you are both willing to participate. No one can force you or your child in any other way to participate. You will not have to comment on why you or your child do not want to participate. If you or your child have made your decision, your child can withdraw from the study at any time, without giving reasons and without consequences. If your child has low iron levels and you or your child decide to withdraw at any time, iron tablets can be accessed at any primary health care clinic.

Who will have access to your medical records?

- The researchers will treat all information collected as private and confidential. This means that your name or your child's names will not appear on the research forms or any of the reports. A separate list which connects your name to the study number will be kept locked in a separate file from the data and will not be seen by anyone except the study team. Your and your child's identity will not be given when our findings are communicated, including publication of the data in science journals. Only information about the entire group that participated will be reported. No information about you or your child, or information that connects you to the study, will be given to anyone without your wish and your written permission. At any time during the study, you can ask to see your child's data.

What will happen in the unlikely event of some form of injury occurring as a direct result of your taking part in this research study?

- In the unlikely case of harm associated with the study, you child will receive immediate medical treatment. All cost for medical treatment caused by harmful consequences of the study will be covered by insurance from Stellenbosch University. Accidents on the way to and from Tygerberg Hospital are not included in this insurance.

Will you be paid to take part in this study and are there any costs involved?

- You as caregiver will receive an incentive for your time, inconvenience and expenses for each scheduled study visit. There will be no costs involved for you, if you do take part.

Is there anything else that you should know or do?

- This study will be conducted in collaboration with researchers at the Laboratory of Human Nutrition of ETH Zürich in Switzerland. The study has also been approved by the Ethics Committee of ETH Zürich.
- You should inform your family practitioner or usual doctor that you are taking part in a research study.
- You can contact Dr S Barnabas on 021 938 5295 or after hours on 084 641 0735 if you have any further queries or problems.
- You can contact the Health Research Ethics Committee at 021 938 9207 if you have any concerns or complaints that have not been adequately addressed by your study doctor/staff.
- You will receive a copy of this information and consent form for your own records.

Declaration by participant

By signing below, I ……………………………….…………. agree to take part in a research study entitled *Improving efficacy and safety of oral iron supplementation in HIV-infected children by providing prebiotic galacto-oligosaccharides as adjunct treatment: A randomized controlled trial*

I declare that:

- I have read this information and consent form, or it was read to me, and it is written in a language in which I am fluent and with which I am comfortable.
- I have had a chance to ask questions and I am satisfied that all my questions have been answered.
- I understand that taking part in this study is **voluntary,** and I have not been pressurised to take part.
- I may choose to leave the study at any time and nothing bad will come of it – I will not be penalised or prejudiced in any way.
- I may be asked to leave the study before it has finished, if the study doctor or researcher feels it is in my best interests, or if I do not follow the study plan that we have agreed on.

Signed at (*place*) ....…........…………….. on (*date*) …………....……….. 2021.

....	....
**Signature of participant**	**Signature of witness**

Declaration by investigator

I *(name)* ……………………………………………..……… declare that:

- I explained the information in this document in a simple and clear manner to …………………………………..
- I encouraged him/her to ask questions and took enough time to answer them.
- I am satisfied that he/she completely understands all aspects of the research, as discussed above.
- I did/did not use an interpreter. (*If an interpreter is used then the interpreter must sign the declaration below.)*

Signed at (*place*) ....….......……………. on (*date*) …………....……….. 2021.

....

Signature of investigator **Signature of witness**

Permission to have all anonymous data shared with journals:

Please carefully read the statements below (or have them read to you) and think about your choice. No matter what you decide, it will not affect whether you can be in the research study, or your routine health care

When this study is finished, we would like to publish results of the study in journals. Most journals require us to share your anonymous data with them before they publish the results. Therefore, we would like to obtain your permission to have your anonymous data shared with journals.

Permission for sharing samples and/or information with other investigators:

Please carefully read the statements below (or have them read to you) and think about your choice. No matter what you decide, it will not affect whether you can be in the research study, or your routine health care.

In order to do the research we have discussed, we must collect and store blood and stool samples and health information from people like you that are HIV positive and have low iron levels]. We will do some of the tests right away. Other tests may be done in the future. Once we have done the research that we are planning for this research project, we would like to store your sample and/or information. The researchers that are working with us on this study are based in Switzerland. Some of your blood and stool samples will be sent to them for analysis in the current and future research. To protect your privacy, we will replace your name with a unique study number. We will only use this code for your sample and information about you. We will do our best to keep the code private. It is however always possible that someone could find out about your name but this is very unlikely to happen. Therefore, we would like to ask for your permission to share your samples and information with other investigators.

Tick the Option you choose for anonymous data sharing with journals:

I agree to have my anonymous data shared with journals during publication of results of this study

☐ Signature____________

OR

I do not agree to have my anonymous data shared with journals during publication of results of this study

☐ Signature____________

Tick the Option you choose for sharing samples and/or information with other investigators:

I do not want my sample and/or information to be shared with other investigators

☐ Signature______________

OR

My sample and/or information may be shared with other investigators for further analysis and future research in a field related to iron deficiency.

☐ Signature______________

Attachment 2

PARTICIPANT INFORMATION LEAFLET AND ASSENT FORM

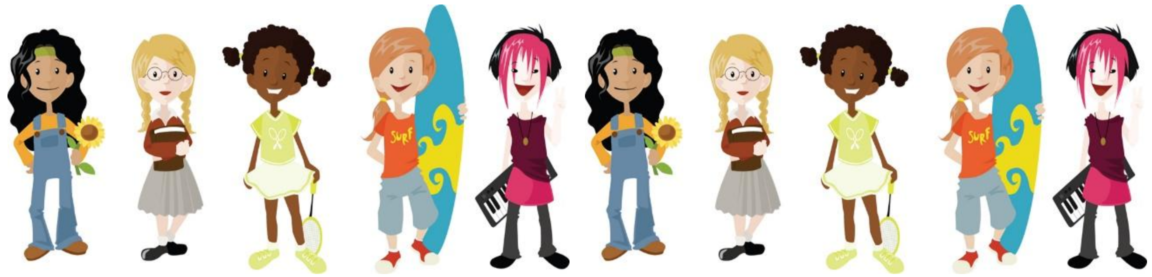

TITLE OF THE RESEARCH PROJECT: Testing new ways to improve the effect and safety of oral iron tables in HIV-infected children

RESEARCHERS NAME(S): Dr S Barnabas

ADDRESS: FAM-CRU, Ward J8, Tygerberg Hospital

CONTACT NUMBER: 021 938 4295

Hello, my name is *(name, surname)* and I would like to ask if you would like to take part in a research project.

What is research?

- Research is something we do to find new answers about the way things (and people) work. We use research projects or studies to help us find out more about disease or illness. Research also helps us to find better ways of helping or treating children who are sick.

What is this research project all about?

- Food has building blocks to keep us healthy and to help us grow. We want to find out more about iron, one of the building blocks in food, and find out how a drink that contains fibres from foods can help your body taking up and tolerate the iron from a supplement.

Why have I been invited to take part in this research project?

- To find the answers we are looking for, we need to do a few tests in children with low iron levels, and with HIV. We think that you might fall into any of these groups and that is why we ask you to think about taking part in this study.

Who is doing the research?

- The research is done by researchers from Stellenbosch University (Tygerberg) and a university in Switzerland in Europe, ETH Zurich.

What will happen to me in this study?

- There will be 100 children from 10 to 15 years in this study.
- If you say yes to take part, then you have to sign a special form. As a participant, you will visit the FAM-CRU unit at Tygerberg Hospital 4 times over a period of 3 months.
- The visits will not be the same each time and the following will happen at different visits:
  - We will look at your health and ask some questions.
  - We will measure your weight and height.
  - We will draw blood.
  - We will ask you to bring a stool (poo) sample (this you will collect at home and bring with in a closed plastic bottle).
  - We will talk about foods that you eat on most days.
  - You will drink iron tablets for 3 months.
  - You will drink a sachet of powder daily for 3 months.

The blood and stool samples collected will be sent to a laboratory in Switzerland for some of the tests.

Can anything bad happen to me?

- There is a small chance of bruising, infection, and soreness during blood draw. Only a trained nurse will draw blood. A cream will be used to numb the skin before each blood draw.

Can anything good happen to me?

- Yes, if the iron level in your blood is low, we will give you iron tablets that will possibly make the iron level in your blood higher. We will also keep a close eye on your health and treat any illness. Other children can also be helped in future if we find out how to prevent low iron levels.

Will anyone know I am in the study?

- No, the researchers will not tell anyone and your information will only be seen by the research team. Your name will not be on any forms or reports. When we share the information of the group with others, no names will be mentioned.

Who can I talk to about the study?

- You can talk to Dr S Barnabas at FAM-CRU if you have any questions or problems. His number is 021 938 5295 and after hours you can phone 084 641 0735.

What if I do not want to do this?

- It is your choice to take part or not. It is okay if you do not want to take part in this study. No one can force you and you do not have to tell anyone why you do not want to take part. You can decide not to take part even if your parent said yes to

take part in the study. If you decide to take part, you can stop being in the study at any time without getting in trouble.

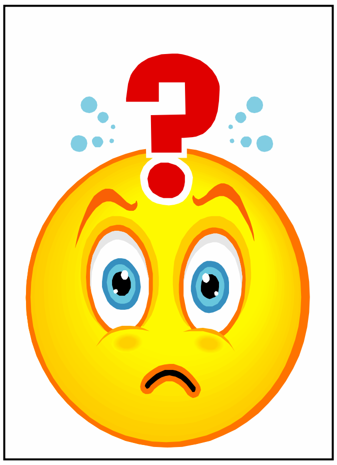

Do you understand this research study and are you willing to take part in it?

YES NO

Has the researcher answered all your questions?

YES NO

Do you understand that you can pull out of the study at any time?

YES NO

______________________________ ______________________________

Name and Surname of child **Signature of child**

__________________ __________________ __________________

Place Date Time

Person obtaining informed consent and assent process:

I am satisfied that the child understands what the consent form is about and that his/her questions have been answered.

Name and Surname **Signature**

Place Date Time

Eidgenössische Technische Hochschule Zürich
Swiss Federal Institute of Technology Zurich

Attachment 3

CRF - Case Report Form

Improving efficacy and safety of oral iron supplementation in HIV-infected children by providing prebiotic galacto-oligosaccharides as adjunct treatment: A randomized controlled trial

Version 1.0 / 08.10.2020

Dr. MD Shaun Barnabas

Family Centre for Research with Ubuntu (FAM-CRU)

Department of Pediatrics and Child Health

Faculty of Medicine and Health Sciences

Stellenbosch University

[redacted]

OVERVIEW OF PROCEDURES AND ASSESSMENTS

	Screening	Baseline	Midpoint	Endpoint
			12-week intervention period	
**Visit**	**0**	**1**	**2**	**3**
**Day**	**-45**	**0**	**42 (±3)**	**84 (±3)**
Informed consent	X			
Assignment to subject ID	X			
Checking in/exclusion criteria	X			
Socio-demographic information	X			
Health check/medical history	X	X	X	X
Randomisation to intervention	X			
Anthropometry (height/weight)	X	X	X	X
Venous blood sampling	X[a]	X[b]	X[c]	X[b]
Faecal sample collection		X		X
Distribution of supplements		X	X	X
Compliance monitoring			X	X
Adverse event / side effect monitoring		X	X	X

[a]Iron status: Full blood count, plasma ferritin.

[b]Iron status: Full blood count, plasma ferritin, soluble transferring receptor, transferrin saturation; ^{57}Fe isotopic dilution; hepcidin; systemic inflammation: C-reactive protein, alpha-1 glycolic acid; gut mucosal integrity.

[c]Iron status: Full blood count, plasma ferritin, soluble transferring receptor, transferrin saturation; ^{57}Fe isotopic dilution; systemic inflammation: C-reactive protein, alpha-1 glycolic acid.

VISIT 0 (DAY 0 - 21): SCREENING – 1/4

Date of visit 0 (Day -45) (dd/mm/yy) ☐☐/☐☐/☐☐

INFORMED CONSENT

Date Informed Consent signed (dd/mm/yy) ☐☐/☐☐/☐☐

CHILD DATA

Date of birth of child	(dd/mm/yy) ☐☐/☐☐/☐☐
Child's age at screening	(in years) ☐☐
Source	☐ Birth certificate ☐ Mother ☐ Child health booklet<br>Other:_______________
Child sex	☐ Male ☐ Female

Anthropometrics:

Length	1st measurement: ☐☐☐.☐ cm
	2nd measurement: ☐☐☐.☐ cm
	(3rd measurement: ☐☐☐.☐ cm)
	Mean = ☐☐☐.☐ cm
Weight	1st measurement: ☐☐☐.☐ kg
	2nd measurement: ☐☐☐.☐ kg
	(3rd measurement: ☐☐☐.☐ kg)
	Mean = ☐☐☐.☐ kg
Z-score weight-for-age	= ____________________
Z-score weight-for-height	= ____________________
Z-score height-for-age	= ____________________

CHILD HEALTH STATUS AND DRUG INTAKE PAST 4 WEEKS AND CURRENTLY

Chronic disease	☐ yes ☐ no If yes, treatment…………………
Fever	☐ yes ☐ no If yes, treatment…………………
Hospitalization	☐ yes ☐ no If yes, treatment…………………
Vomiting for long time	☐ yes ☐ no If yes, treatment…………………
Cough or difficult breathing	☐ yes ☐ no If yes, treatment…………………
Diagnosis of pneumonia	☐ yes ☐ no If yes, treatment…………………
Diagnosis of tuberculosis	☐ yes ☐ no If yes, treatment…………………
Gastrointestinal disorders	☐ yes ☐ no If yes, treatment…………………
Diarrhea	☐ yes ☐ no If yes, treatment…………………
Blood or mucus in the stool	☐ yes ☐ no If yes, treatment…………………
Malaria	☐ yes ☐ no If yes, treatment…………………
Ear infection	☐ yes ☐ no If yes, treatment…………………
Sores in mouth	☐ yes ☐ no If yes, treatment…………………
Signs of lethargy / unconsciousness	☐ yes ☐ no If yes, treatment…………………
Signs of severe dehydration	☐ yes ☐ no If yes, treatment…………………
Palmar pallor	☐ yes ☐ no If yes, treatment…………………
Jaundice	☐ yes ☐ no If yes, treatment…………………
Any other illness	☐ yes ☐ no If yes, treatment…………………
Specify and list other current treatments/medication	____
Vitamin-mineral supplements in the past 4 weeks and currently	☐ yes ☐ no<br>if yes, specify type and duration:<br>________________________________
**Antibiotics** in the past 4 weeks	☐ yes ☐ no<br>If yes, specify:<br>Diagnosis:________________<br>Type of antibiotics:________________

	Date (dd/mm/yy) of first dose administration:
	☐☐/☐☐/☐☐
	Date (dd/mm/yy) of last dose administration:
	☐☐/☐☐/☐☐
	Duration of treatment: ____days
**Tuberculosis** treatment in the past 4 weeks	☐ yes ☐ no
	If yes, specify:
	Diagnosis:_______________
	Type of treatment:_______________
	Date (dd/mm/yy) of first dose administration:
	☐☐/☐☐/☐☐
	Date (dd/mm/yy) of last dose administration:
	☐☐/☐☐/☐☐
	Duration of treatment: ____days
**Antiretroviral therapy** in the past 4 weeks	☐ yes ☐ no

HIV CRITERIA

Is the child HIV-infected?	☐ yes ☐ no
ART start date	Year: ☐☐☐☐
CD4+ Cells	
	Cells/mm____________________
HIV RNA viral load	
	copies/mL____________________

BLOOD SAMPLING & ANALYSIS

Time blood sampling	☐☐:☐☐
Sample labeling	Subject ID___V0:______
Comments about specimen collection/absence/rescheduling	____

Date full blood count	☐☐/☐☐/☐☐☐☐
**Hemoglobin** (from full blood count)	g/dL ☐☐.☐
MCV (from full blood count)	fL ☐☐.☐
Ferritin	µg/L ☐☐☐.☐
sTfR	mg/L ☐☐.☐

INCLUSION CRITERIA

Subjects must meet all of the following criteria to be eligible to participate in this study

1	Child aged 10-15 years	☐ yes ☐ no
2	Mild to moderate micro- and normocytic anaemia defined as Hb ≥8.0 and <11.5 / 12 g/dL (children 8–11 / 12–14 years) plus MCV ≤91.5 fL and/or ID defined as ferritin <30 µg/L or sTfR >8.3 mg/L	☐ yes ☐ no
3	Body-Mass-Index-for-age Z-scores (BAZ) −3 to 2 SD of reference population	☐ yes ☐ no
4	HIV RNA viral load <50 copies/mL (measured as part of routine care)	☐ yes ☐ no
5	CD4+ T cell counts ≥500 cells/mm^3	☐ yes ☐ no
6	Willingness of caregiver to participate in the study	☐ yes ☐ no
7	Caregiver speaks English, Afrikaans or isiXhosa	☐ yes ☐ no
8	The informed consent form has been read and signed by the caregiver (or has been read out to the caregiver in case of illiteracy) plus assent needs to be obtained from the child	☐ yes ☐ no
9	Residence in the study area for the period of the study.	☐ yes ☐ no

If one of the answers is NO, the subject cannot participate in the study without prior permission from the principal investigators

Were any inclusion criteria DEVIATIONS approved by the principal investigator?	☐ yes, please specify inclusion criteria number ☐<br>Specify the reason<br>…………………………….<br>☐ no<br>☐ N/A

EXCLUSION CRITERIA

Subjects with any of the following characteristics will not be eligible to participate in the study

1	Child received iron supplements or antibiotic treatment 4 weeks prior to study start	☐ yes ☐ no
2	Acute illness or other conditions that in the opinion of the PI or co-researchers would jeopardize the safety or rights of a participant in the trial or would render the participant unable to comply with the protocol	☐ yes ☐ no
3	Participant is taking part in other studies involving medical or physical interventions	☐ yes ☐ no

If one of the answers is YES, the subject cannot participate in the study without prior permission from the principal investigators

Were any exclusion criteria DEVIATIONS approved by the principal investigator?	☐ yes, please specify inclusion criteria number ☐<br>Specify the reason<br>…………………………….<br>☐ no<br>☐ N/A

ELIGIBILITY

The subject meets all inclusion criteria	☐ yes ☐ no
The subject meets none of the exclusion criteria	☐ yes ☐ no
The caregiver was informed in proper form about the study	☐ yes ☐ no
The caregiver has signed the informed consent form	☐ yes ☐ no

In the investigator's opinion, on the basis of the study assessments, is the subject eligible to participate in this trial? ☐ yes ☐ no

Has the child been randomized? ☐ yes ☐ no

Intervention Group:

A	Treatment Group A	☐
B	Treatment Group B	☐

Investigator's signature _____________________ Date (dd/mm/yy) ☐☐ / ☐☐ / ☐☐

VISIT 1 (DAY 0): BASELINE 2/4

CHILD HEALTH STATUS AND DRUG INTAKE SINCE SCREENING

Chronic disease	☐ yes ☐ no If yes, treatment....................
Fever	☐ yes ☐ no If yes, treatment....................
Hospitalization	☐ yes ☐ no If yes, treatment....................
Vomiting for long time	☐ yes ☐ no If yes, treatment....................
Cough or difficult breathing	☐ yes ☐ no If yes, treatment....................
Diagnosis of pneumonia	☐ yes ☐ no If yes, treatment....................
Diagnosis of tuberculosis	☐ yes ☐ no If yes, treatment....................
Gastrointestinal disorders	☐ yes ☐ no If yes, treatment....................
Diarrhea	☐ yes ☐ no If yes, treatment....................
Blood or mucus in the stool	☐ yes ☐ no If yes, treatment....................
Malaria	☐ yes ☐ no If yes, treatment....................
Ear infection	☐ yes ☐ no If yes, treatment....................
Sores in mouth	☐ yes ☐ no If yes, treatment....................
Signs of lethargy / unconsciousness	☐ yes ☐ no If yes, treatment....................

Signs of severe dehydration ☐ yes ☐ no If yes, treatment…………………

Palmar pallor ☐ yes ☐ no If yes, treatment…………………

Jaundice ☐ yes ☐ no If yes, treatment…………………

Any other illness ☐ yes ☐ no If yes, treatment…………………

In case of illness, adverse event form filled: ☐ yes ☐ no

Vitamin-mineral supplements ☐ yes ☐ no if yes, specify: ___________________

Other current medication ☐ yes ☐ no if yes, specify: ___________________

PUBERTAL STAGE

Menstruation started: ☐ yes ☐ no

ANTHROPOMETRICS

Length
1st measurement: ☐☐☐.☐ cm
2nd measurement: ☐☐☐.☐ cm
(3rd measurement: ☐☐☐.☐ cm)
Mean = ☐☐☐.☐ cm

Weight
1st measurement: ☐☐☐.☐ kg
2nd measurement: ☐☐☐.☐ kg
(3rd measurement: ☐☐☐.☐ kg)
Mean = ☐☐☐.☐ kg

BLOOD SAMPLING & ANALYSIS

Time blood sampling ☐☐:☐☐

Sample labeling Subject ID___V1:______

Comments about specimen collection/absence/rescheduling ____

Date full blood count	☐☐/☐☐/☐☐☐☐
Hemoglobin (from full blood count)	g/dL ☐☐.☐
MCV (from full blood count)	fL ☐☐.☐

STOOL SAMPLING

Date of stool sampling	☐☐/☐☐
Time stool sampling	☐☐:☐☐
Date of stool processing	☐☐/☐☐
Time of processing	
Sample labeling	Subject ID___V1:______
Comments about specimen collection/absence/rescheduling	____

SUPPLEMENT / SYMPTOMS DIARY DISTRIBUTION

Iron supplement distributed ☐ yes ☐ no

Treatment supplement distributed ☐ yes ☐ no

Treatment code: ☐ A ☐ B

Symptoms diary explained and distributed ☐ yes ☐ no

REMINDER FOR NEXT VISIT

Next visit (visit 2 on day 42 (±3): date (dd/mm/yy) ☐☐/☐☐/☐☐

Time of next visit: ☐☐:☐☐

Investigator’s signature ____________________ Date (dd/mm/yy) ☐☐/☐☐/☐☐

VISIT 2 (DAY 42 ±3): MIDPOINT 3/4

Symptoms diary collected ☐ yes ☐ no

Symptoms diary complete ☐ yes ☐ no ☐ partially

CHILD HEALTH STATUS AND DRUG INTAKE SINCE VISIT 1

Chronic disease	☐ yes ☐ no If yes, treatment…………………
Fever	☐ yes ☐ no If yes, treatment…………………
Hospitalization	☐ yes ☐ no If yes, treatment…………………
Vomiting for long time	☐ yes ☐ no If yes, treatment…………………
Cough or difficult breathing	☐ yes ☐ no If yes, treatment…………………
Diagnosis of pneumonia	☐ yes ☐ no If yes, treatment…………………
Diagnosis of tuberculosis	☐ yes ☐ no If yes, treatment…………………
Gastrointestinal disorders	☐ yes ☐ no If yes, treatment…………………
Diarrhea	☐ yes ☐ no If yes, treatment…………………
Blood or mucus in the stool	☐ yes ☐ no If yes, treatment…………………
Malaria	☐ yes ☐ no If yes, treatment…………………
Ear infection	☐ yes ☐ no If yes, treatment…………………
Sores in mouth	☐ yes ☐ no If yes, treatment…………………
Signs of lethargy / unconsciousness	☐ yes ☐ no If yes, treatment…………………
Signs of severe dehydration	☐ yes ☐ no If yes, treatment…………………
Palmar pallor	☐ yes ☐ no If yes, treatment…………………
Jaundice	☐ yes ☐ no If yes, treatment…………………
Any other illness	☐ yes ☐ no If yes, treatment…………………

In case of illness, adverse event form filled: ☐ yes ☐ no

Vitamin-mineral supplements ☐ yes ☐ no if yes, specify: ____________________

Other current medication ☐ yes ☐ no if yes, specify: ____________________

PUBERTAL STAGE

Menstruation started: ☐ yes ☐ no

ANTHROPOMETRICS

Length
1st measurement: ☐☐☐.☐ cm
2nd measurement: ☐☐☐.☐ cm
(3rd measurement: ☐☐☐.☐ cm)
Mean = ☐☐☐.☐ cm

Weight
1st measurement: ☐☐☐.☐ kg
2nd measurement: ☐☐☐.☐ kg
(3rd measurement: ☐☐☐.☐ kg)
Mean = ☐☐☐.☐ kg

BLOOD SAMPLING & ANALYSIS

Time blood sampling	☐☐:☐☐
Sample labeling	Subject ID___V2:______
Comments about specimen collection/absence/rescheduling	____
Date full blood count	☐☐/☐☐/☐☐☐☐
Hemoglobin (from full blood count)	g/dL ☐☐.☐
MCV (from full blood count)	fL ☐☐.☐

SUPPLEMENT / SYMPTOMS DIARY DISTRIBUTION

Iron supplement distributed ☐ yes ☐ no

Treatment supplement distributed ☐ yes ☐ no

Treatment code: ☐ A ☐ B

Symptoms diary explained and distributed ☐ yes ☐ no

REMINDER FOR NEXT VISIT

Next visit (visit 3 on day 84 (±3): date (dd/mm/yy) ☐☐/☐☐/☐☐

Time of next visit: ☐☐:☐☐

Investigator's signature ____________________Date (dd/mm/yy) ☐☐/☐☐/☐☐

VISIT 13 (DAY 84 ± 3): ENDPOINT 4/4

Symptoms diary collected ☐ yes ☐ no

Symptoms diary complete ☐ yes ☐ no ☐ partially

CHILD HEALTH STATUS AND DRUG INTAKE SINCE MIDPOINT

Chronic disease	☐ yes ☐ no If yes, treatment…………………
Fever	☐ yes ☐ no If yes, treatment…………………
Hospitalization	☐ yes ☐ no If yes, treatment…………………
Vomiting for long time	☐ yes ☐ no If yes, treatment…………………
Cough or difficult breathing	☐ yes ☐ no If yes, treatment…………………
Diagnosis of pneumonia	☐ yes ☐ no If yes, treatment…………………

Diagnosis of tuberculosis	☐ yes ☐ no	If yes, treatment…………………
Gastrointestinal disorders	☐ yes ☐ no	If yes, treatment…………………
Diarrhea	☐ yes ☐ no	If yes, treatment…………………
Blood or mucus in the stool	☐ yes ☐ no	If yes, treatment…………………
Malaria	☐ yes ☐ no	If yes, treatment…………………
Ear infection	☐ yes ☐ no	If yes, treatment…………………
Sores in mouth	☐ yes ☐ no	If yes, treatment…………………
Signs of lethargy / unconsciousness	☐ yes ☐ no	If yes, treatment…………………
Signs of severe dehydration	☐ yes ☐ no	If yes, treatment…………………
Palmar pallor	☐ yes ☐ no	If yes, treatment…………………
Jaundice	☐ yes ☐ no	If yes, treatment…………………
Any other illness	☐ yes ☐ no	If yes, treatment…………………

In case of illness, adverse event form filled: ☐ yes ☐ no

Vitamin-mineral supplements ☐ yes ☐ no if yes, specify: ____________________

Other current medication ☐ yes ☐ no if yes, specify: ____________________

PUBERTAL STAGE

Menstruation started: ☐ yes ☐ no

ANTHROPOMETRICS

Length	1st measurement: ☐☐☐.☐ cm
	2nd measurement: ☐☐☐.☐ cm
	(3rd measurement: ☐☐☐.☐ cm)
	Mean = ☐☐☐.☐ cm
Weight	1st measurement: ☐☐☐.☐ kg
	2nd measurement: ☐☐☐.☐ kg
	(3rd measurement: ☐☐☐.☐ kg)

Mean = ☐☐☐.☐ kg

BLOOD SAMPLING & ANALYSIS

Time blood sampling	☐☐:☐☐
Sample labeling	Subject ID___V3:______
Comments about specimen collection/absence/rescheduling	____
Date full blood count	☐☐/☐☐/☐☐☐☐
Hemoglobin (from full blood count)	g/dL ☐☐.☐
MCV (from full blood count)	fL ☐☐.☐

STOOL SAMPLING

Date of stool sampling	☐☐/☐☐
Time stool sampling	☐☐:☐☐
Date of stool processing	☐☐/☐☐
Time of processing	
Sample labeling	Subject ID___V1:______
Comments about specimen collection/absence/rescheduling	____

Investigator’s signature __________________ Date (dd/mm/yy) ☐☐ / ☐☐ / ☐☐

FINAL INFORMATION

Did subject complete study per protocol?

☐ Yes, indicate date of completion (dd/mm/yy) ☐☐ / ☐☐ / ☐☐

☐ No, indicate date of termination / withdrawal (dd/mm/yy) ☐☐ / ☐☐ / ☐☐

If no:

Specify reason for withdrawal /dropout (check one only):

Consent withdrawn ☐

Non-compliance ☐

Adverse event/Serious adverse event ☐

Episode of severe illness ☐

Other, specify: ________________ ☐

Last day of contact with the subject (dd/mm/yy) ☐☐ / ☐☐ / ☐☐

I have reviewed the case report forms for this subject and verified that they are accurate and complete

Investigator’s signature __________________ Date (dd/mm/yy) ☐☐ / ☐☐ / ☐☐

Eidgenössische Technische Hochschule Zürich
Swiss Federal Institute of Technology Zurich

Appendix 4

Improving efficacy and safety of oral iron supplementation in HIV-infected children by providing prebiotic galacto-oligosaccharides as adjunct treatment: A randomized controlled trial

WEEKLY SUPPLEMENT INTAKE & SYMPTOMS DIARY

Subject ID ☐☐☐ **Study week** ☐☐

*Indicate what your child experienced by **ticking √ the symptoms** for the day and **whether child took supplements/any medicine**. Please remember to fill in the **date***

**Day**	**Sunday**		**Monday**		**Tuesday**		**Wednesday**		**Thursday**		**Friday**		**Saturday**	
**Date** (dd/mm/yyyy):														
**Healthy**														
**Diarrhea**														
**Runny nose**														
**Coughing**														
**Chest pain**														
**Fever**														
**Nauseous**														
**Vomiting**														
**Tummy ache**														

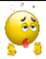 Very tired														
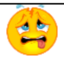 Headache														
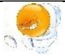 Constipation														
**Did the child take iron supplement?**														
**Did the child drink the GOS/placebo powder?**														
**Did the child get any medicine or traditional medicine?** Please write them down														

ETH

Eidgenössische Technische Hochschule Zürich
Swiss Federal Institute of Technology Zurich

Appendix 5

Improving efficacy and safety of oral iron supplementation in HIV-infected children by providing prebiotic galacto-oligosaccharides as adjunct treatment: A randomized controlled trial

Socio-demographic questionnaire – Caregiver/child

Subject ID | | | | **Date:** | 2 | 0 | Y | Y | M | M | D | D | **Investigator:** ____________________

Question									
1. Date of birth:	Y	Y	Y	Y	M	M	D	D	

Question						
		1	2	3	4	
2. What is your relationship with the child (participant)?		Mother	Father	Grandmother	Aunt	Other. Specify:
		1	2	3	4	5
3. How would you describe your child in terms of population group?		Black	Coloured	Indian	White	Other. Specify:
		1	2	3	4	5
4. What is your home language?		English	isiXhosa	Afrikaans	Zulu	Other. Specify:
		1	2	3	4	5
5. Highest education level of primary caregiver (stays in same household as child)? *Choose the parent with the highest education		None	Primary School	Gr 8-9	Gr 10-12	Tertiary Education (college /university)
		1	2	3	4	5
6. Marital status of caregiver?		Unmarried, living with partner	Unmarried and not living with partner	Married and living with husband	Married and not living with husband	Other. Specify:
	1	2	3	4	5	6

7. Current employment status of breadwinner	Permanent employment	Seasonal worker	Day-to-day basis	Self-employed	Unemployed	Other. Specify:
8. How many people live in your household most days of the week (including children and elderly)?	______________________________					
	1	2	3	4	5	6
9. Do any members of the household receive any grants?	None	Child support	Foster care	Disability	Old age pension	Other. Specify

10. To determine your living standards measure, please indicate which of the following you currently have in your household:

X = Yes ; - = No

X	Metropolitan dweller (250 000+)		DVD Player / Blu Ray Player
	~~Living in a non-urban area~~		Refrigerator or combined fridge/freezer
	House / Cluster House / Town House		Electric Stove
	Tap water in house / on plot		Microwave oven
	Flush Toilet inside house		Deep Freezer - Free Standing
	Hot running water		Washing machine
	Built in Kitchen Sink		Tumble dryer
	No Domestic Workers or Gardeners		Dishwashing Machine
	Home security service		PayTV (M-net / DSTV / TopTV) Subscription
	2 Cell phones in Household		Home Theatre System
	3 or more Cell phones in Household		Vacuum Cleaner
	Zero or One Radio set in Household		Motor Vehicle
	Air conditioner (excl. fans)		Computer - Desktop / Laptop
	Have TV set(s)		Land line (excl. Cellphone)
	Swimming Pool		
